# Supplementary material for: A Bayesian Model Based on Local Phenotypic Resistance Data to Inform Empiric Antibiotic Escalation Decisions
Source: Infect Dis Ther. 2024 Jul 18;13(9):1963–81. doi: 10.1007/s40121-024-01011-3 (PMC11343932; doi:10.1007/s40121-024-01011-3)
Supplement: Supplementary file 1 — Supplementary file1 (PDF 4731 KB) [file 40121_2024_1011_MOESM1_ESM.pdf]

Rational Empiric Antibiotic Escalation Applied to Specific Patient Groups Based on Local Phenotypic Resistance Data

Ranjeet Bamber

Department of Population Health Sciences, Bristol Medical School, Faculty of Health Sciences, University of Bristol, Bristol. UK

Brian Sullivan

Department of Population Health Sciences, Bristol Medical School, Faculty of Health Sciences, University of Bristol, Bristol. UK

Léo Gorman

Jean Golding Institute, University of Bristol, Bristol. UK

Winnie WY Lee

School of Cellular and Molecular Medicine, Faculty of Life Sciences, University of Bristol, Bristol. UK

Matthew B Avison

School of Cellular and Molecular Medicine, Faculty of Life Sciences, University of Bristol, Bristol. UK

Andrew W Dowsey

Department of Population Health Sciences, Bristol Medical School, Faculty of Health Sciences, University of Bristol

Philip B Williams*

University Hospitals Bristol and Weston NHS Foundation Trust, Bristol Royal Infirmary, Bristol. UK

*** Corresponding Author**

Philip.Williams2@UHBW.nhs.uk

Supplementary Material (AMR Percentages)

Antibiotic resistance over time, with the probability of decreasing resistance between the start and end points (All patients)

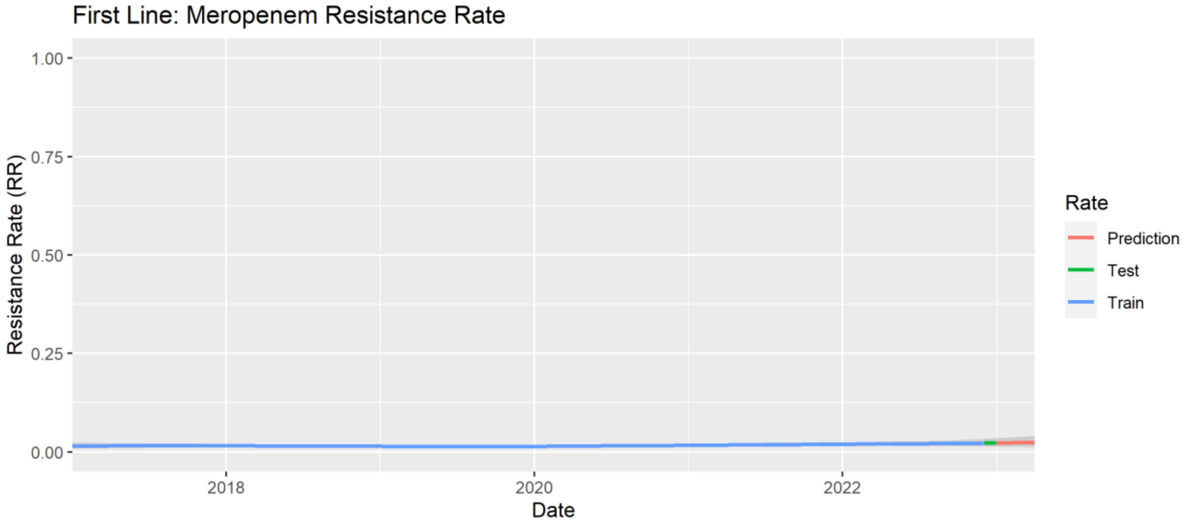

[1] "2017-01-01 Mid 0.015 (95%CI 0.008 to 0.024) Estimated Error 0.0039"

[1] "2022-12-02 Mid 0.022 (95%CI 0.014 to 0.033) Estimated Error 0.0050"

[1] "2022-12-31 Mid 0.022 (95%CI 0.013 to 0.035) Estimated Error 0.0055"

11.3% posterior probability of decrease/superiority

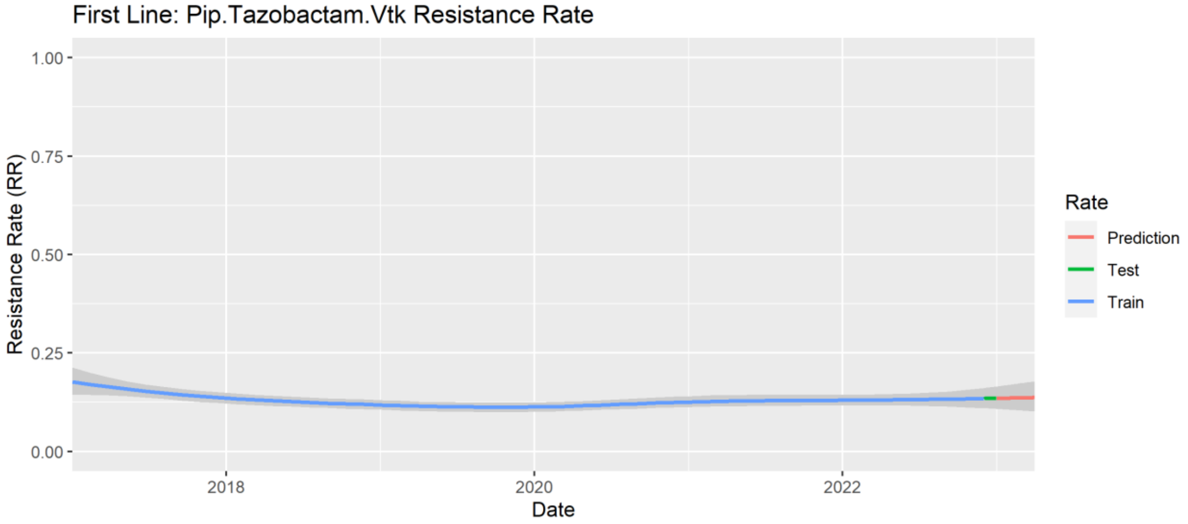

[1] "2017-01-01 Mid 0.176 (95%CI 0.145 to 0.213) Estimated Error 0.0171"

[1] "2022-12-02 Mid 0.134 (95%CI 0.110 to 0.161) Estimated Error 0.0130"

[1] "2022-12-31 Mid 0.135 (95%CI 0.108 to 0.164) Estimated Error 0.0144"

97.9% posterior probability of decrease/superiority

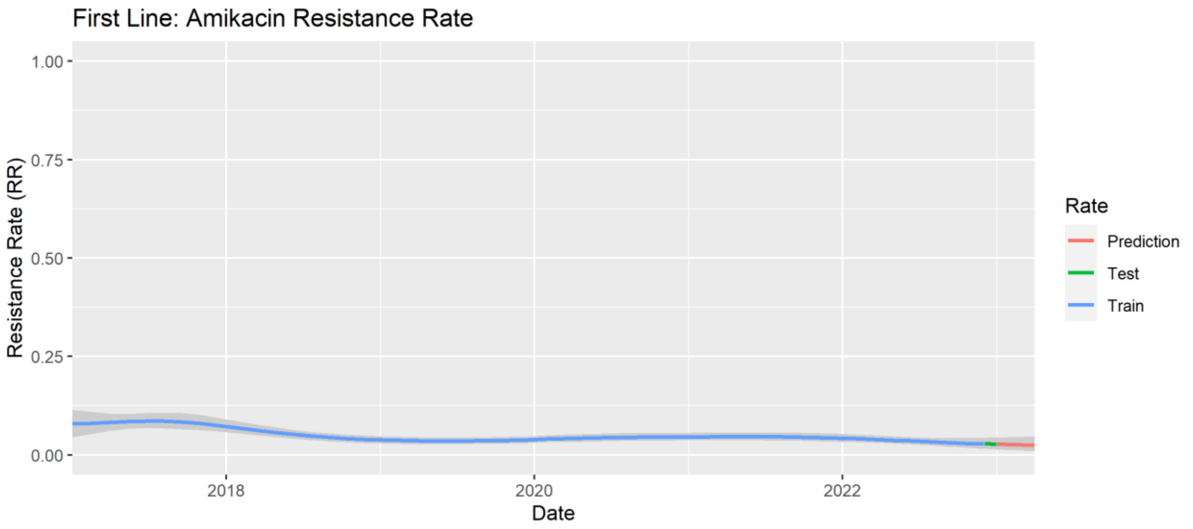

[1] "2017-01-01 Mid 0.079 (95%CI 0.045 to 0.115) Estimated Error 0.0179"

[1] "2022-12-03 Mid 0.028 (95%CI 0.016 to 0.043) Estimated Error 0.0072"

[1] "2022-12-31 Mid 0.027 (95%CI 0.014 to 0.044) Estimated Error 0.0077"

99.7% posterior probability of decrease/superiority

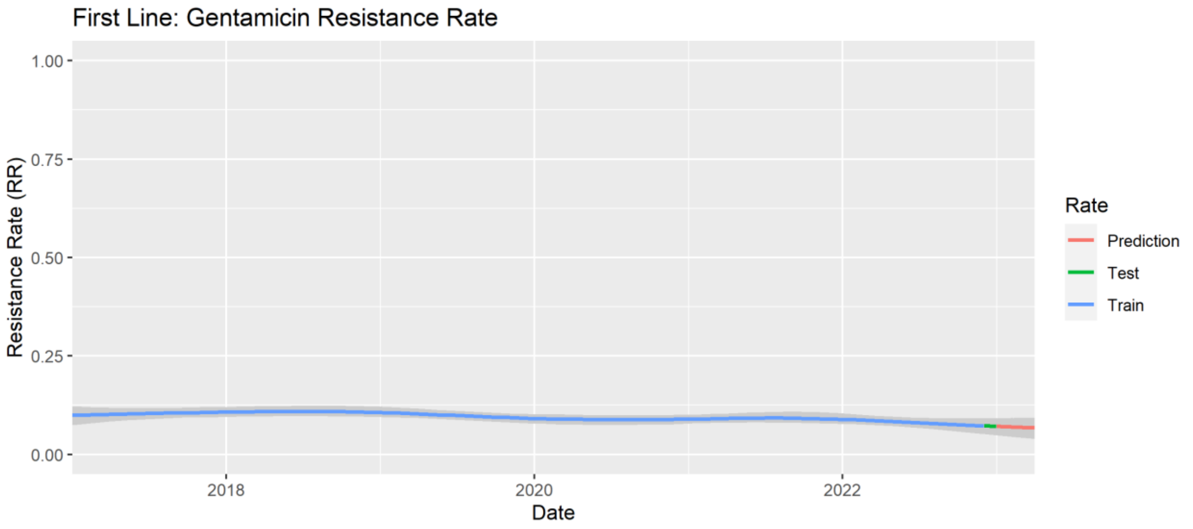

[1] "2017-01-01 Mid 0.099 (95%CI 0.075 to 0.122) Estimated Error 0.0123"

[1] "2022-12-02 Mid 0.072 (95%CI 0.052 to 0.091) Estimated Error 0.0103"

[1] "2022-12-31 Mid 0.071 (95%CI 0.048 to 0.091) Estimated Error 0.0112"

97.2% posterior probability of decrease/superiority

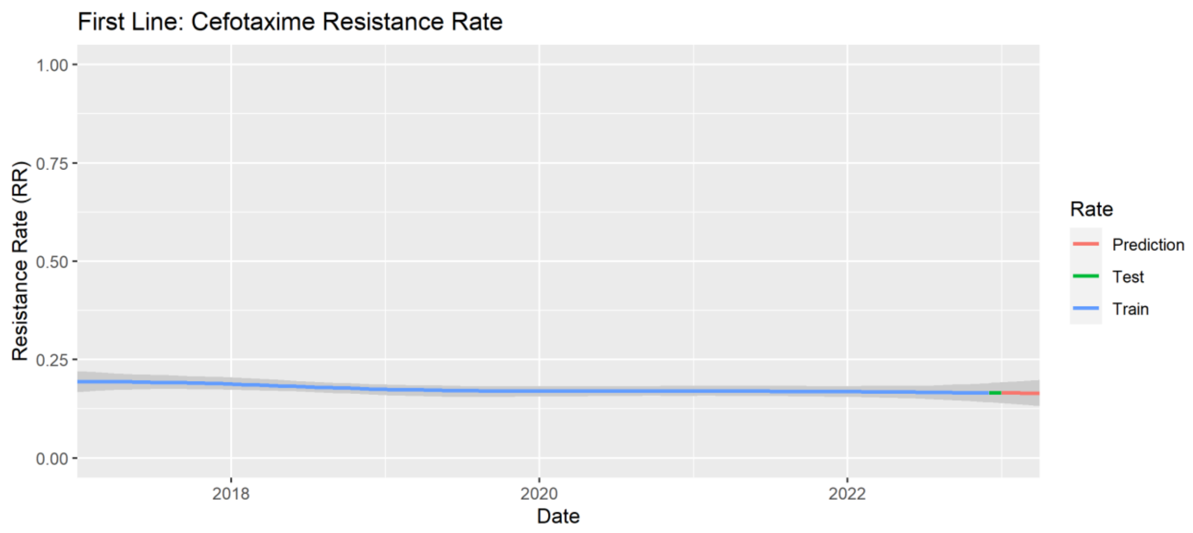

[1] "2017-01-01 Mid 0.194 (95%CI 0.168 to 0.221) Estimated Error 0.0135"

[1] "2022-12-02 Mid 0.165 (95%CI 0.142 to 0.190) Estimated Error 0.0121"

[1] "2022-12-31 Mid 0.165 (95%CI 0.139 to 0.192) Estimated Error 0.0130"

94.6% posterior probability of decrease/superiority

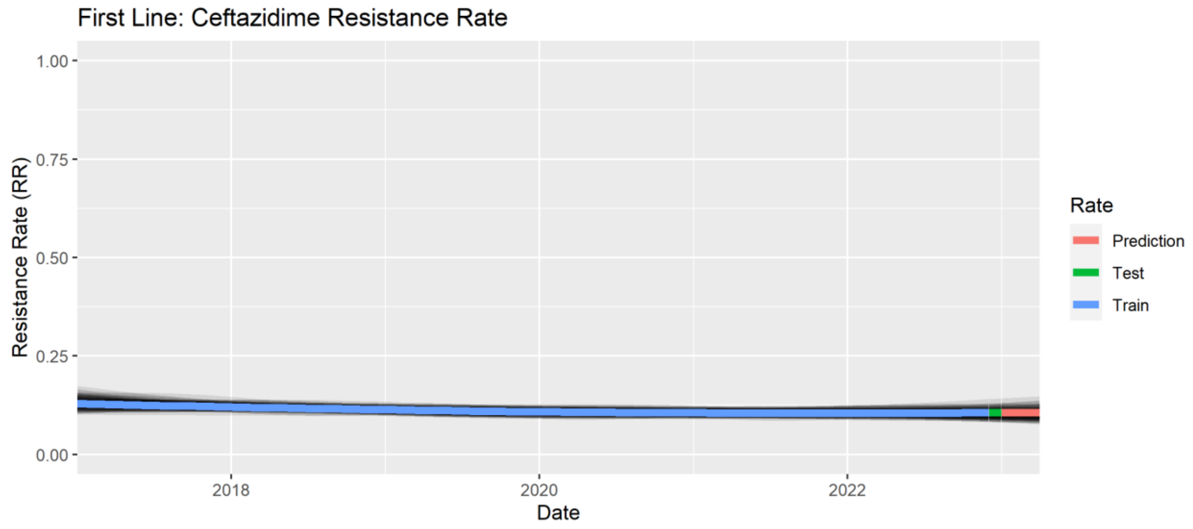

[1] "2017-01-01 Mid 0.129 (95%CI 0.109 to 0.153) Estimated Error 0.0112"

[1] "2022-12-02 Mid 0.106 (95%CI 0.089 to 0.128) Estimated Error 0.0097"

[1] "2022-12-31 Mid 0.106 (95%CI 0.088 to 0.129) Estimated Error 0.0103"

94.3% posterior probability of decrease/superiority

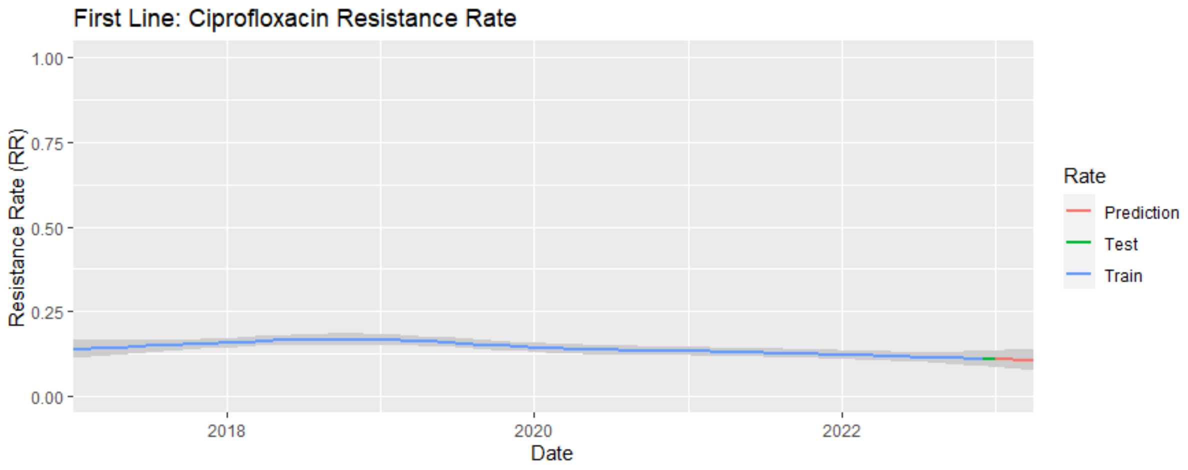

[1] "2017-01-01 Mid 0.139 (95%CI 0.111 to 0.168) Estimated Error 0.0144"

[1] "2022-12-02 Mid 0.109 (95%CI 0.086 to 0.132) Estimated Error 0.0115"

[1] "2022-12-31 Mid 0.108 (95%CI 0.083 to 0.133) Estimated Error 0.0125"

95.1% posterior probability of decrease/superiority

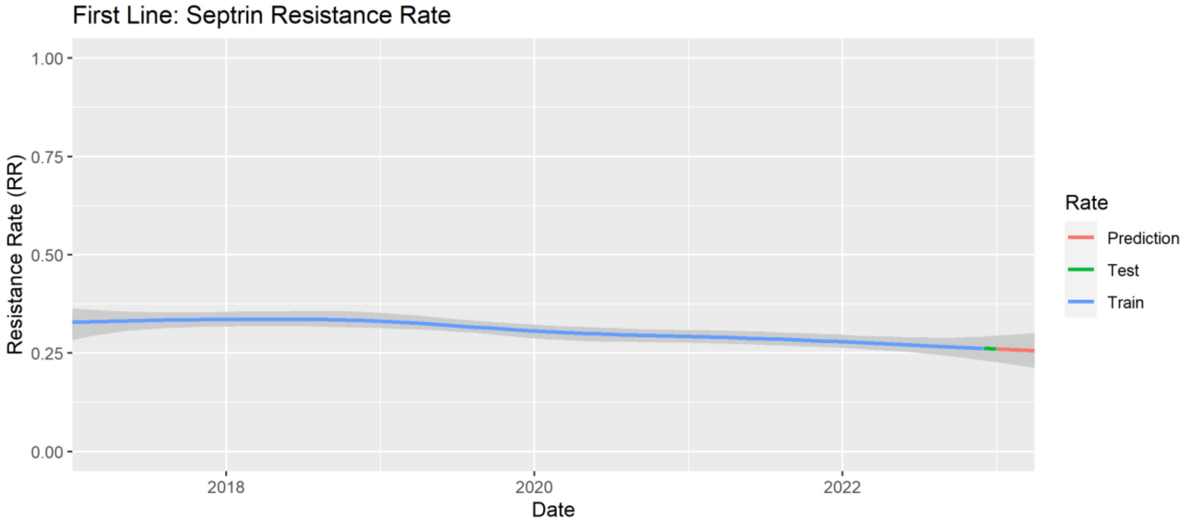

[1] "2017-01-01 Mid 0.329 (95%CI 0.283 to 0.364) Estimated Error 0.0203"

[1] "2022-12-02 Mid 0.262 (95%CI 0.231 to 0.292) Estimated Error 0.0153"

[1] "2022-12-31 Mid 0.260 (95%CI 0.227 to 0.294) Estimated Error 0.0167"

98.5% posterior probability of decrease/superiority

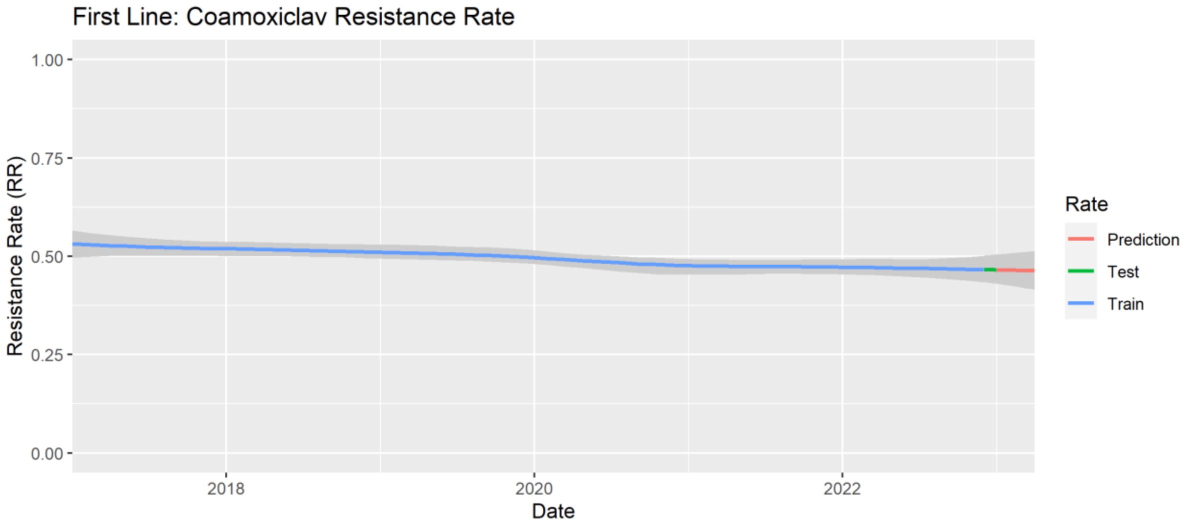

[1] "2017-01-01 Mid 0.531 (95%CI 0.497 to 0.566) Estimated Error 0.0169"

[1] "2022-12-02 Mid 0.466 (95%CI 0.434 to 0.502) Estimated Error 0.0169"

[1] "2022-12-31 Mid 0.465 (95%CI 0.430 to 0.504) Estimated Error 0.0183"

99.1% posterior probability of decrease/superiority

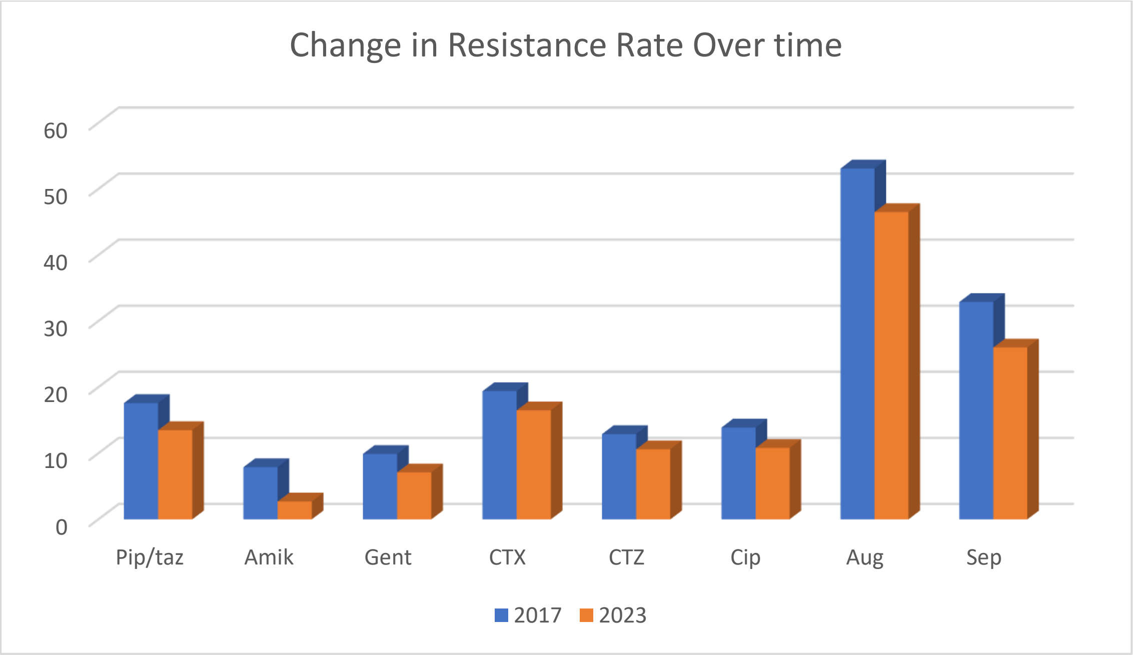

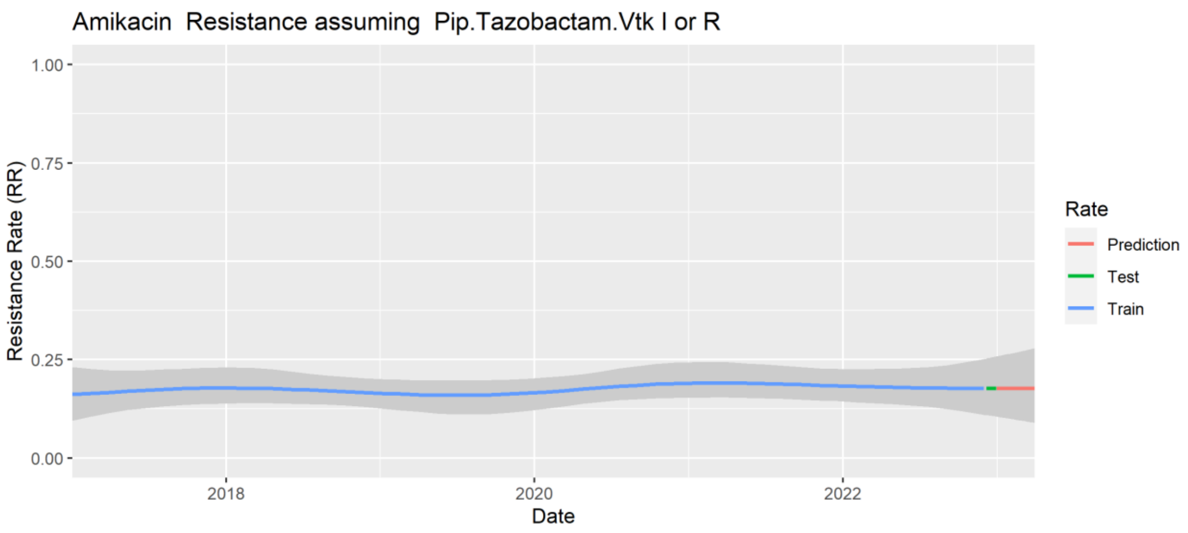

[1] "2017-01-02 Mid 0.161 (95%CI 0.095 to 0.231) Estimated Error 0.0341"

[1] "2022-12-06 Mid 0.177 (95%CI 0.109 to 0.253) Estimated Error 0.0358"

[1] "2022-12-30 Mid 0.177 (95%CI 0.105 to 0.258) Estimated Error 0.0380"

"37.0% posterior probability of decrease "

All patients

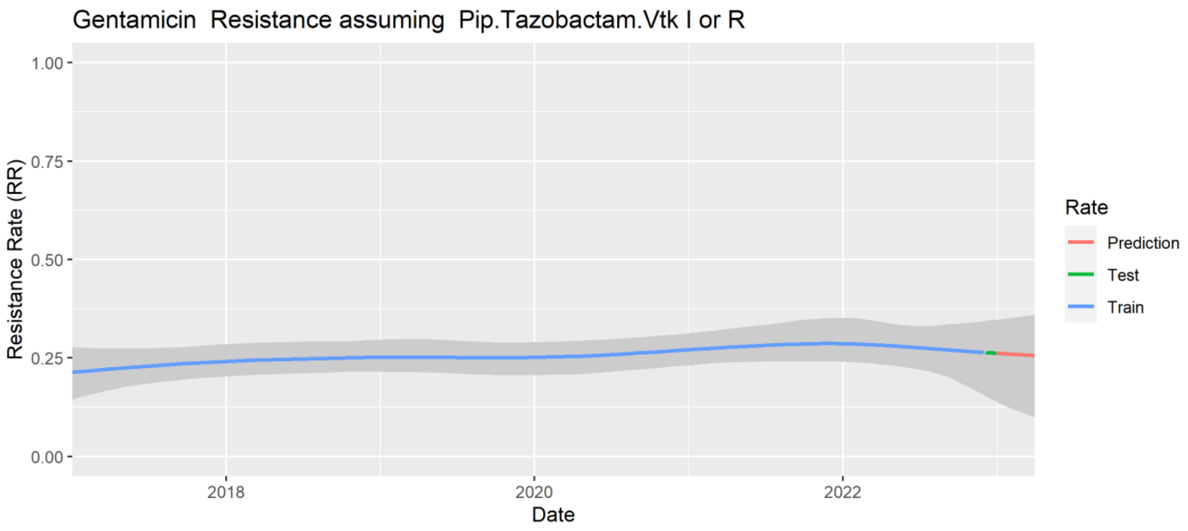

[1] "2017-01-01 Mid 0.214 (95%CI 0.145 to 0.278) Estimated Error 0.0333"

[1] "2022-12-06 Mid 0.263 (95%CI 0.153 to 0.344) Estimated Error 0.0466"

[1] "2022-12-30 Mid 0.262 (95%CI 0.139 to 0.347) Estimated Error 0.0500"

"16.8% posterior probability of decrease "

All patients

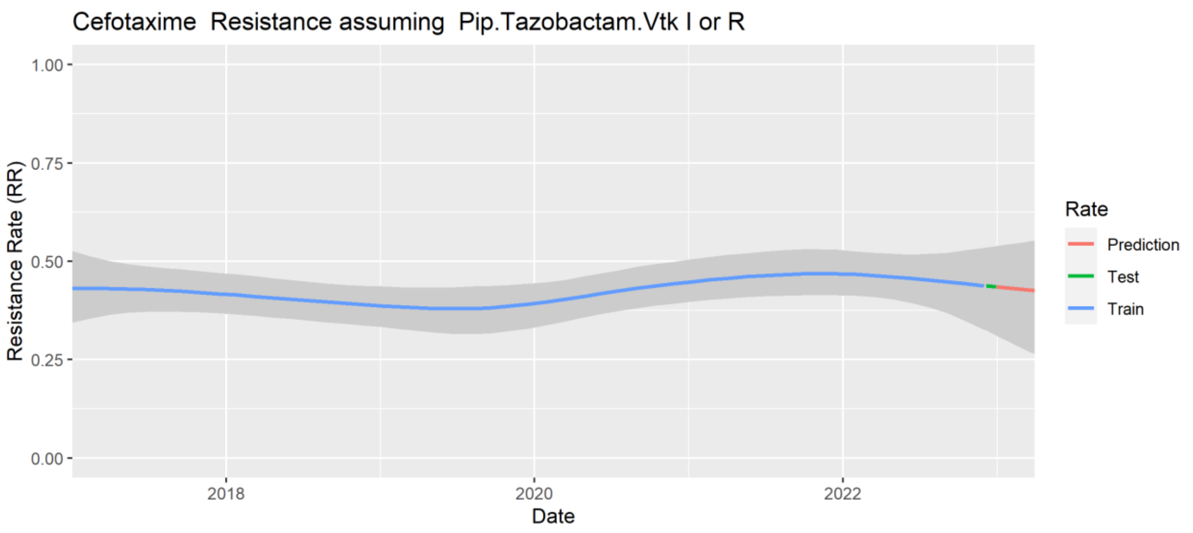

[1] "2017-01-01 Mid 0.432 (95%CI 0.344 to 0.526) Estimated Error 0.0461"

[1] "2022-12-06 Mid 0.437 (95%CI 0.324 to 0.534) Estimated Error 0.0535"

[1] "2022-12-30 Mid 0.435 (95%CI 0.311 to 0.538) Estimated Error 0.0577"

"44.4% posterior probability of decrease "

All patients

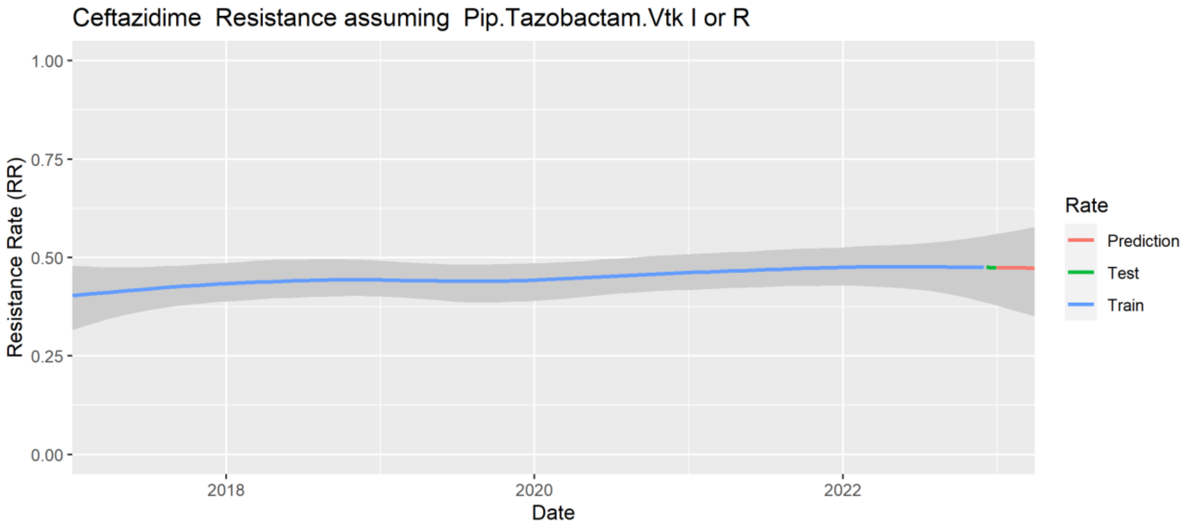

[1] "2017-01-01 Mid 0.404 (95%CI 0.316 to 0.479) Estimated Error 0.0407"

[1] "2022-12-06 Mid 0.475 (95%CI 0.385 to 0.555) Estimated Error 0.0423"

[1] "2022-12-30 Mid 0.474 (95%CI 0.378 to 0.559) Estimated Error 0.0448"

"10.6% posterior probability of decrease "

All patients

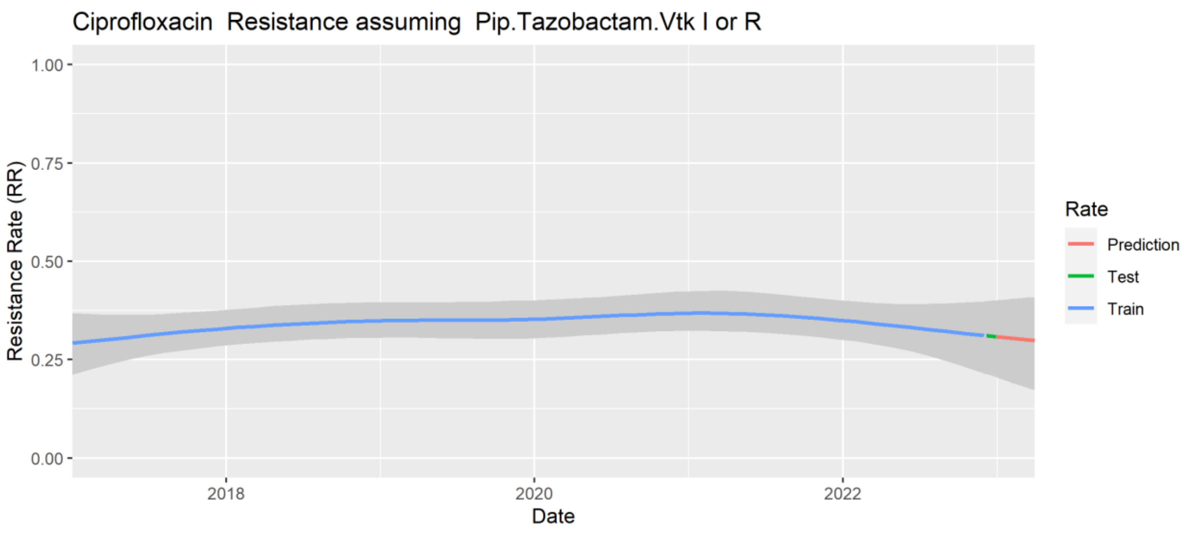

[1] "2017-01-01 Mid 0.292 (95%CI 0.212 to 0.368) Estimated Error 0.0398"

[1] "2022-12-06 Mid 0.311 (95%CI 0.213 to 0.399) Estimated Error 0.0484"

[1] "2022-12-30 Mid 0.308 (95%CI 0.205 to 0.400) Estimated Error 0.0512"

[1] "36.5% posterior probability of decrease "

All patients

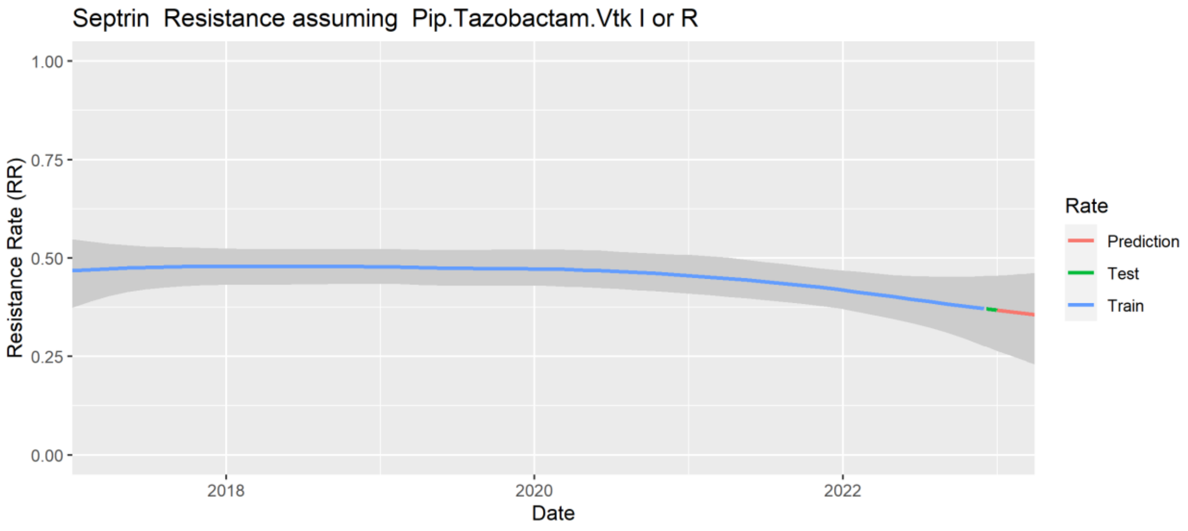

[1] "2017-01-01 Mid 0.468 (95%CI 0.374 to 0.547) Estimated Error 0.0438"

[1] "2022-12-06 Mid 0.371 (95%CI 0.275 to 0.454) Estimated Error 0.0459"

[1] "2022-12-30 Mid 0.368 (95%CI 0.265 to 0.455) Estimated Error 0.0486"

[1] "94.6% posterior probability of decrease "

All patients

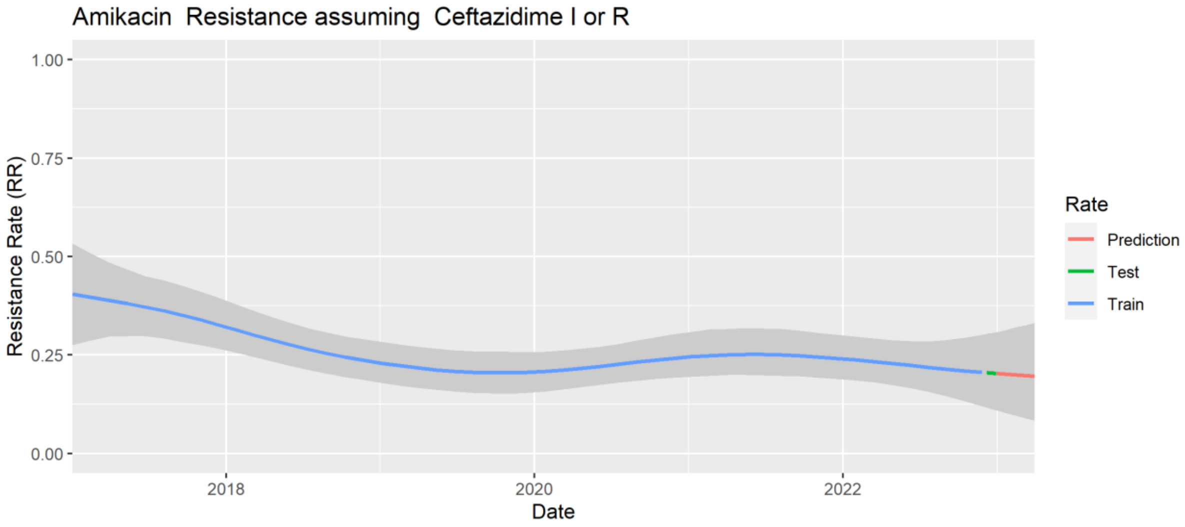

[1] "2017-01-02 Mid 0.404 (95%CI 0.275 to 0.532) Estimated Error 0.0649"

[1] "2022-12-07 Mid 0.205 (95%CI 0.116 to 0.304) Estimated Error 0.0476"

[1] "2022-12-30 Mid 0.203 (95%CI 0.109 to 0.307) Estimated Error 0.0506"

[1] "99.0% posterior probability of decrease "

All patients

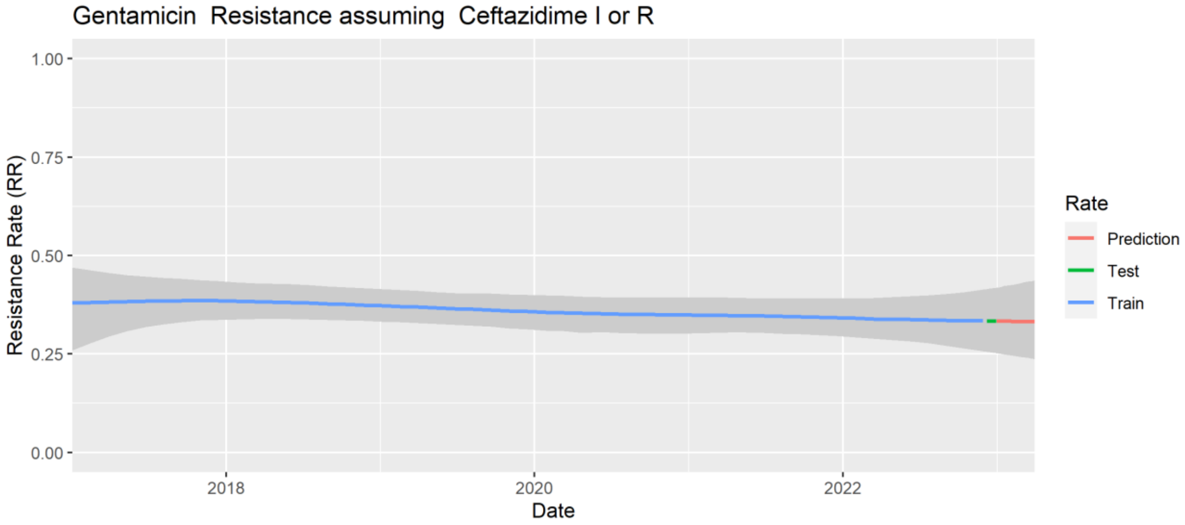

[1] "2017-01-01 Mid 0.380 (95%CI 0.259 to 0.469) Estimated Error 0.0494"

[1] "2022-12-07 Mid 0.334 (95%CI 0.256 to 0.415) Estimated Error 0.0401"

[1] "2022-12-30 Mid 0.333 (95%CI 0.252 to 0.418) Estimated Error 0.0419"

"77.9% posterior probability of decrease "

All patients

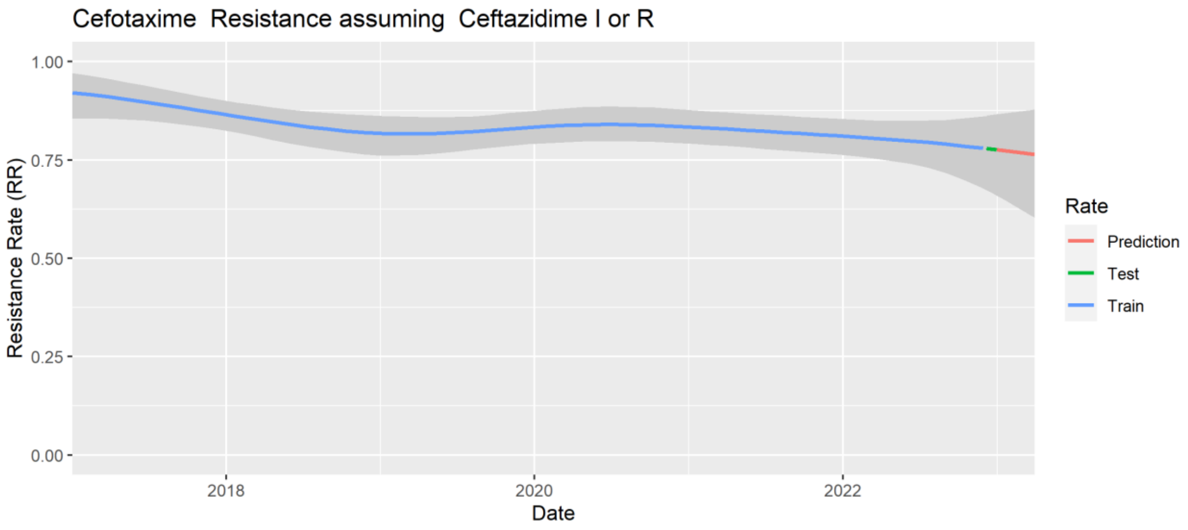

[1] "2017-01-01 Mid 0.921 (95%CI 0.855 to 0.971) Estimated Error 0.0303"

[1] "2022-12-07 Mid 0.779 (95%CI 0.673 to 0.862) Estimated Error 0.0480"

[1] "2022-12-30 Mid 0.776 (95%CI 0.660 to 0.866) Estimated Error 0.0520"

[1] "99.5% posterior probability of decrease "

All patients

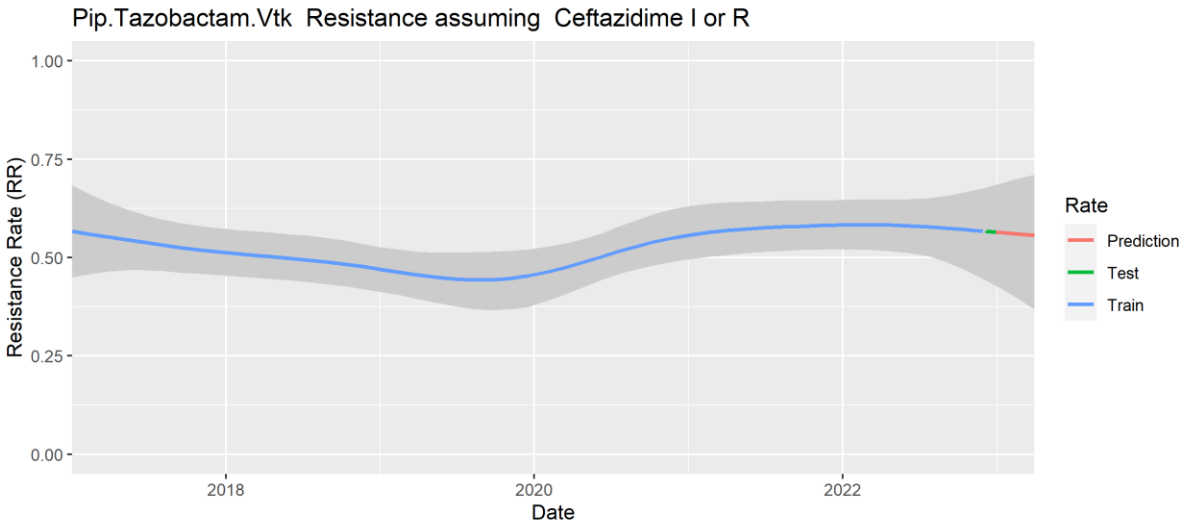

[1] "2017-01-01 Mid 0.567 (95%CI 0.450 to 0.683) Estimated Error 0.0600"

[1] "2022-12-07 Mid 0.566 (95%CI 0.441 to 0.678) Estimated Error 0.0603"

[1] "2022-12-30 Mid 0.564 (95%CI 0.428 to 0.684) Estimated Error 0.0654"

[1] "49.2% posterior probability of decrease "

All patients

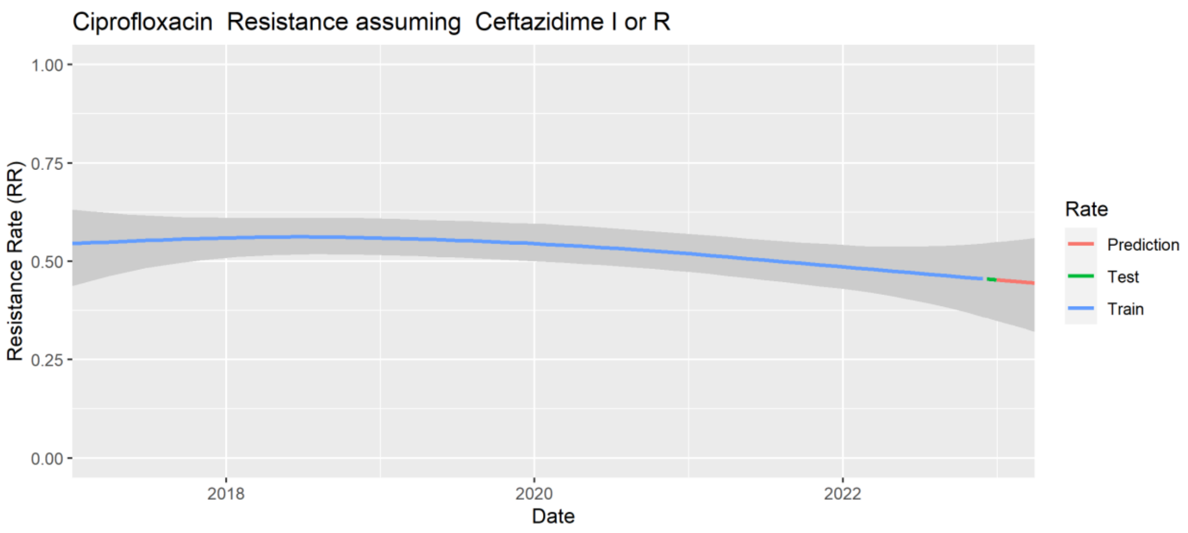

[1] "2017-01-01 Mid 0.545 (95%CI 0.437 to 0.632) Estimated Error 0.0502"

[1] "2022-12-07 Mid 0.455 (95%CI 0.356 to 0.546) Estimated Error 0.0476"

[1] "2022-12-30 Mid 0.453 (95%CI 0.349 to 0.549) Estimated Error 0.0498"

[1] "90.7% posterior probability of decrease "

All patients

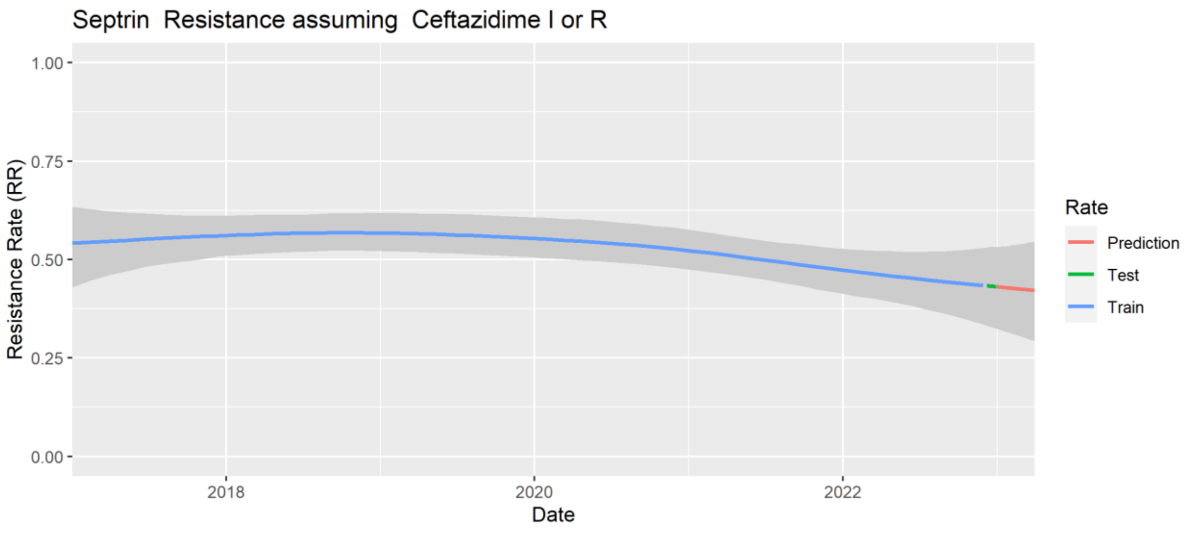

[1] "2017-01-01 Mid 0.542 (95%CI 0.430 to 0.634) Estimated Error 0.0519"

[1] "2022-12-07 Mid 0.433 (95%CI 0.332 to 0.531) Estimated Error 0.0496"

[1] "2022-12-30 Mid 0.431 (95%CI 0.324 to 0.532) Estimated Error 0.0522"

[1] "94.2% posterior probability of decrease "

All patients

Supplementary Material (Isolate Percentages)

Isolate Percentages Over time.

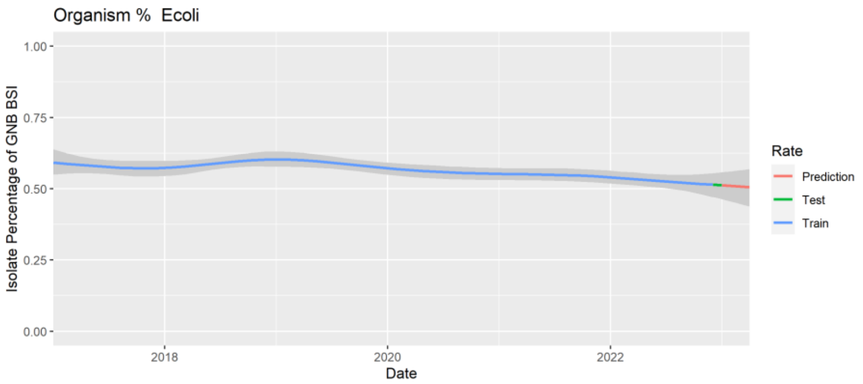

[1] "2017-01-01 Mid 0.592 (95%CI 0.550 to 0.638) Estimated Error 0.0224"

[1] "2022-12-02 Mid 0.514 (95%CI 0.471 to 0.554) Estimated Error 0.0210"

[1] "2022-12-31 Mid 0.512 (95%CI 0.463 to 0.557) Estimated Error 0.0236"

99.5% Posterior Probability of decrease from 2017 to 2023

E. coli   All patients

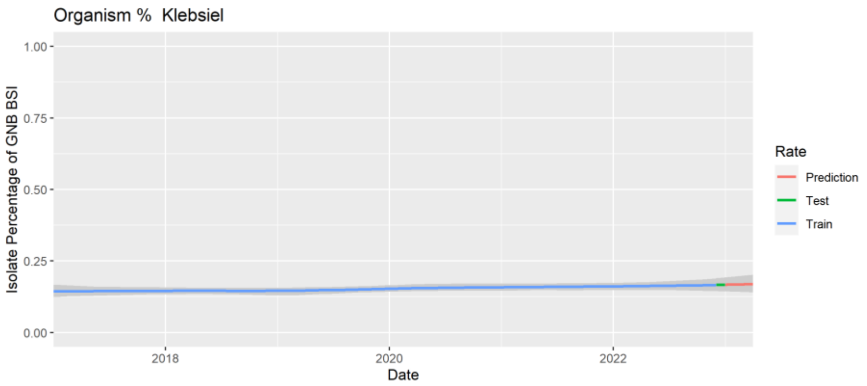

[1] "2017-01-01 Mid 0.144 (95%CI 0.125 to 0.167) Estimated Error 0.0108"

[1] "2022-12-02 Mid 0.166 (95%CI 0.144 to 0.190) Estimated Error 0.0113"

[1] "2022-12-31 Mid 0.167 (95%CI 0.144 to 0.192) Estimated Error 0.0122"

8.2% x greater than y

Klebsiella sp. All patients

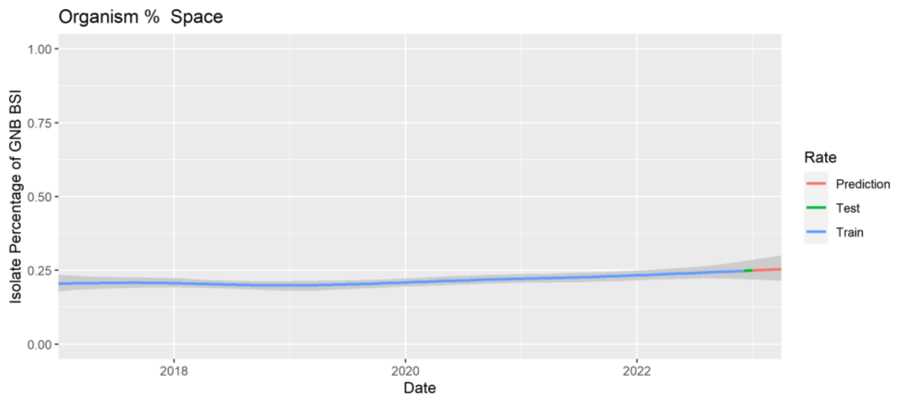

[1] "2017-01-01 Mid 0.205 (95%CI 0.179 to 0.235) Estimated Error 0.0143"

[1] "2022-12-02 Mid 0.248 (95%CI 0.220 to 0.281) Estimated Error 0.0151"

[1] "2022-12-31 Mid 0.250 (95%CI 0.219 to 0.286) Estimated Error 0.0165"

1.8% Posterior Probability of decrease from 2017 to 2023

Space Organisms All patients

##

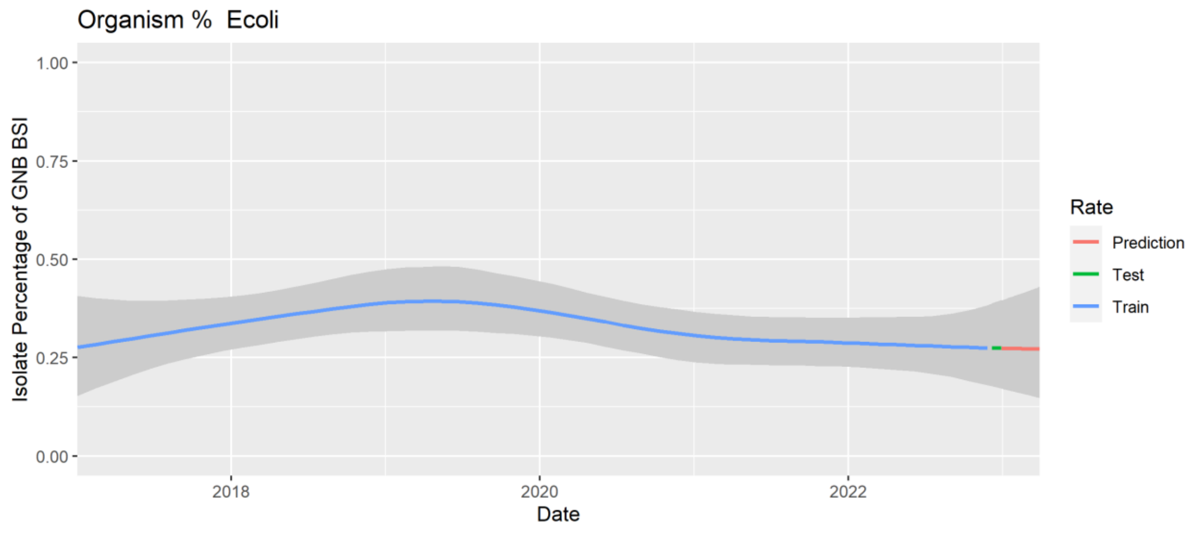

[1] "2017-01-01 Mid 0.276 (95%CI 0.153 to 0.407) Estimated Error 0.0661"

[1] "2022-12-06 Mid 0.274 (95%CI 0.177 to 0.388) Estimated Error 0.0533"

[1] "2022-12-29 Mid 0.274 (95%CI 0.171 to 0.395) Estimated Error 0.0567"

52.0% Posterior Probability of decrease from 2017 to 2023

E. coli ICU

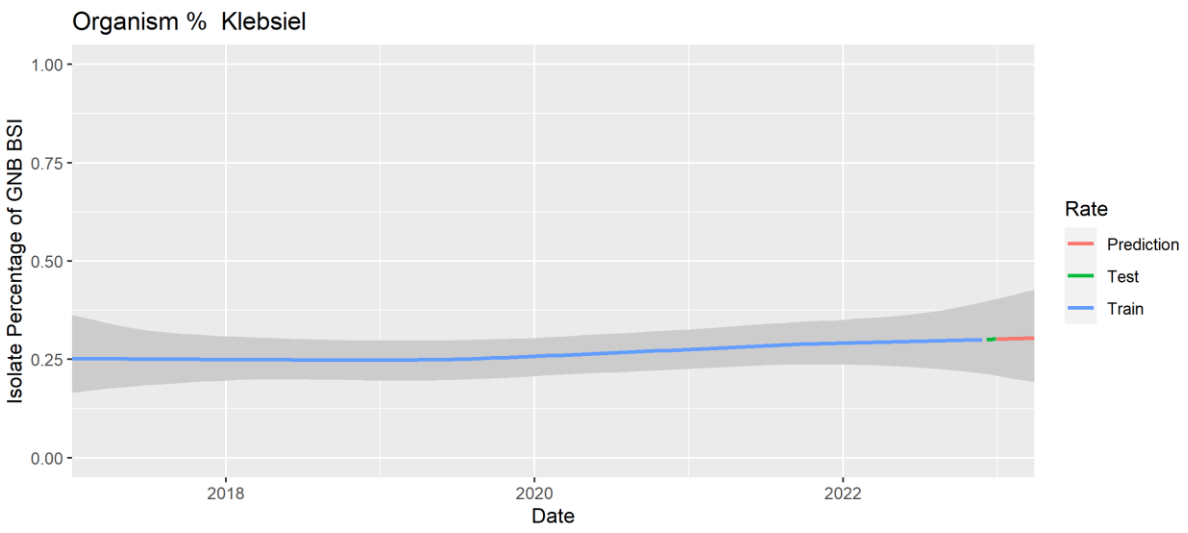

[1] "2017-01-01 Mid 0.252 (95%CI 0.165 to 0.363) Estimated Error 0.0498"

[1] "2022-12-06 Mid 0.300 (95%CI 0.213 to 0.398) Estimated Error 0.0464"

[1] "2022-12-29 Mid 0.301 (95%CI 0.209 to 0.403) Estimated Error 0.0486"

24.7% Posterior Probability of decrease from 2017 to 2023

Klebsiella sp. ICU

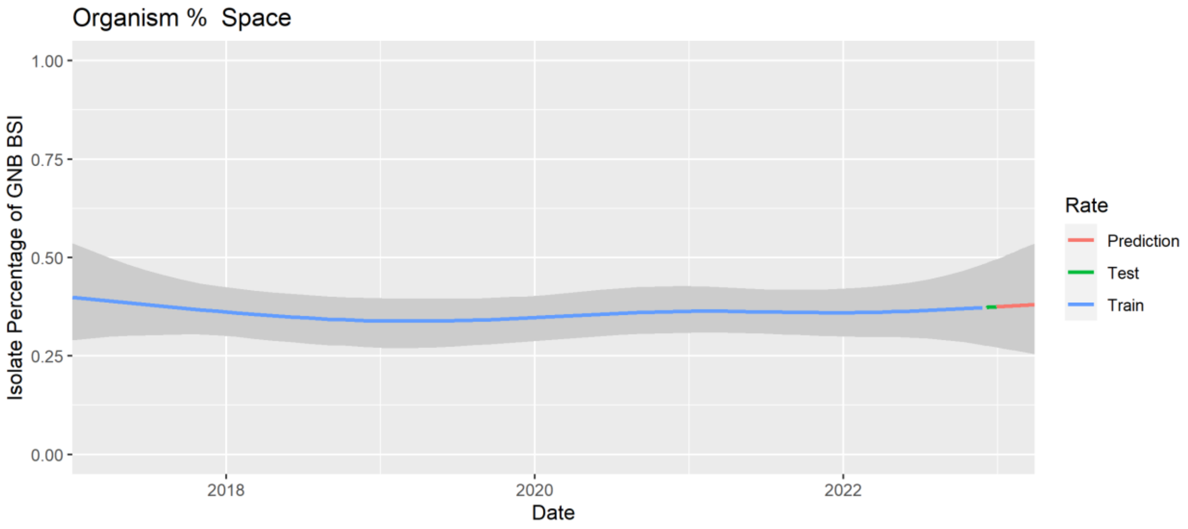

[1] "2017-01-01 Mid 0.399 (95%CI 0.291 to 0.537) Estimated Error 0.0637"

[1] "2022-12-06 Mid 0.373 (95%CI 0.276 to 0.486) Estimated Error 0.0525"

[1] "2022-12-29 Mid 0.375 (95%CI 0.272 to 0.495) Estimated Error 0.0556"

61.5% Posterior Probability of decrease from 2017 to 2023

SPACE ICU

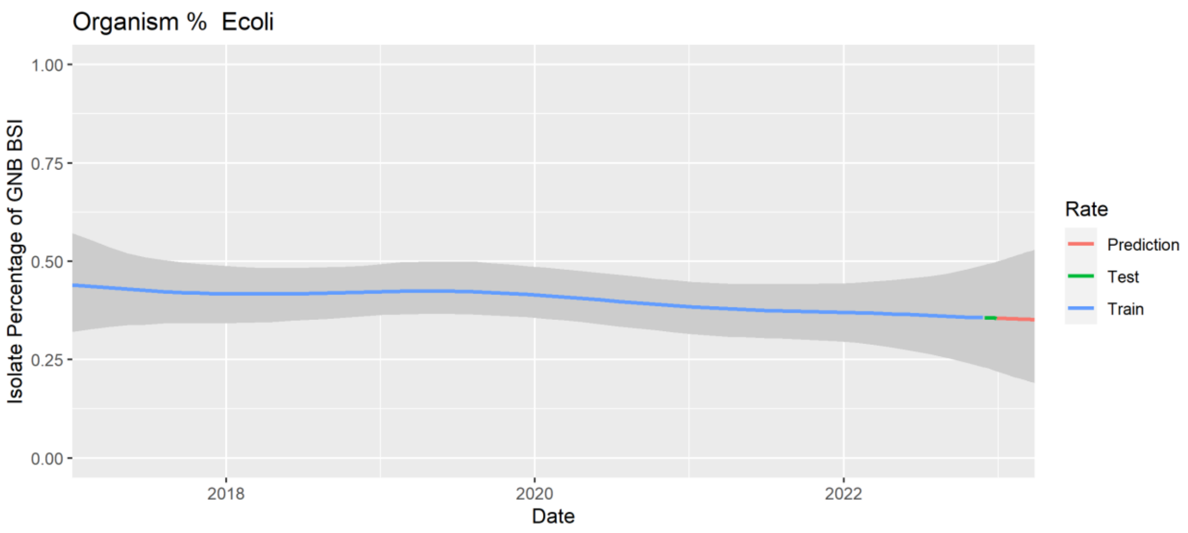

[1] "2017-01-02 Mid 0.440 (95%CI 0.321 to 0.572) Estimated Error 0.0630"

[1] "2022-11-30 Mid 0.356 (95%CI 0.230 to 0.491) Estimated Error 0.0659"

[1] "2022-12-28 Mid 0.355 (95%CI 0.220 to 0.499) Estimated Error 0.0698"

81.9% Posterior Probability of decrease from 2017 to 2023

E. coli Haemato-Oncology

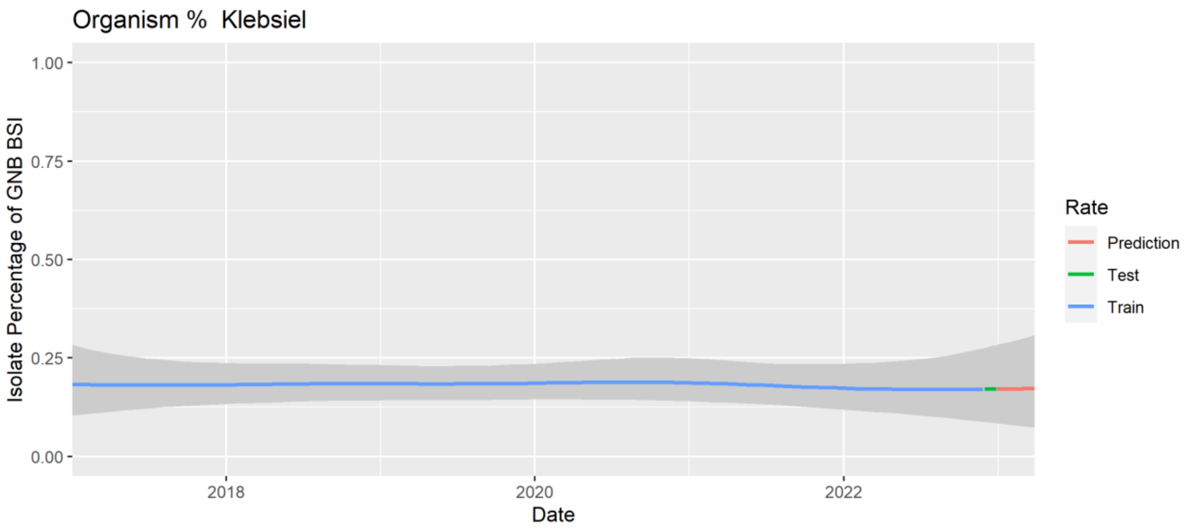

[1] "2017-01-02 Mid 0.183 (95%CI 0.104 to 0.283) Estimated Error 0.0453"

[1] "2022-11-30 Mid 0.170 (95%CI 0.087 to 0.275) Estimated Error 0.0473"

[1] "2022-12-28 Mid 0.171 (95%CI 0.084 to 0.282) Estimated Error 0.0499"

57.6% Posterior Probability of decrease from 2017 to 2023

Klebsiella sp. Haemato-Oncology

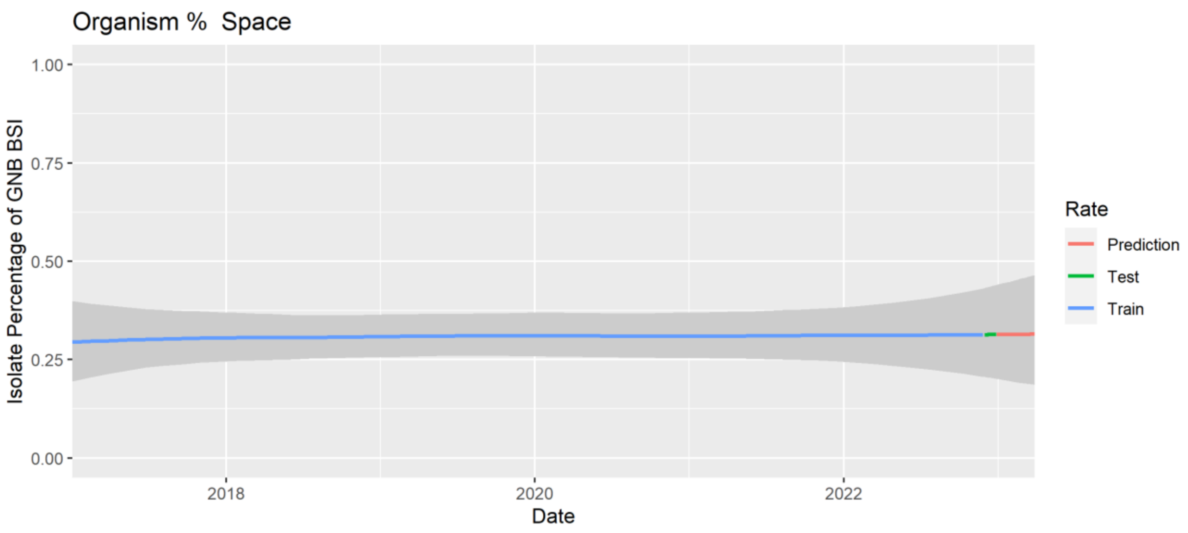

[1] "2017-01-02 Mid 0.295 (95%CI 0.194 to 0.399) Estimated Error 0.0519"

[1] "2022-11-30 Mid 0.313 (95%CI 0.206 to 0.432) Estimated Error 0.0573"

[1] "2022-12-28 Mid 0.314 (95%CI 0.201 to 0.439) Estimated Error 0.0602"

40.9% Posterior Probability of decrease from 2017 to 2023

SPACE organisms Haemato-Oncology

##

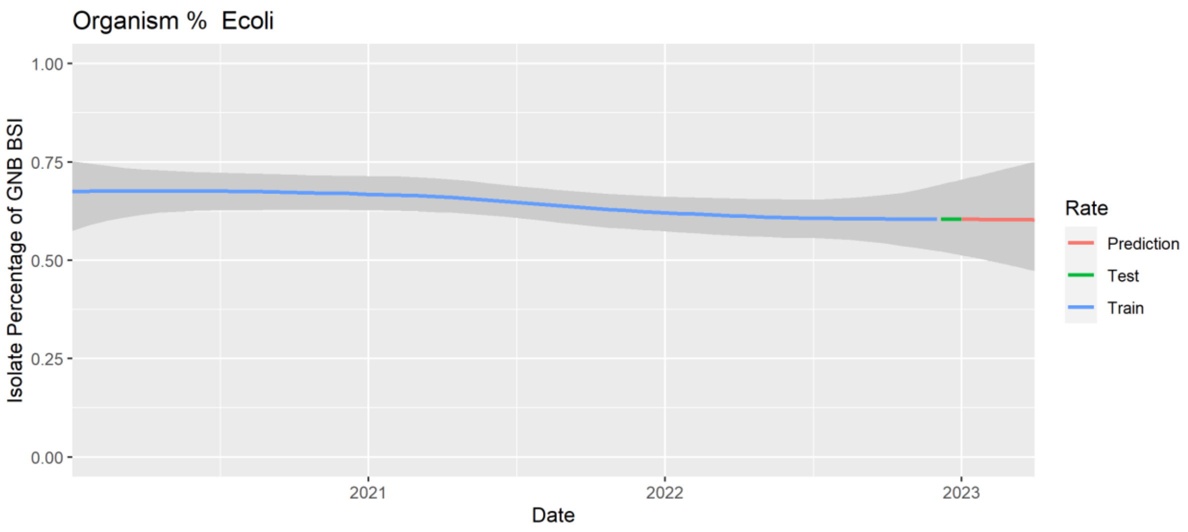

[1] "2020-01-02 Mid 0.675 (95%CI 0.575 to 0.751) Estimated Error 0.0452"

[1] "2022-12-06 Mid 0.604 (95%CI 0.522 to 0.693) Estimated Error 0.0426"

[1] "2022-12-31 Mid 0.604 (95%CI 0.512 to 0.705) Estimated Error 0.0478"

86.7% Posterior Probability of decrease from 2017 to 2023

GI source E. coli

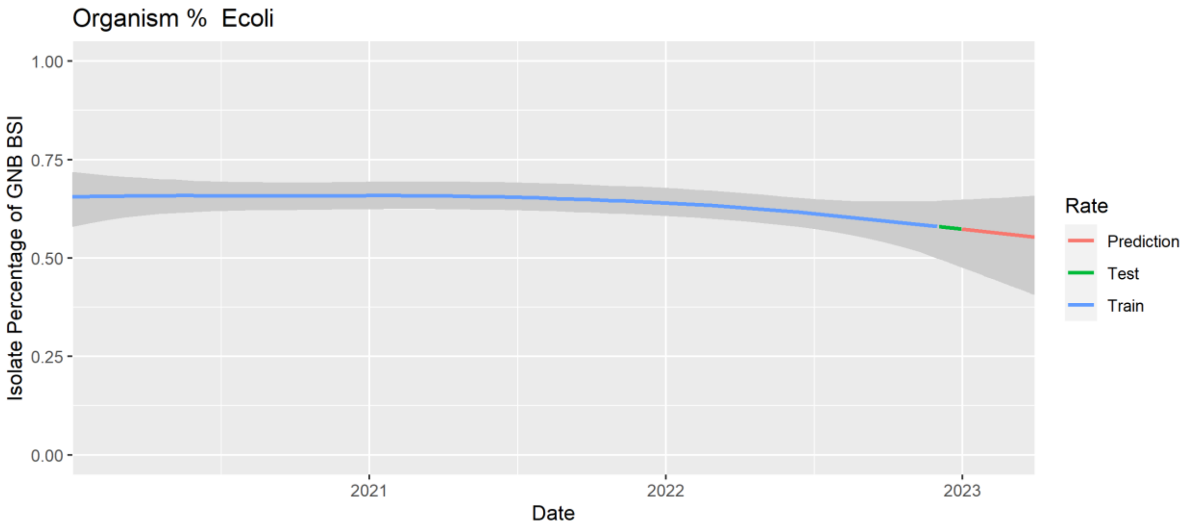

1] "2020-01-01 Mid 0.656 (95%CI 0.579 to 0.719) Estimated Error 0.0354"

[1] "2022-12-03 Mid 0.580 (95%CI 0.497 to 0.646) Estimated Error 0.0379"

[1] "2022-12-30 Mid 0.573 (95%CI 0.476 to 0.648) Estimated Error 0.0434"

"94.3% x greater than y

Urine source E. coli

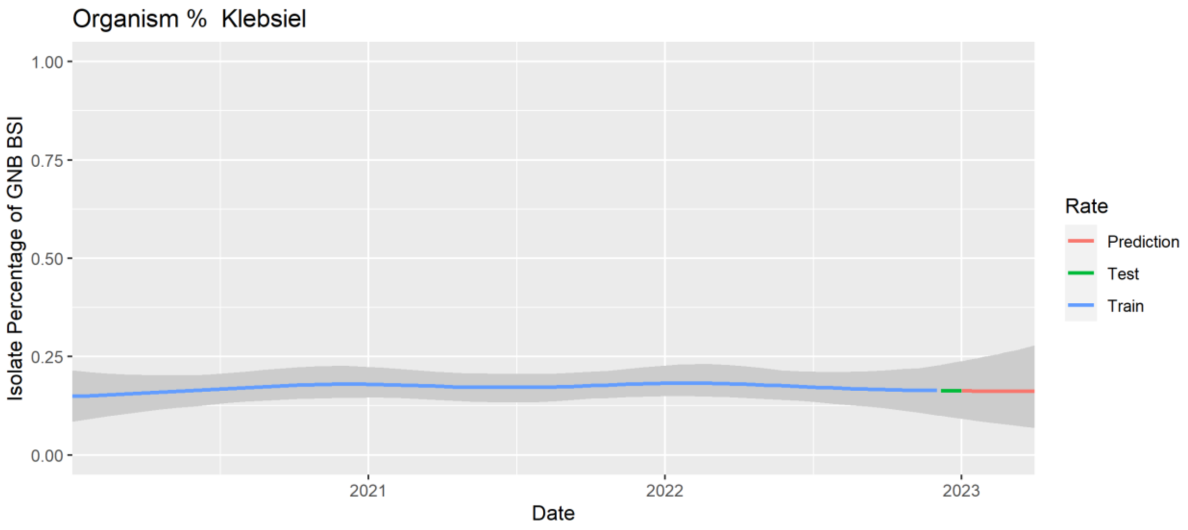

[1] "2020-01-02 Mid 0.149 (95%CI 0.085 to 0.215) Estimated Error 0.0335"

[1] "2022-12-06 Mid 0.163 (95%CI 0.099 to 0.229) Estimated Error 0.0327"

[1] "2022-12-31 Mid 0.163 (95%CI 0.092 to 0.238) Estimated Error 0.0365"

37.2% Posterior Probability of decrease from 2017 to 2023

GI Klebsiella sp.

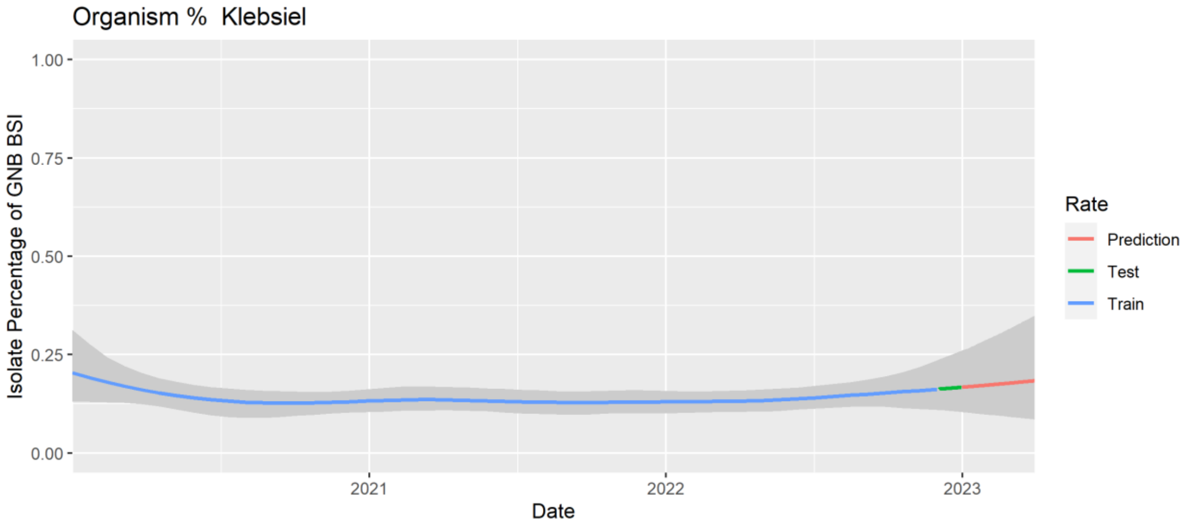

[1] "2020-01-01 Mid 0.204 (95%CI 0.130 to 0.313) Estimated Error 0.0486"

[1] "2022-12-03 Mid 0.162 (95%CI 0.109 to 0.238) Estimated Error 0.0325"

[1] "2022-12-30 Mid 0.167 (95%CI 0.104 to 0.260) Estimated Error 0.0395"

77.6% Posterior Probability of decrease from 2017 to 2023

Urine Source Klebsiella sp.

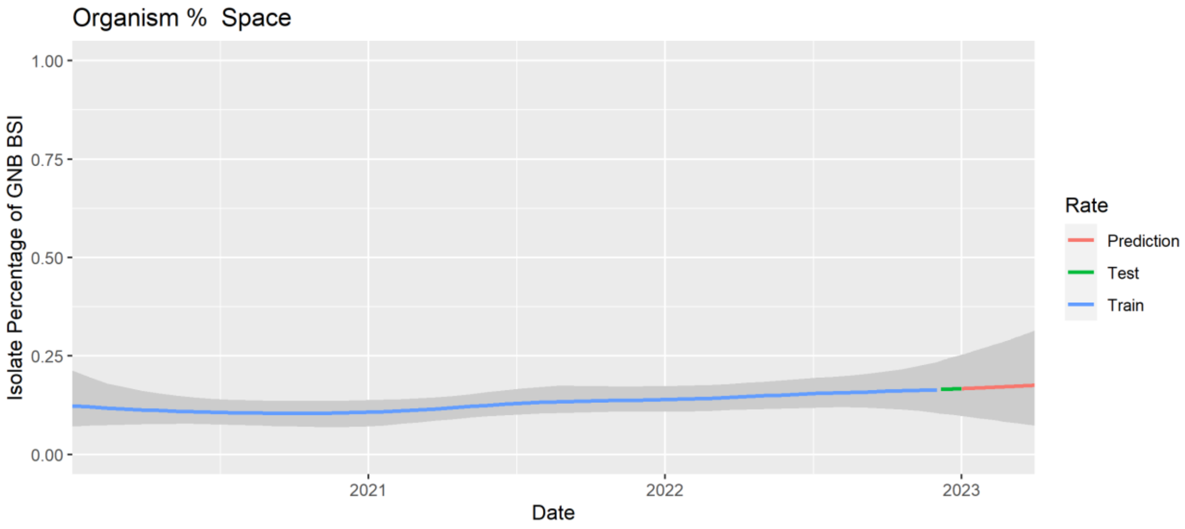

[1] "2020-01-02 Mid 0.124 (95%CI 0.071 to 0.213) Estimated Error 0.0362"

[1] "2022-12-06 Mid 0.165 (95%CI 0.103 to 0.238) Estimated Error 0.0338"

[1] "2022-12-31 Mid 0.167 (95%CI 0.097 to 0.253) Estimated Error 0.0388"

19.3% Posterior Probability of decrease from 2017 to 2023

GI Source SPACE Organisms

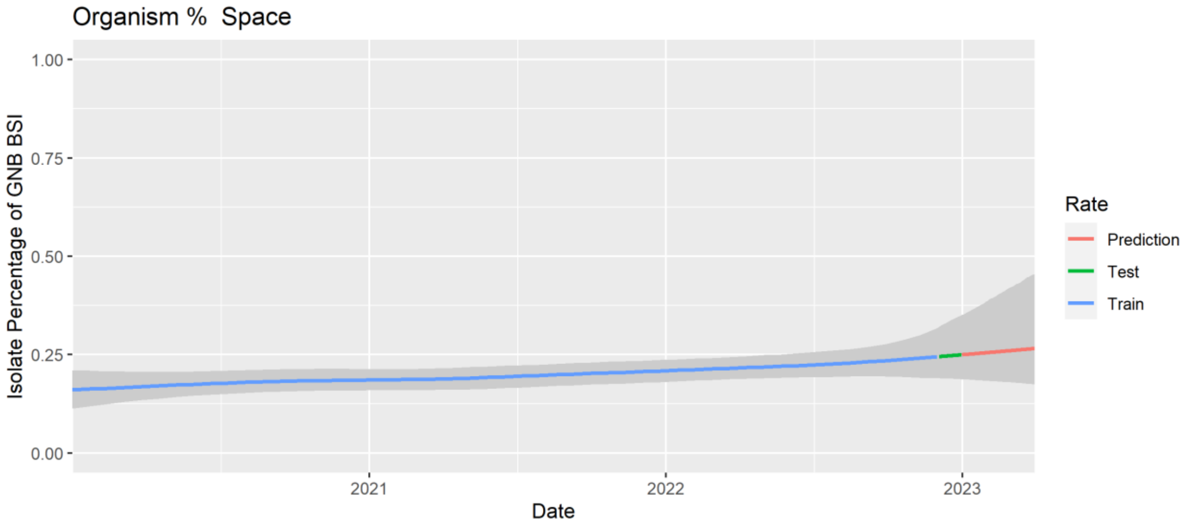

[1] "2020-01-01 Mid 0.161 (95%CI 0.113 to 0.211) Estimated Error 0.0245"

[1] "2022-12-03 Mid 0.245 (95%CI 0.190 to 0.321) Estimated Error 0.0322"

[1] "2022-12-30 Mid 0.249 (95%CI 0.187 to 0.350) Estimated Error 0.0385"

1.6% Posterior Probability of decrease from 2017 to 2023

Urine Source SPACE Organisms

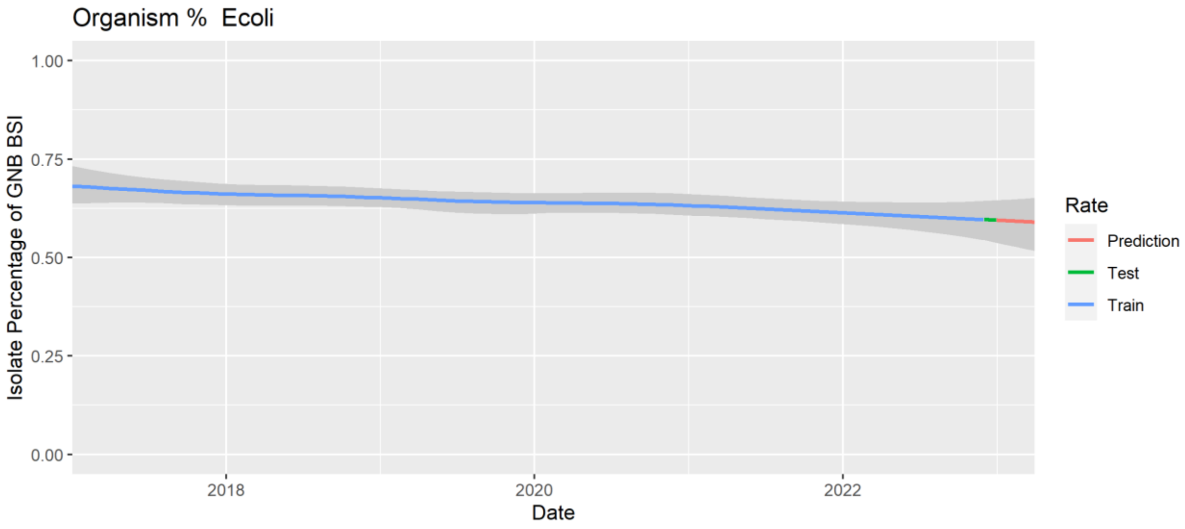

[1] "2017-01-01 Mid 0.682 (95%CI 0.637 to 0.732) Estimated Error 0.0236"

[1] "2022-12-01 Mid 0.596 (95%CI 0.544 to 0.644) Estimated Error 0.0254"

[1] "2022-12-30 Mid 0.595 (95%CI 0.537 to 0.645) Estimated Error 0.0272"

99.4% Posterior Probability of decrease from 2017 to 2023

Patients Over 80 years E. coli

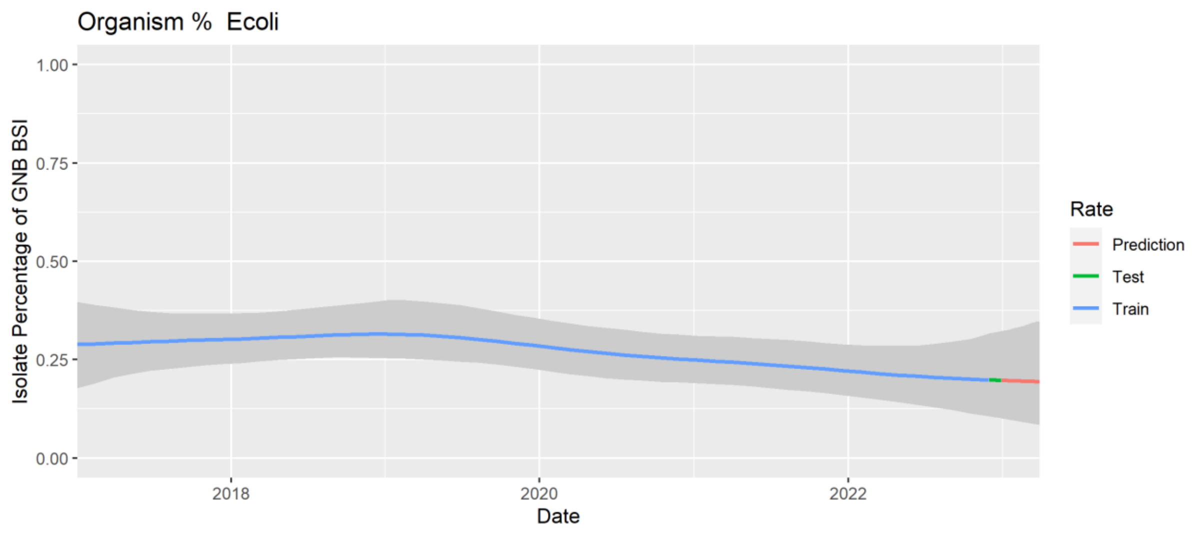

[1] "2017-01-02 Mid 0.289 (95%CI 0.178 to 0.396) Estimated Error 0.0560"

[1] "2022-11-30 Mid 0.198 (95%CI 0.105 to 0.316) Estimated Error 0.0525"

[1] "2022-12-29 Mid 0.197 (95%CI 0.101 to 0.322) Estimated Error 0.0558"

88.5% x greater than y

Patients Under 18 years E. coli

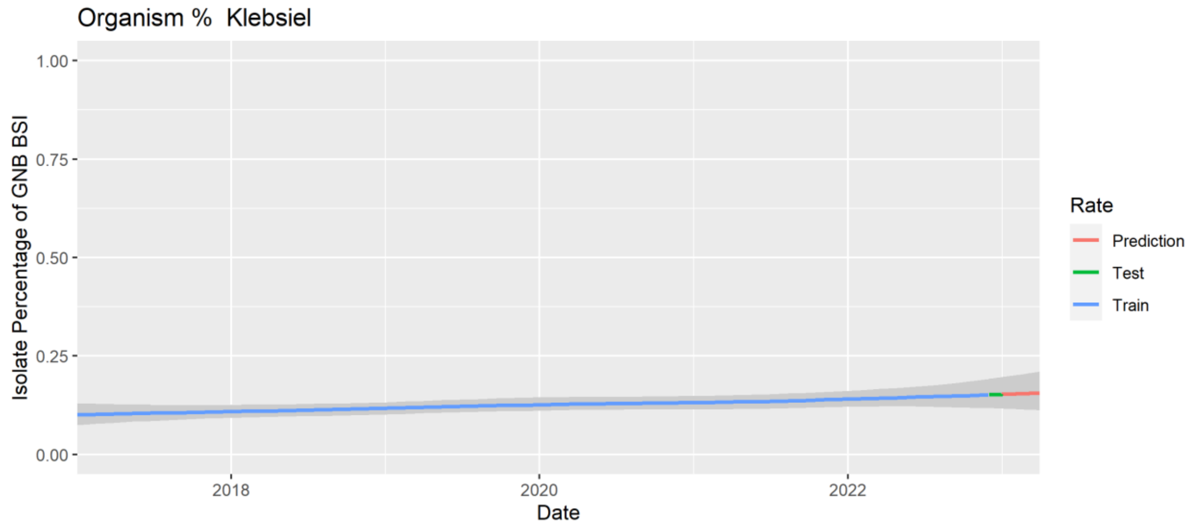

[1] "2017-01-01 Mid 0.101 (95%CI 0.075 to 0.130) Estimated Error 0.0139"

[1] "2022-12-01 Mid 0.151 (95%CI 0.117 to 0.192) Estimated Error 0.0185"

[1] "2022-12-30 Mid 0.152 (95%CI 0.116 to 0.196) Estimated Error 0.0198"

1.5% Posterior Probability of decrease from 2017 to 2023

Patients Over 80 years Klebsiella sp.

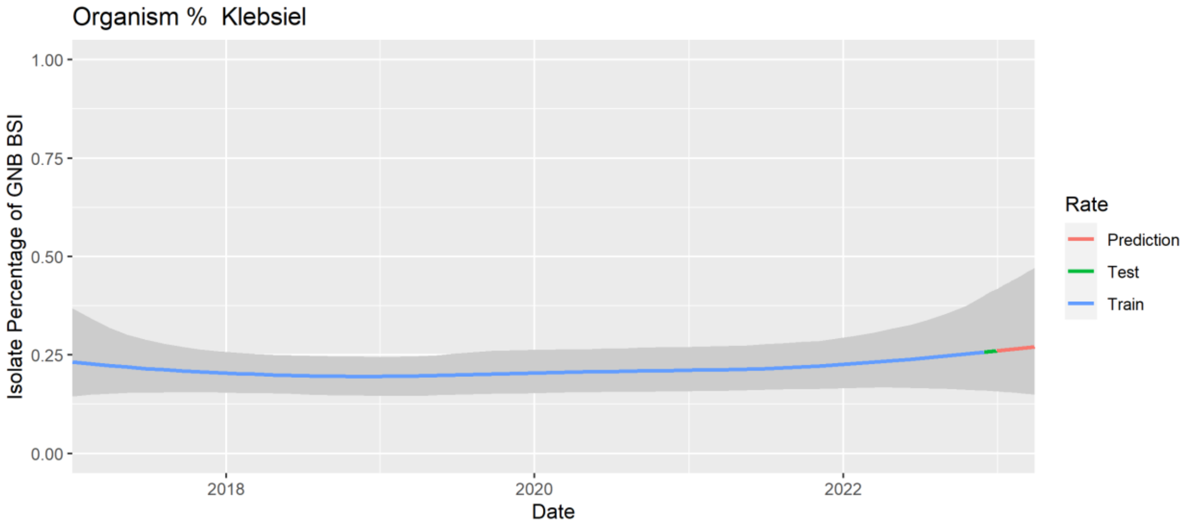

[1] "2017-01-02 Mid 0.232 (95%CI 0.145 to 0.369) Estimated Error 0.0561"

[1] "2022-11-30 Mid 0.257 (95%CI 0.159 to 0.400) Estimated Error 0.0606"

[1] "2022-12-29 Mid 0.260 (95%CI 0.157 to 0.416) Estimated Error 0.0652"

37.0% Posterior Probability of decrease from 2017 to 2023

Patients Under 18 years   Klebsiella sp.

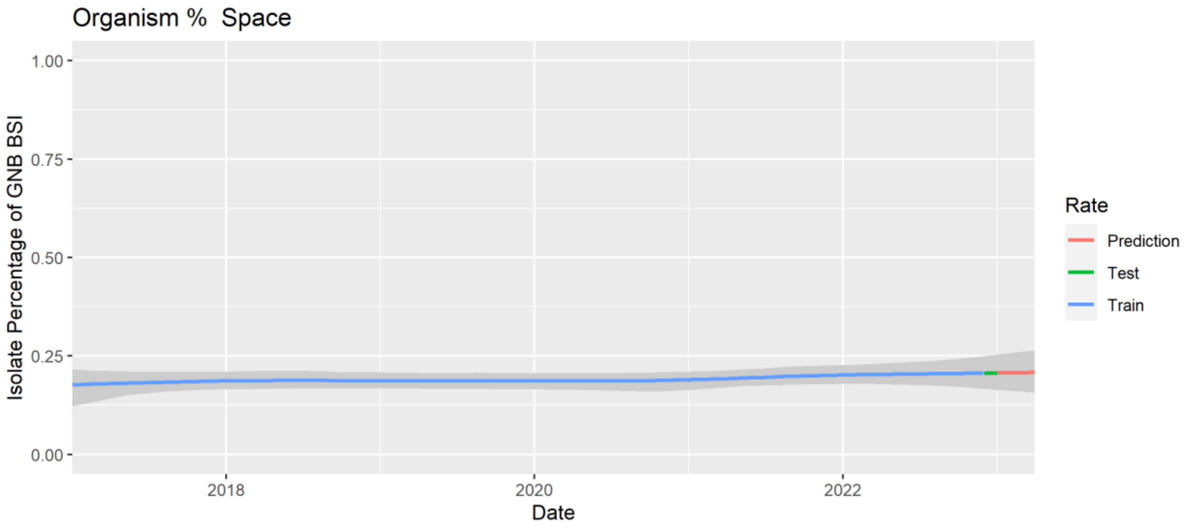

[1] "2017-01-01 Mid 0.177 (95%CI 0.123 to 0.217) Estimated Error 0.0217"

[1] "2022-12-01 Mid 0.206 (95%CI 0.167 to 0.250) Estimated Error 0.0207"

[1] "2022-12-30 Mid 0.207 (95%CI 0.164 to 0.252) Estimated Error 0.0221"

16.3% Posterior Probability of decrease from 2017 to 2023

Patients Over 80 years SPACE Organisms

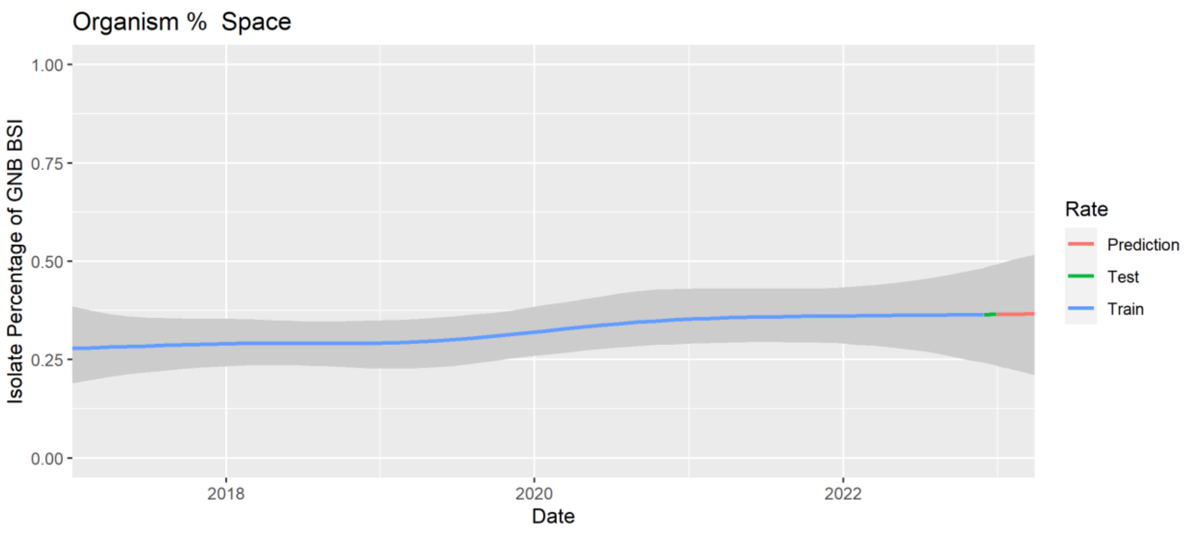

[1] "2017-01-02 Mid 0.279 (95%CI 0.191 to 0.385) Estimated Error 0.0492"

[1] "2022-11-30 Mid 0.364 (95%CI 0.242 to 0.484) Estimated Error 0.0618"

[1] "2022-12-29 Mid 0.365 (95%CI 0.235 to 0.492) Estimated Error 0.0654"

14.4% Posterior Probability of decrease from 2017 to 2023

Patients Under 18 years   SPACE Organisms

Supplementary Material (AMR Percentages)

Antibiotic resistance over time, with the probability of decreasing resistance between the start and end points (All patients, for E. coli and Non-E. coli isolates)

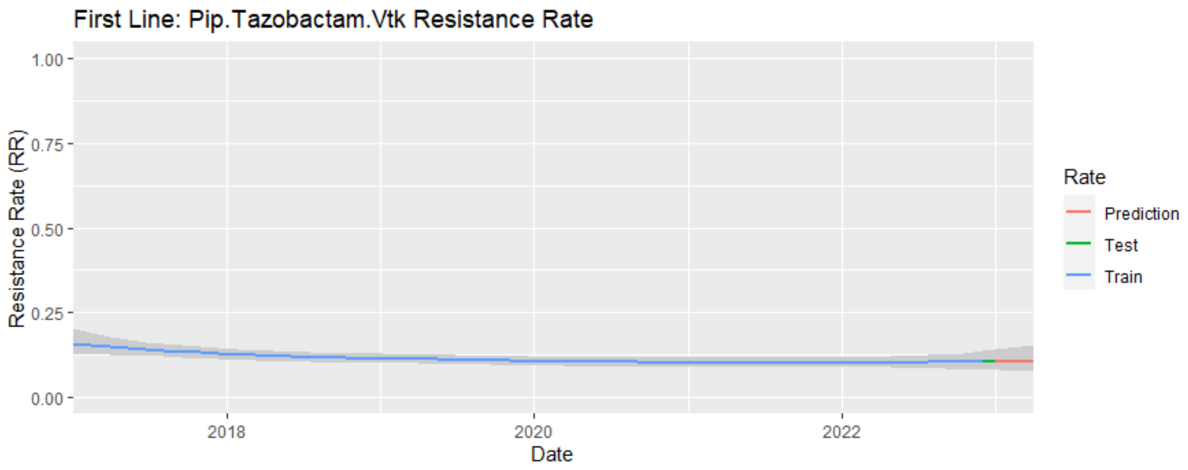

[1] "2017-01-01 Mid 0.157 (95%CI 0.125 to 0.200) Estimated Error 0.0196"

[1] "2022-12-03 Mid 0.104 (95%CI 0.080 to 0.135) Estimated Error 0.0142"

[1] "2022-12-31 Mid 0.104 (95%CI 0.078 to 0.139) Estimated Error 0.0154"

"99.4% posterior probability of decrease "

E. coli

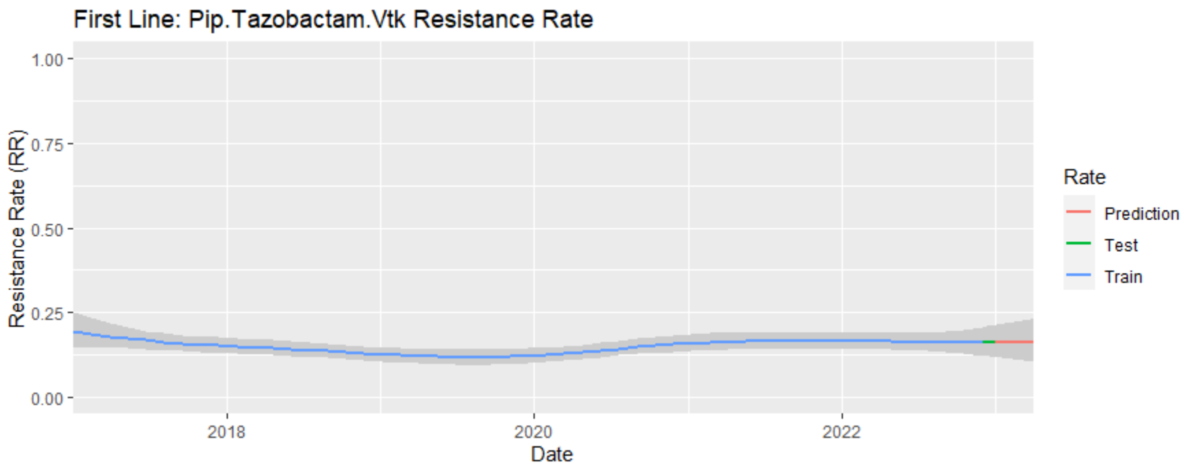

[1] "2017-01-01 Mid 0.193 (95%CI 0.143 to 0.251) Estimated Error 0.0271"

[1] "2022-12-02 Mid 0.162 (95%CI 0.121 to 0.206) Estimated Error 0.0218"

[1] "2022-12-31 Mid 0.162 (95%CI 0.117 to 0.211) Estimated Error 0.0241"

"80.4% posterior probability of decrease "

Non-Ecoli

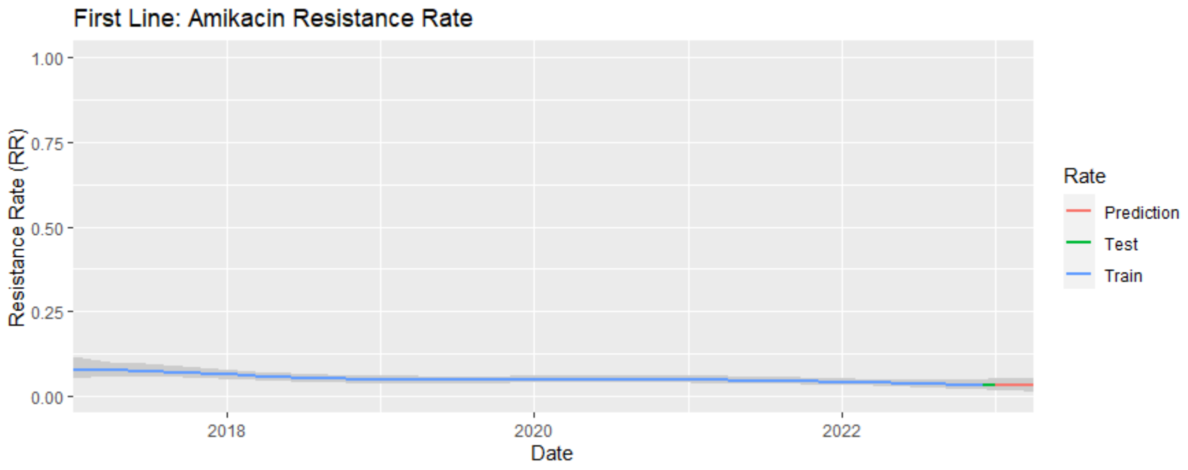

[1] "2017-01-02 Mid 0.079 (95%CI 0.052 to 0.115) Estimated Error 0.0160"

[1] "2022-12-03 Mid 0.032 (95%CI 0.017 to 0.049) Estimated Error 0.0081"

[1] "2022-12-31 Mid 0.031 (95%CI 0.016 to 0.050) Estimated Error 0.0086"

"99.9% posterior probability of decrease "

E. coli

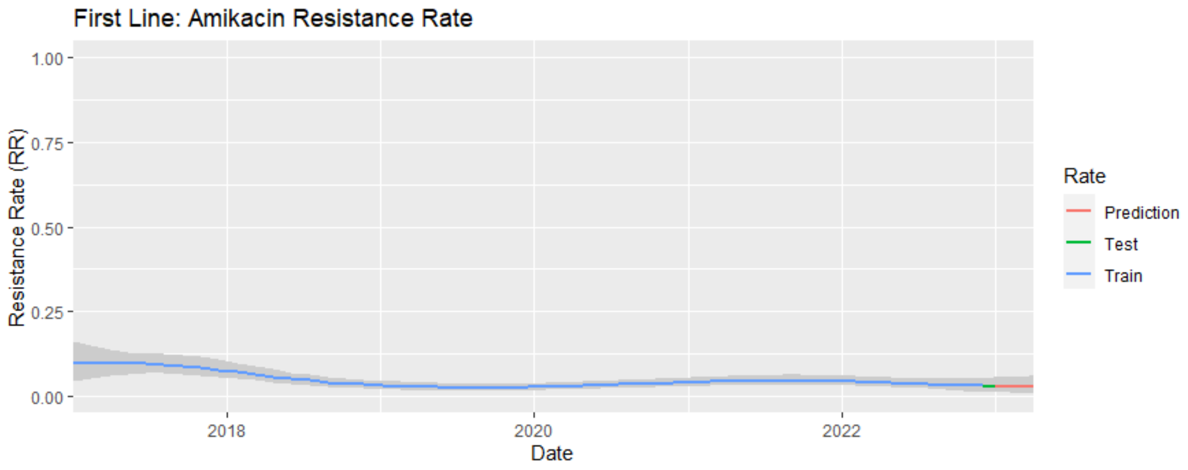

[1] "2017-01-01 Mid 0.096 (95%CI 0.045 to 0.160) Estimated Error 0.0294"

[1] "2022-12-03 Mid 0.029 (95%CI 0.012 to 0.052) Estimated Error 0.0103"

[1] "2022-12-31 Mid 0.028 (95%CI 0.011 to 0.053) Estimated Error 0.0111"

"99.3% posterior probability of decrease "

Non-Ecoli

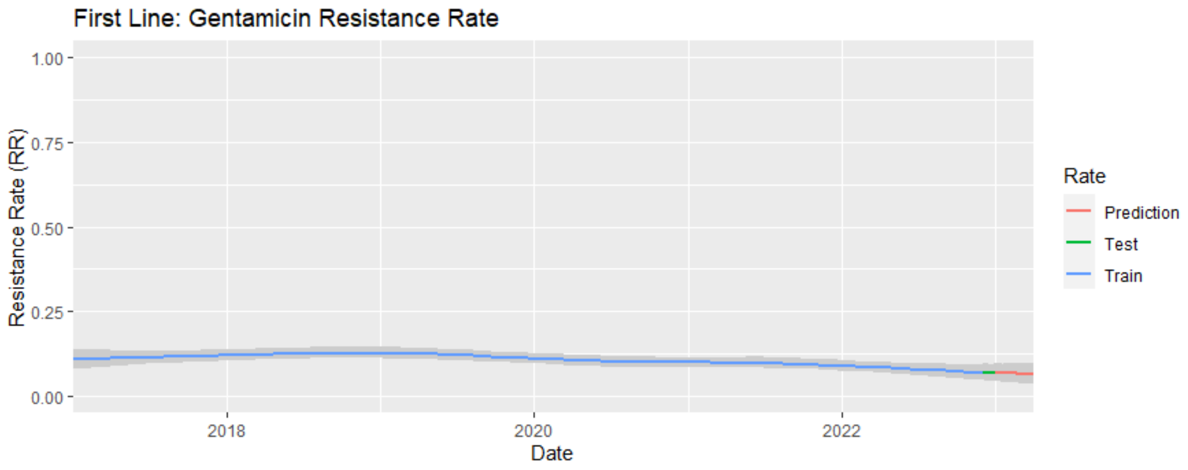

[1] "2017-01-01 Mid 0.107 (95%CI 0.079 to 0.138) Estimated Error 0.0156"

[1] "2022-12-03 Mid 0.069 (95%CI 0.046 to 0.094) Estimated Error 0.0123"

[1] "2022-12-31 Mid 0.068 (95%CI 0.043 to 0.094) Estimated Error 0.0131"

"97.9% posterior probability of decrease "

E. coli

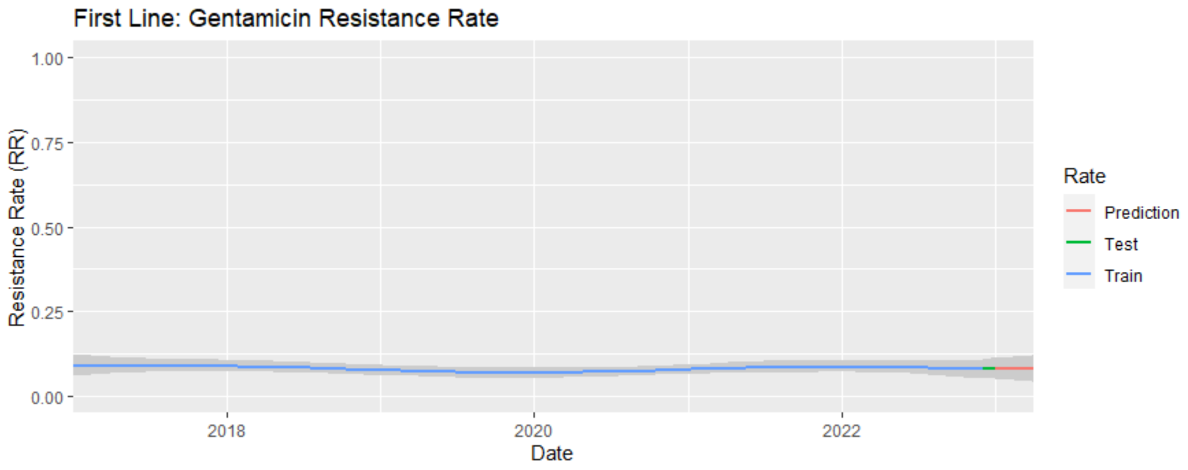

[1] "2017-01-01 Mid 0.090 (95%CI 0.060 to 0.126) Estimated Error 0.0164"

[1] "2022-12-02 Mid 0.080 (95%CI 0.052 to 0.109) Estimated Error 0.0143"

[1] "2022-12-31 Mid 0.079 (95%CI 0.050 to 0.111) Estimated Error 0.0155"

"69.5% posterior probability of decrease "

Non-Ecoli

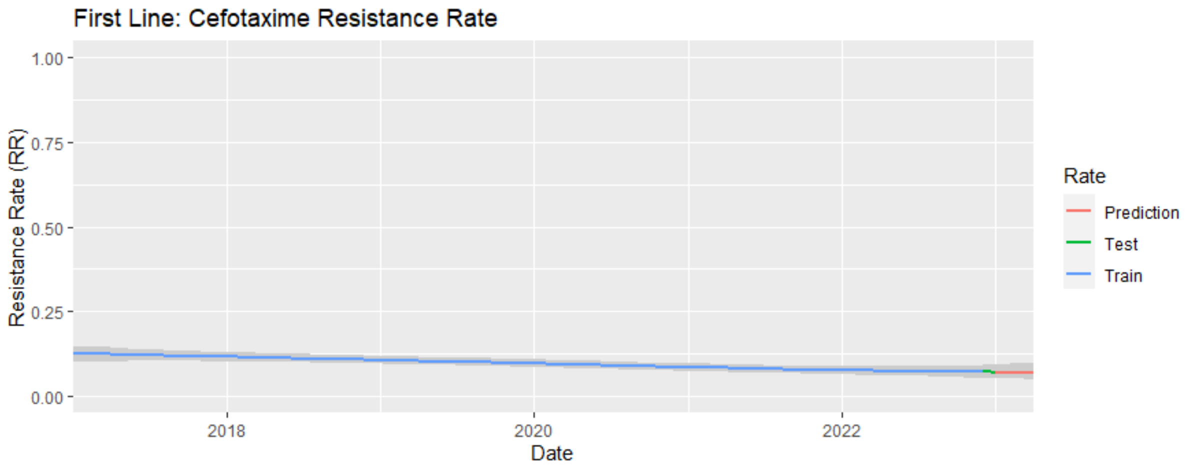

[1] "2017-01-01 Mid 0.125 (95%CI 0.099 to 0.151) Estimated Error 0.0130"

[1] "2022-12-03 Mid 0.070 (95%CI 0.053 to 0.091) Estimated Error 0.0095"

[1] "2022-12-31 Mid 0.070 (95%CI 0.052 to 0.092) Estimated Error 0.0100"

"99.9% posterior probability of decrease "

E. coli

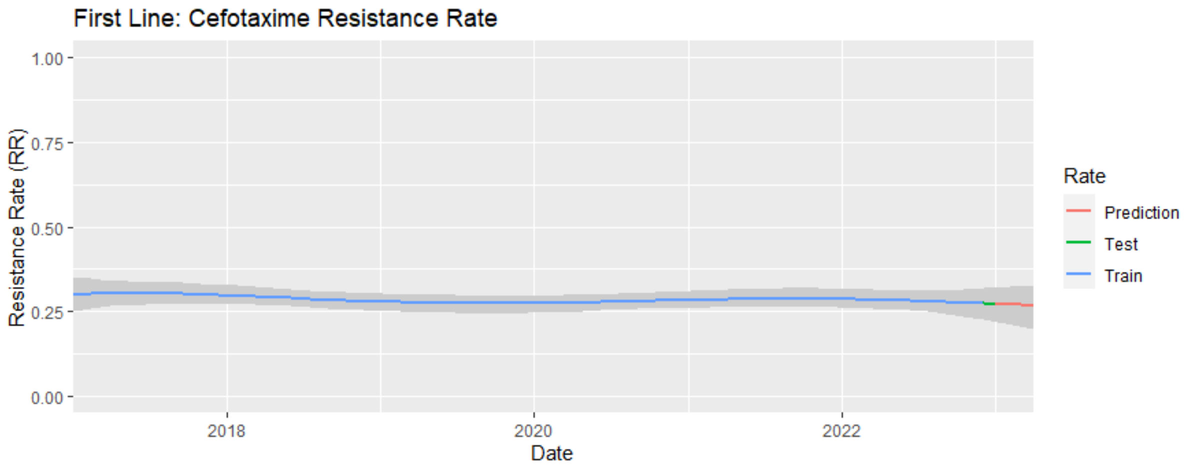

[1] "2017-01-01 Mid 0.301 (95%CI 0.249 to 0.352) Estimated Error 0.0257"

[1] "2022-12-02 Mid 0.273 (95%CI 0.225 to 0.317) Estimated Error 0.0231"

[1] "2022-12-31 Mid 0.272 (95%CI 0.218 to 0.319) Estimated Error 0.0252"

"80.2% posterior probability of decrease "

Non-Ecoli

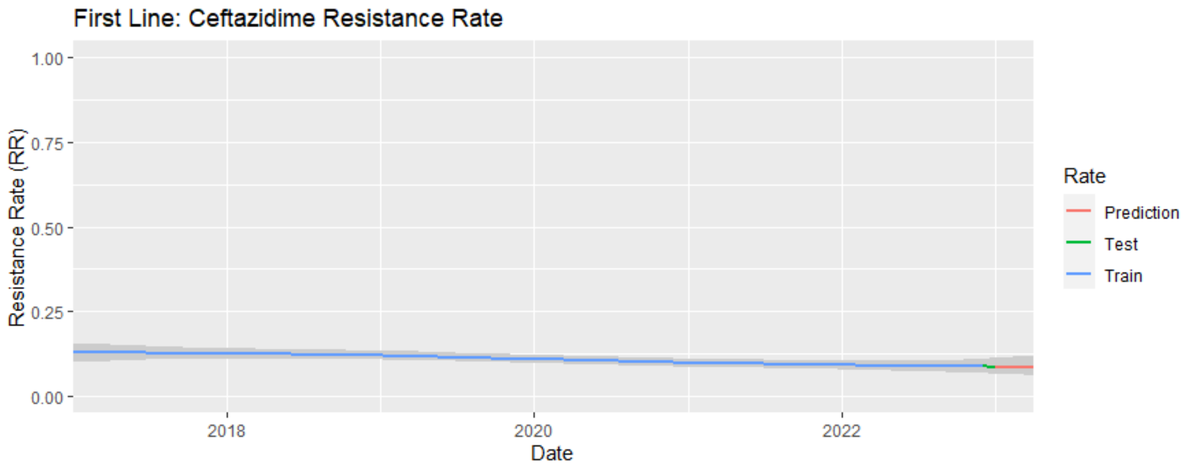

[1] "2017-01-01 Mid 0.129 (95%CI 0.099 to 0.158) Estimated Error 0.0147"

[1] "2022-12-03 Mid 0.086 (95%CI 0.066 to 0.109) Estimated Error 0.0108"

[1] "2022-12-31 Mid 0.086 (95%CI 0.065 to 0.111) Estimated Error 0.0115"

"98.5% posterior probability of decrease "

E. coli

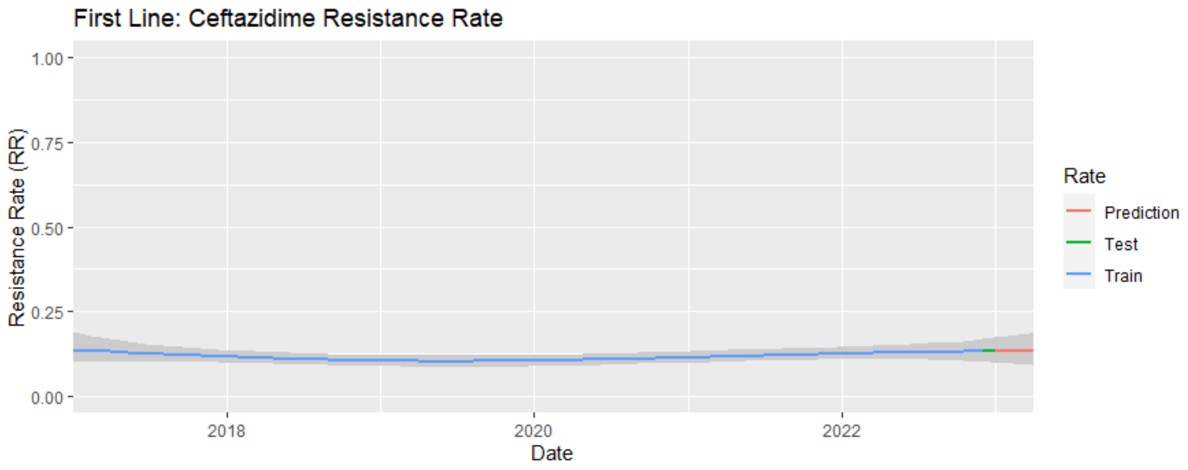

[1] "2017-01-01 Mid 0.137 (95%CI 0.100 to 0.188) Estimated Error 0.0228"

[1] "2022-12-02 Mid 0.132 (95%CI 0.100 to 0.168) Estimated Error 0.0174"

[1] "2022-12-31 Mid 0.133 (95%CI 0.098 to 0.172) Estimated Error 0.0189"

"54.2% posterior probability of decrease "

Non-Ecoli

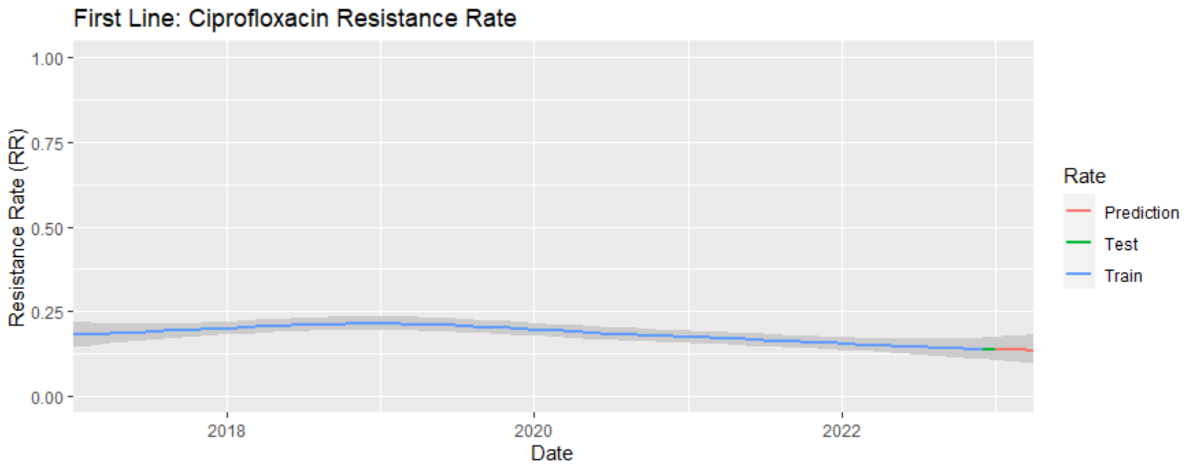

[1] "2017-01-01 Mid 0.180 (95%CI 0.143 to 0.218) Estimated Error 0.0192"

[1] "2022-12-03 Mid 0.139 (95%CI 0.108 to 0.172) Estimated Error 0.0163"

[1] "2022-12-31 Mid 0.137 (95%CI 0.105 to 0.174) Estimated Error 0.0175"

"95.0% posterior probability of decrease "

E. coli

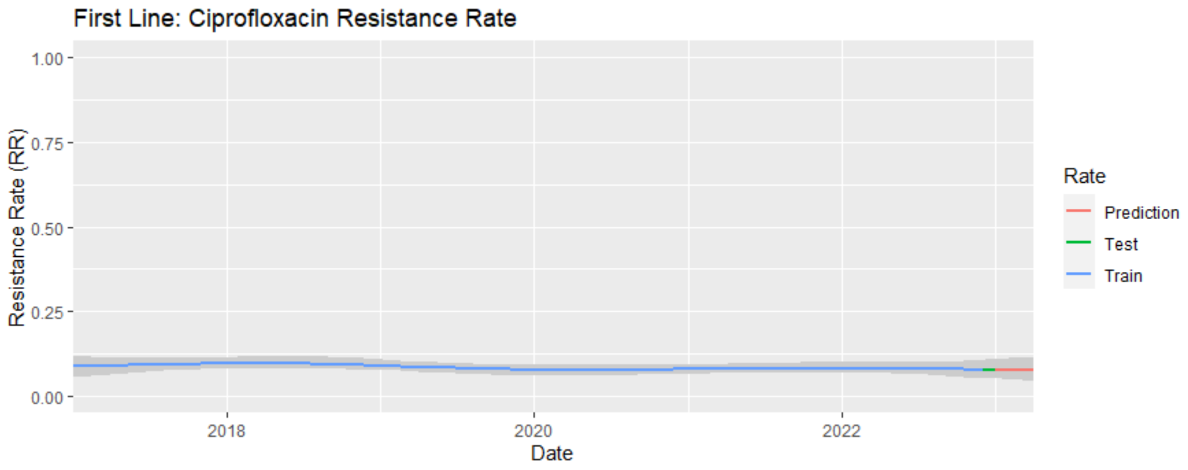

[1] "2017-01-01 Mid 0.088 (95%CI 0.055 to 0.118) Estimated Error 0.0159"

[1] "2022-12-02 Mid 0.078 (95%CI 0.053 to 0.106) Estimated Error 0.0129"

[1] "2022-12-31 Mid 0.077 (95%CI 0.051 to 0.108) Estimated Error 0.0140"

"70.5% posterior probability of decrease "

Non-Ecoli

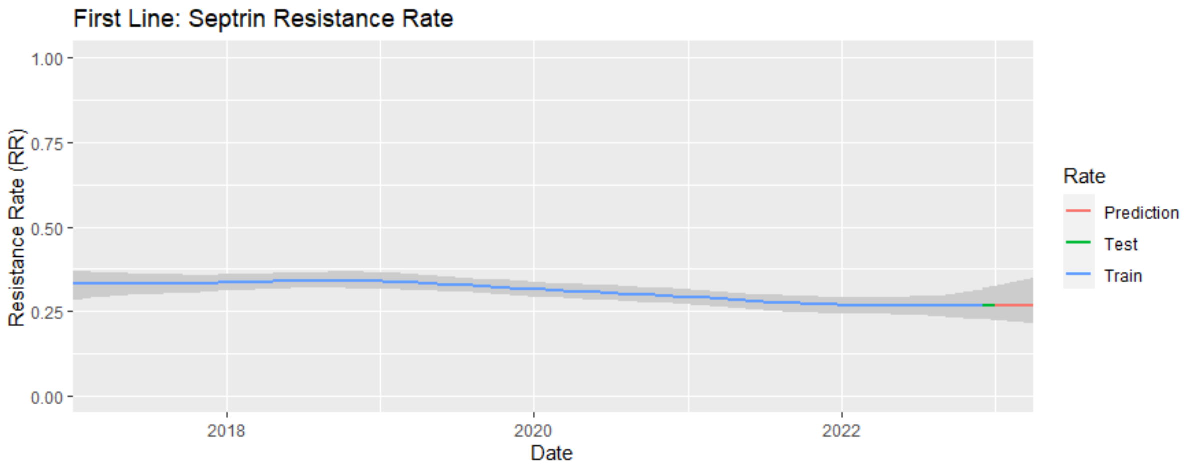

[1] "2017-01-01 Mid 0.330 (95%CI 0.283 to 0.373) Estimated Error 0.0229"

[1] "2022-12-03 Mid 0.267 (95%CI 0.226 to 0.318) Estimated Error 0.0227"

[1] "2022-12-31 Mid 0.268 (95%CI 0.223 to 0.324) Estimated Error 0.0249"

"96.0% posterior probability of decrease "

E. coli

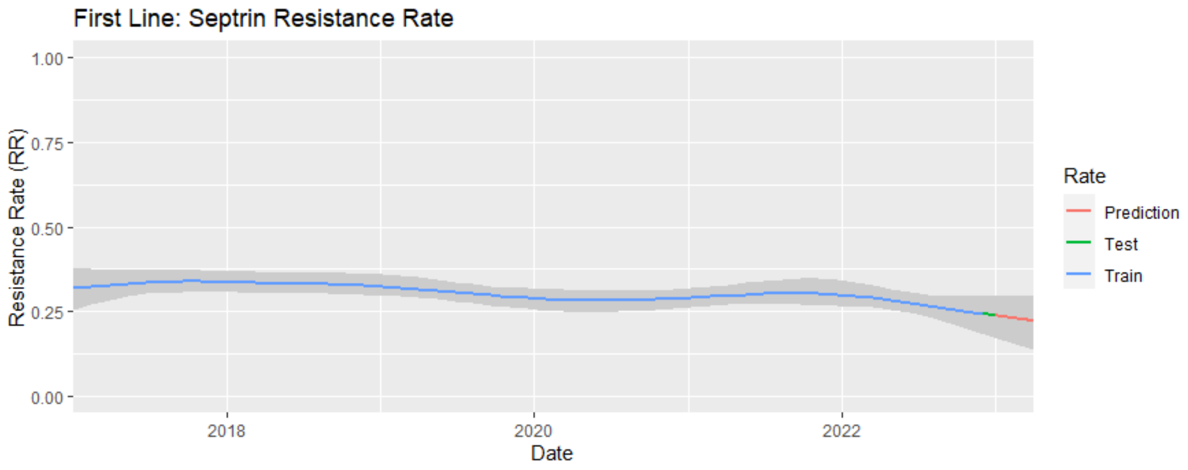

[1] "2017-01-01 Mid 0.321 (95%CI 0.254 to 0.380) Estimated Error 0.0323"

[1] "2022-12-02 Mid 0.242 (95%CI 0.182 to 0.295) Estimated Error 0.0297"

[1] "2022-12-31 Mid 0.237 (95%CI 0.170 to 0.294) Estimated Error 0.0331"

"98.4% posterior probability of decrease "
Non-Ecoli

Supplementary Material

Patients Over 80 or Under 18

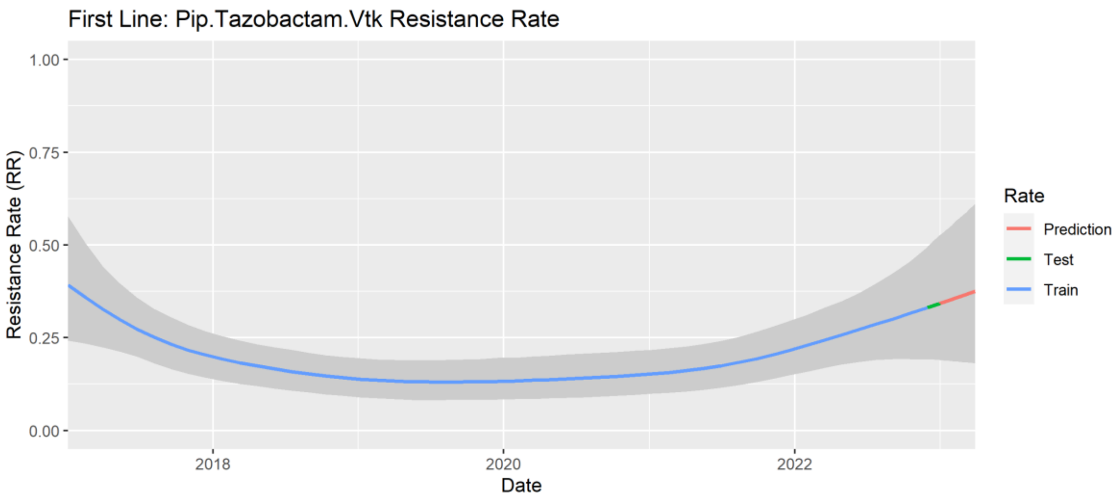

[1] "2017-01-02 Mid 0.392 (95%CI 0.243 to 0.577) Estimated Error 0.0853"

[1] "2022-11-30 Mid 0.331 (95%CI 0.192 to 0.495) Estimated Error 0.0786"

[1] "2022-12-29 Mid 0.342 (95%CI 0.190 to 0.524) Estimated Error 0.0862"

"70.6% posterior probability of decrease "

Under 18

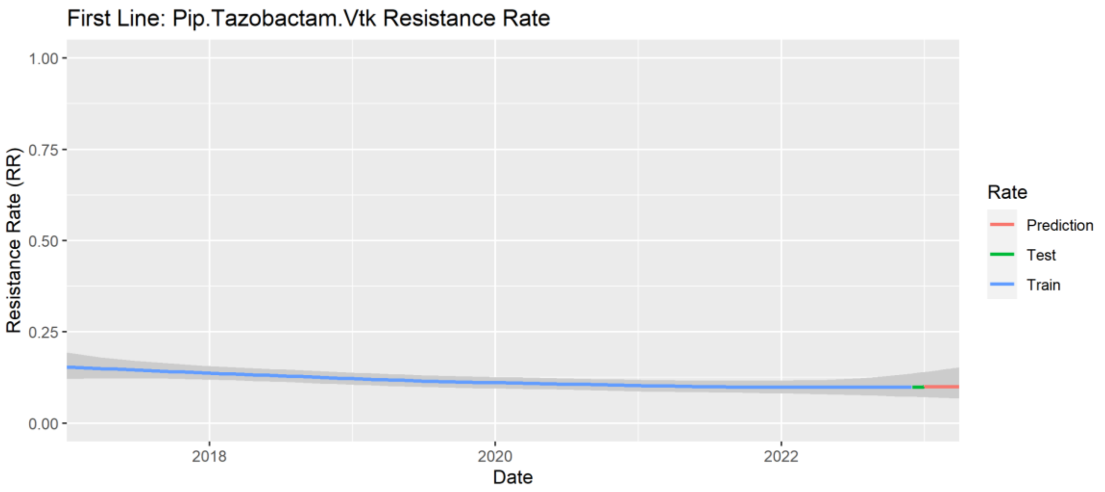

[1] "2017-01-01 Mid 0.154 (95%CI 0.121 to 0.194) Estimated Error 0.0183"

[1] "2022-12-01 Mid 0.099 (95%CI 0.072 to 0.136) Estimated Error 0.0162"

[1] "2022-12-30 Mid 0.099 (95%CI 0.071 to 0.139) Estimated Error 0.0174"

"98.5% posterior probability of decrease "

Over 80

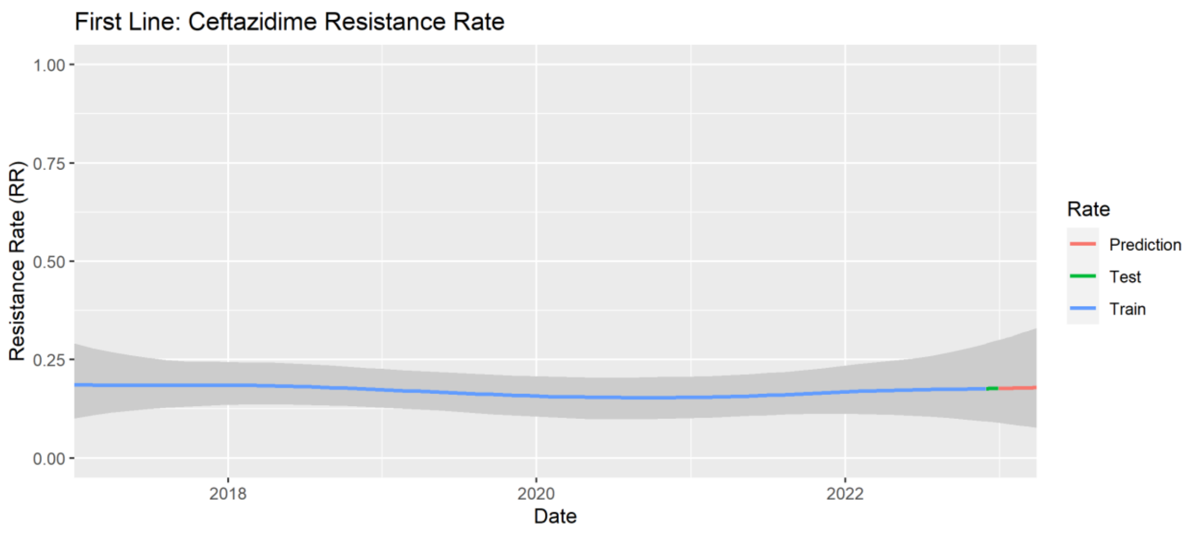

[1] "2017-01-02 Mid 0.186 (95%CI 0.101 to 0.291) Estimated Error 0.0476"

[1] "2022-11-30 Mid 0.176 (95%CI 0.093 to 0.291) Estimated Error 0.0500"

[1] "2022-12-29 Mid 0.176 (95%CI 0.089 to 0.299) Estimated Error 0.0530"

"56.6% posterior probability of decrease "

Under 18

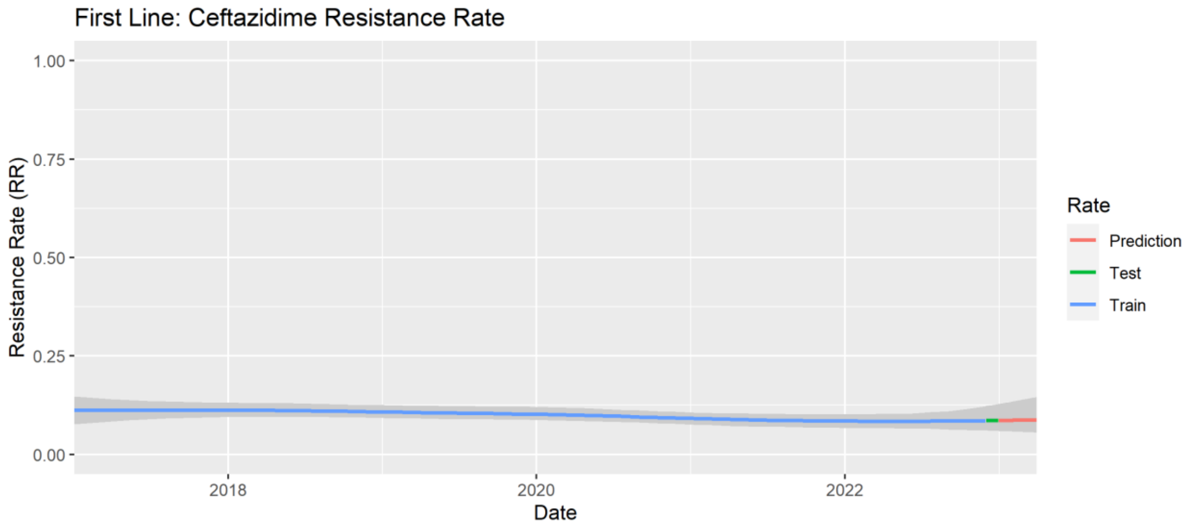

[1] "2017-01-01 Mid 0.112 (95%CI 0.076 to 0.147) Estimated Error 0.0180"

[1] "2022-12-01 Mid 0.086 (95%CI 0.060 to 0.123) Estimated Error 0.0155"

[1] "2022-12-30 Mid 0.086 (95%CI 0.059 to 0.128) Estimated Error 0.0170"

"86.1% posterior probability of decrease "

Over 80

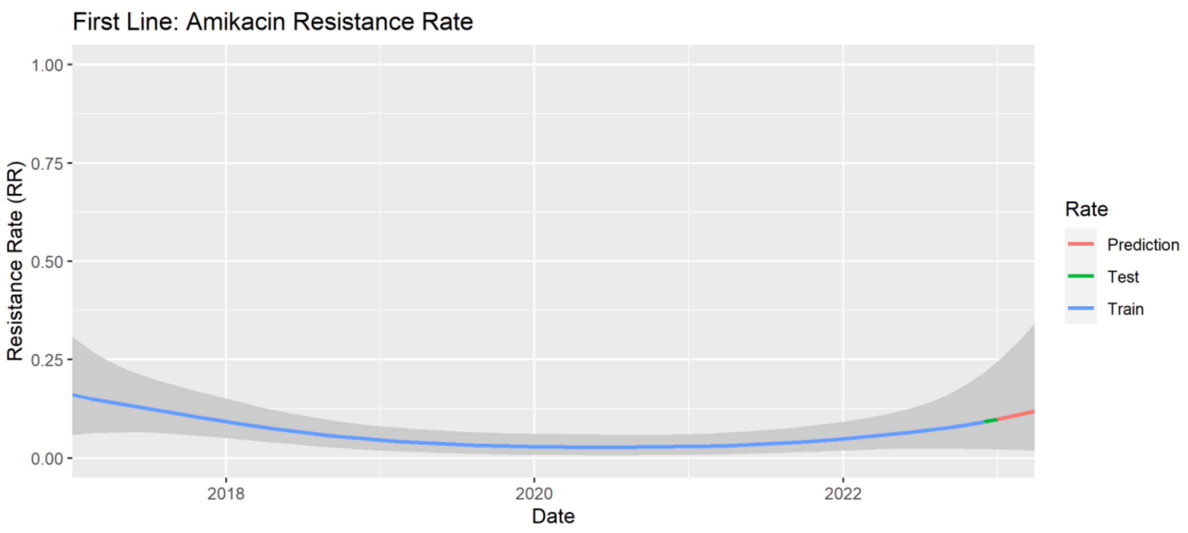

[1] "2017-01-02 Mid 0.160 (95%CI 0.059 to 0.308) Estimated Error 0.0647"

[1] "2022-11-30 Mid 0.092 (95%CI 0.022 to 0.217) Estimated Error 0.0515"

[1] "2022-12-29 Mid 0.098 (95%CI 0.021 to 0.242) Estimated Error 0.0585"

"81.7% posterior probability of decrease "

Under 18

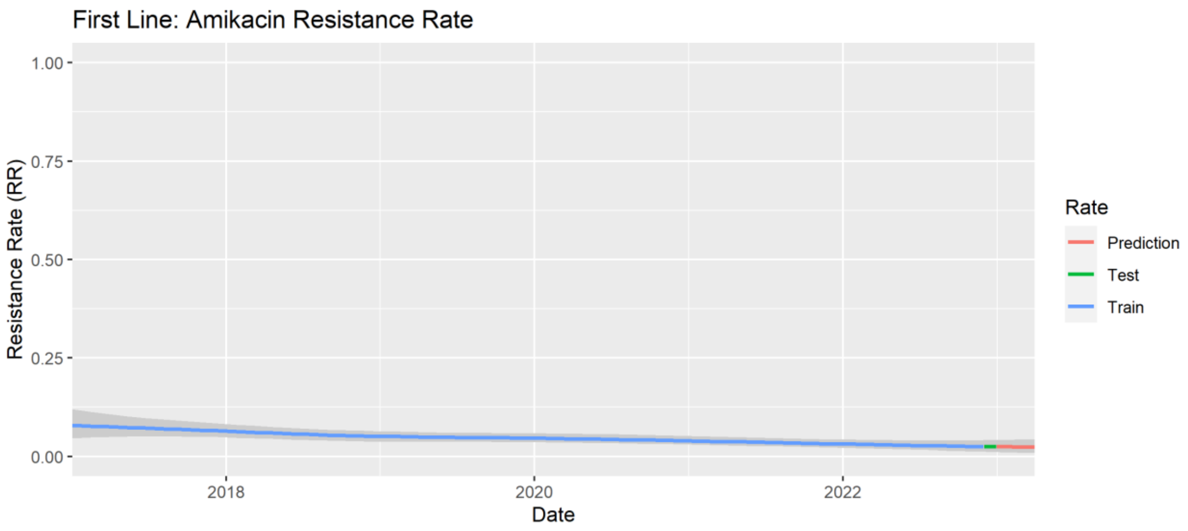

[1] "2017-01-02 Mid 0.079 (95%CI 0.046 to 0.120) Estimated Error 0.0185"

[1] "2022-12-01 Mid 0.025 (95%CI 0.012 to 0.041) Estimated Error 0.0074"

[1] "2022-12-30 Mid 0.025 (95%CI 0.011 to 0.041) Estimated Error 0.0077"

"99.9% posterior probability of decrease "

Over 80

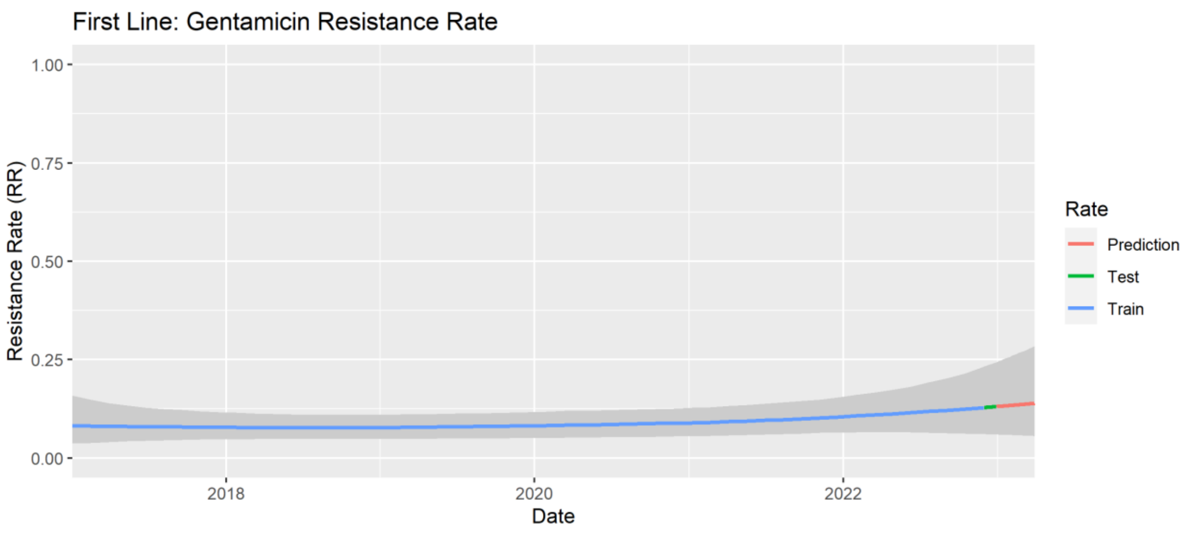

[1] "2017-01-02 Mid 0.082 (95%CI 0.037 to 0.158) Estimated Error 0.0307"

[1] "2022-11-30 Mid 0.128 (95%CI 0.060 to 0.232) Estimated Error 0.0441"

[1] "2022-12-29 Mid 0.130 (95%CI 0.059 to 0.243) Estimated Error 0.0473"

"18.2% posterior probability of decrease "

Under 18

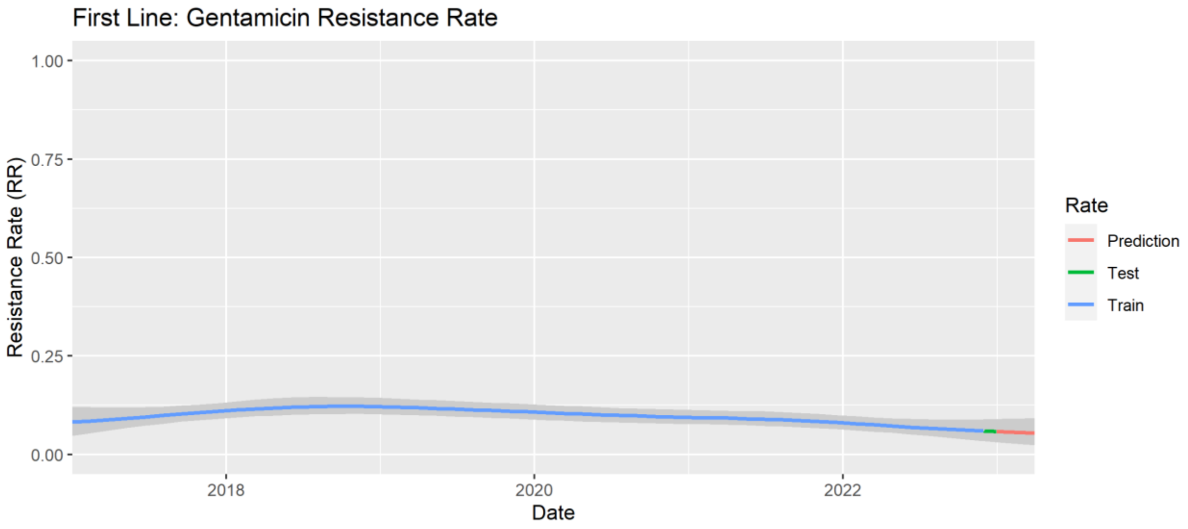

[1] "2017-01-01 Mid 0.082 (95%CI 0.047 to 0.120) Estimated Error 0.0183"

[1] "2022-12-01 Mid 0.059 (95%CI 0.033 to 0.089) Estimated Error 0.0142"

[1] "2022-12-30 Mid 0.058 (95%CI 0.031 to 0.089) Estimated Error 0.0150"

"86.1% posterior probability of decrease "

Over 80

First Line: Ciprofloxacin Resistance Rate

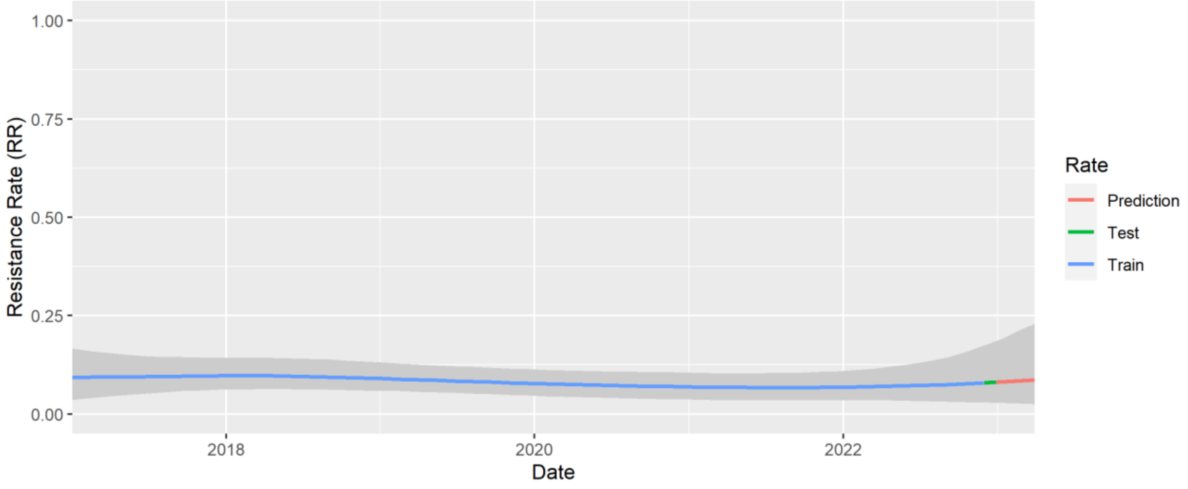

[1] "2017-01-02 Mid 0.093 (95%CI 0.036 to 0.166) Estimated Error 0.0330"

[1] "2022-11-30 Mid 0.079 (95%CI 0.029 to 0.174) Estimated Error 0.0368"

[1] "2022-12-29 Mid 0.081 (95%CI 0.028 to 0.185) Estimated Error 0.0400"

"64.2% posterior probability of decrease "

Under 18

First Line: Gentamicin Resistance Rate

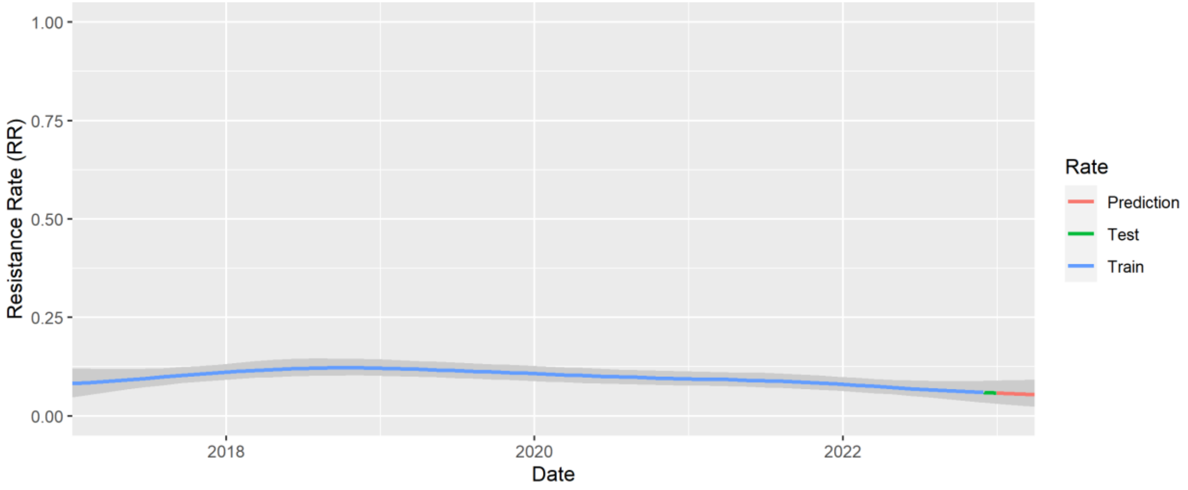

[1] "2017-01-01 Mid 0.141 (95%CI 0.098 to 0.186) Estimated Error 0.0230"

[1] "2022-12-01 Mid 0.111 (95%CI 0.077 to 0.149) Estimated Error 0.0182"

[1] "2022-12-30 Mid 0.110 (95%CI 0.074 to 0.150) Estimated Error 0.0194"

"85.5% posterior probability of decrease "

Over 80

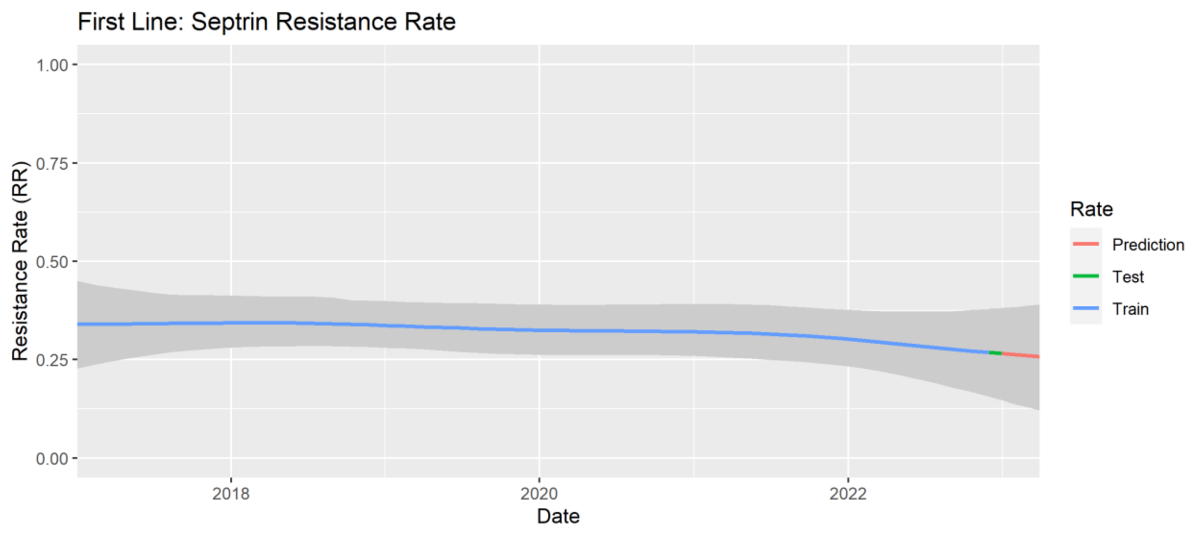

[1] "2017-01-01 Mid 0.336 (95%CI 0.273 to 0.395) Estimated Error 0.0318"

[1] "2022-12-01 Mid 0.293 (95%CI 0.239 to 0.359) Estimated Error 0.0310"

[1] "2022-12-30 Mid 0.294 (95%CI 0.235 to 0.366) Estimated Error 0.0340"

[1] "82.0% posterior probability of decrease "

Under 18

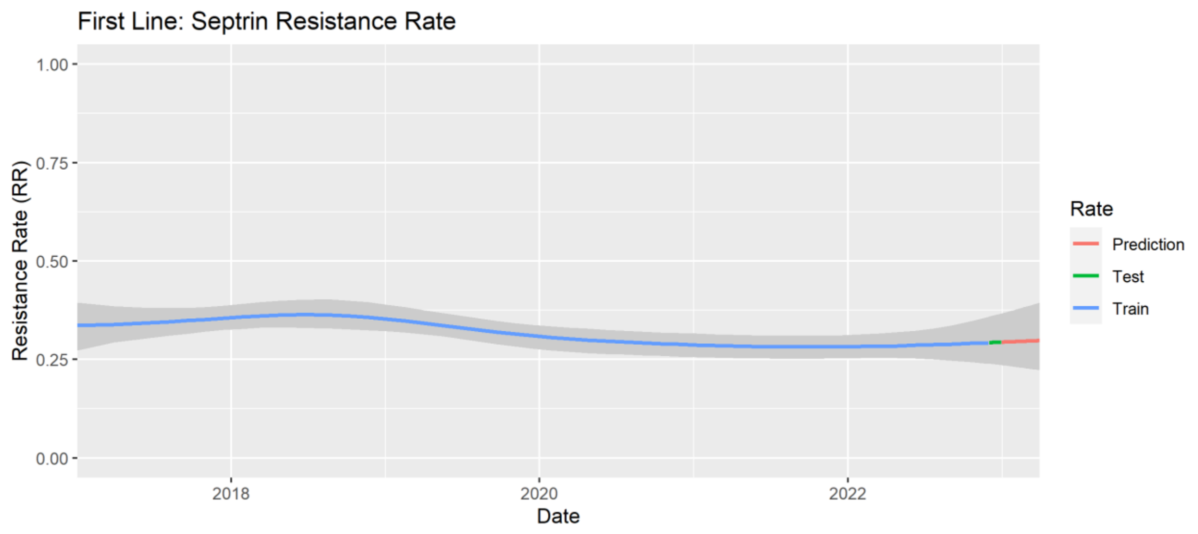

[1] "2017-01-02 Mid 0.339 (95%CI 0.228 to 0.450) Estimated Error 0.0560"

[1] "2022-11-30 Mid 0.268 (95%CI 0.155 to 0.379) Estimated Error 0.0565"

[1] "2022-12-29 Mid 0.265 (95%CI 0.147 to 0.381) Estimated Error 0.0592"

[1] "82.4% posterior probability of decrease "

Over 80

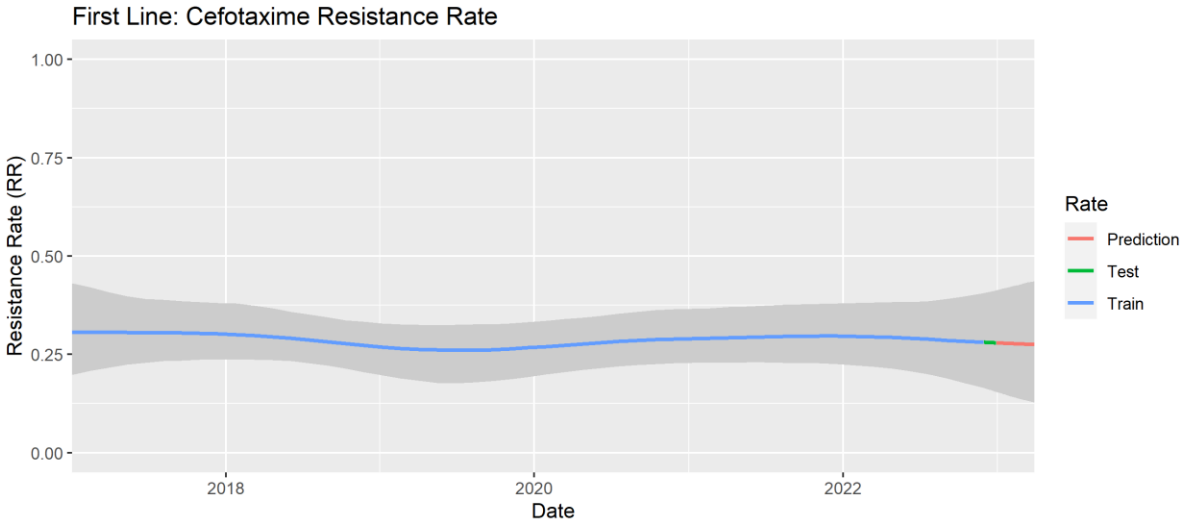

[1] "2017-01-02 Mid 0.307 (95%CI 0.198 to 0.431) Estimated Error 0.0585"

[1] "2022-11-30 Mid 0.281 (95%CI 0.164 to 0.406) Estimated Error 0.0610"

[1] "2022-12-29 Mid 0.279 (95%CI 0.154 to 0.412) Estimated Error 0.0648"

"62.3% posterior probability of decrease "

Under 18

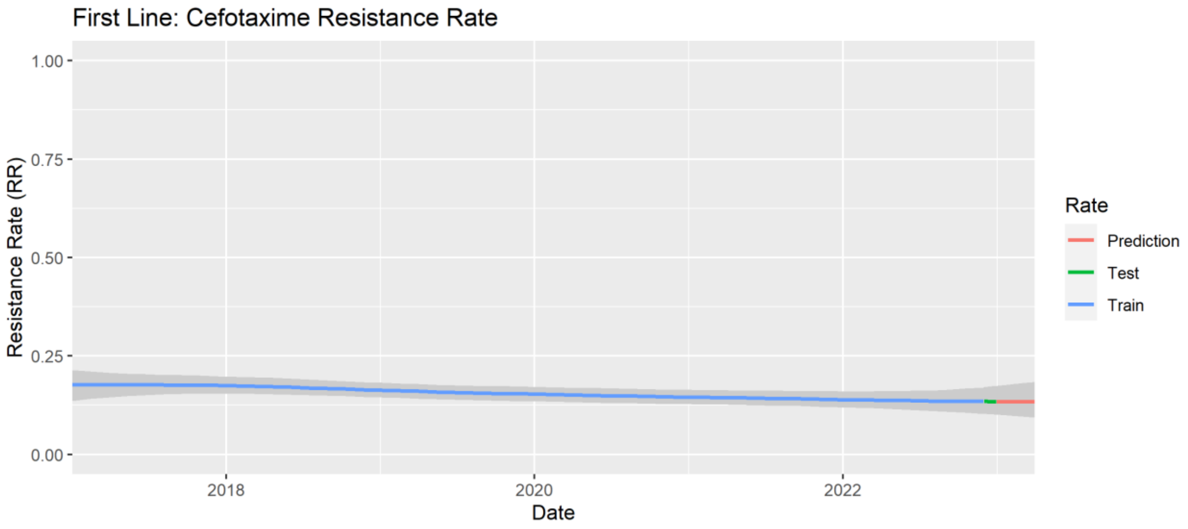

[1] "2017-01-01 Mid 0.176 (95%CI 0.136 to 0.214) Estimated Error 0.0198"

[1] "2022-12-01 Mid 0.134 (95%CI 0.103 to 0.171) Estimated Error 0.0170"

[1] "2022-12-30 Mid 0.134 (95%CI 0.101 to 0.174) Estimated Error 0.0181"

"93.8% posterior probability of decrease "

Over 80

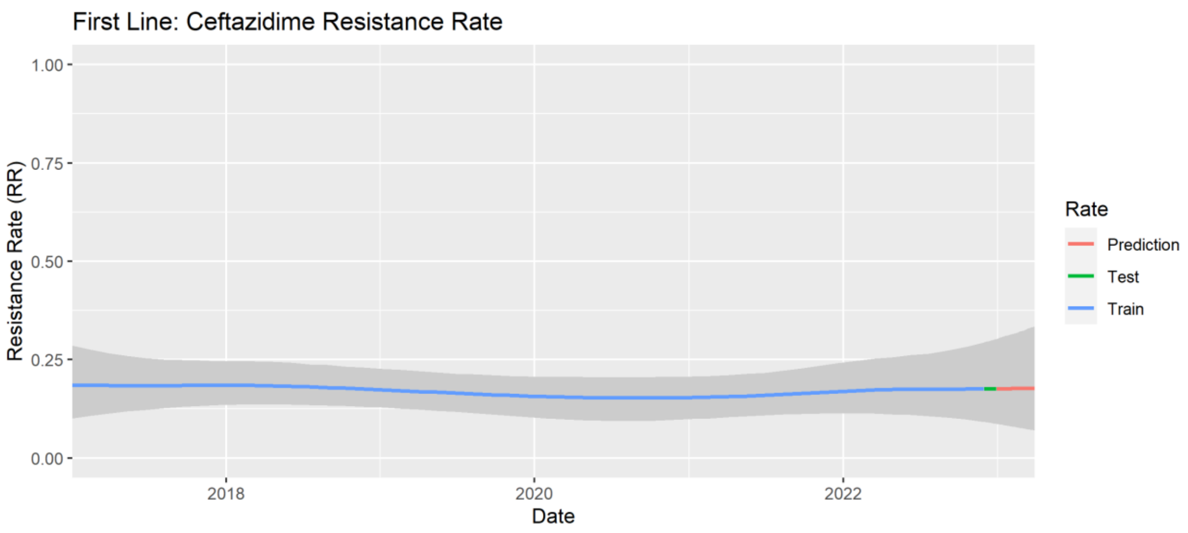

[1] "2017-01-02 Mid 0.185 (95%CI 0.100 to 0.286) Estimated Error 0.0470"

[1] "2022-11-30 Mid 0.175 (95%CI 0.091 to 0.293) Estimated Error 0.0509"

[1] "2022-12-29 Mid 0.176 (95%CI 0.086 to 0.302) Estimated Error 0.0541"

[1] "57.1% posterior probability of decrease "

Under 18

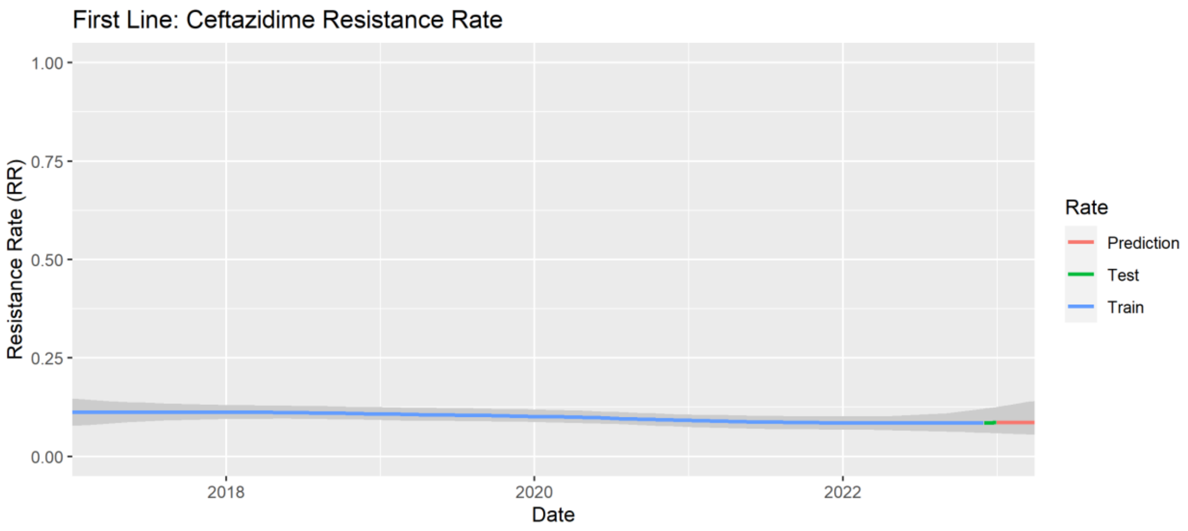

[1] "2017-01-01 Mid 0.113 (95%CI 0.077 to 0.147) Estimated Error 0.0177"

[1] "2022-12-01 Mid 0.085 (95%CI 0.060 to 0.121) Estimated Error 0.0152"

[1] "2022-12-30 Mid 0.085 (95%CI 0.059 to 0.125) Estimated Error 0.0165"

"87.2% posterior probability of decrease "

Over 80

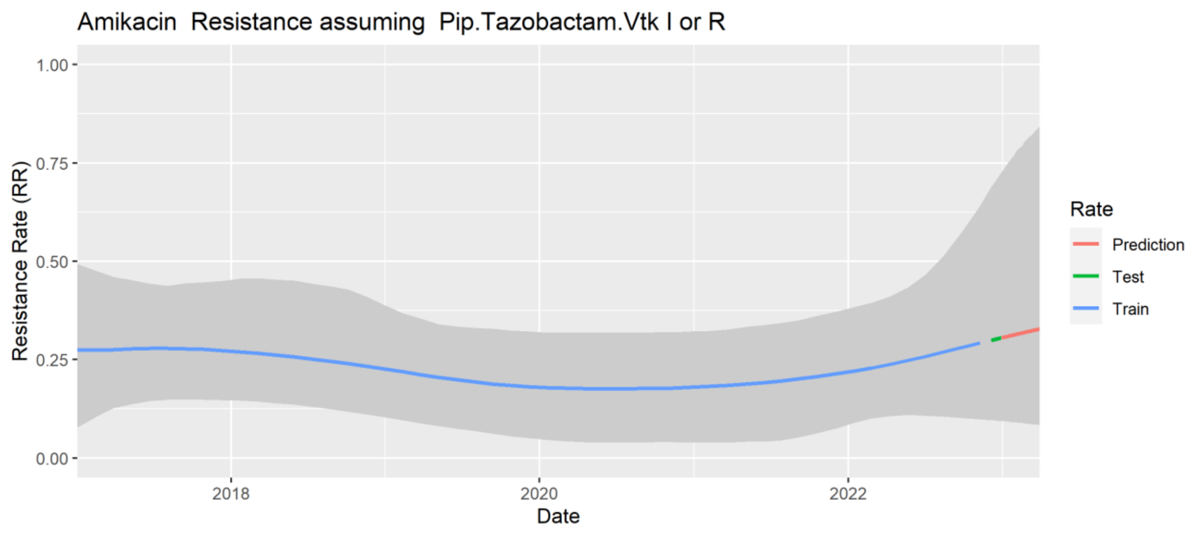

[1] "2017-01-02 Mid 0.274 (95%CI 0.078 to 0.493) Estimated Error 0.1026"

[1] "2022-12-06 Mid 0.299 (95%CI 0.096 to 0.690) Estimated Error 0.1466"

[1] "2022-12-29 Mid 0.305 (95%CI 0.094 to 0.726) Estimated Error 0.1553"

"49.6% posterior probability of decrease "

Under 18

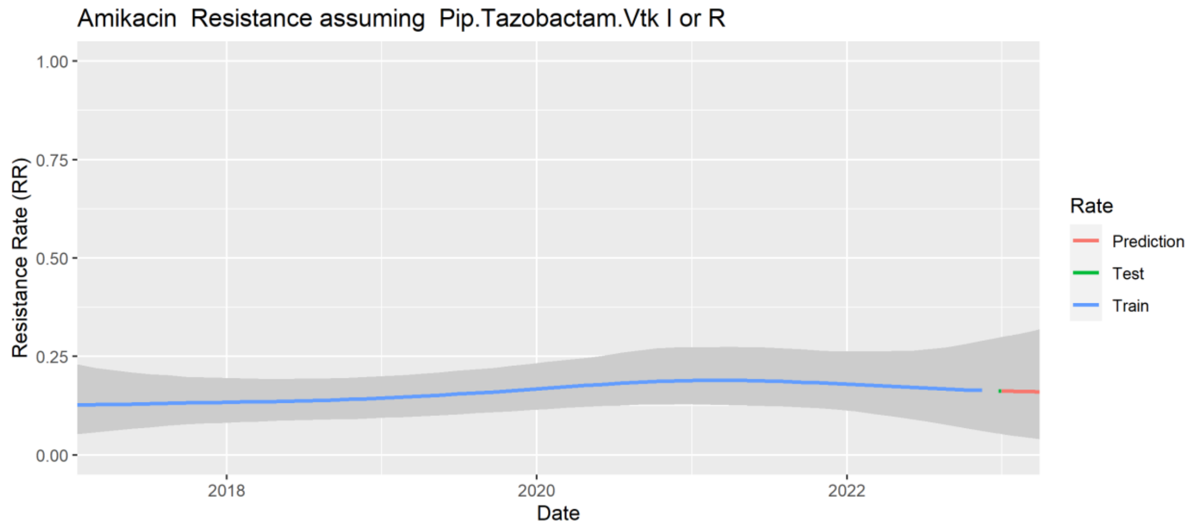

[1] "2017-01-14 Mid 0.127 (95%CI 0.052 to 0.230) Estimated Error 0.0450"

[1] "2022-12-24 Mid 0.162 (95%CI 0.054 to 0.298) Estimated Error 0.0629"

[1] "2022-12-30 Mid 0.162 (95%CI 0.053 to 0.299) Estimated Error 0.0635"

"32.5% posterior probability of decrease "

Over 80

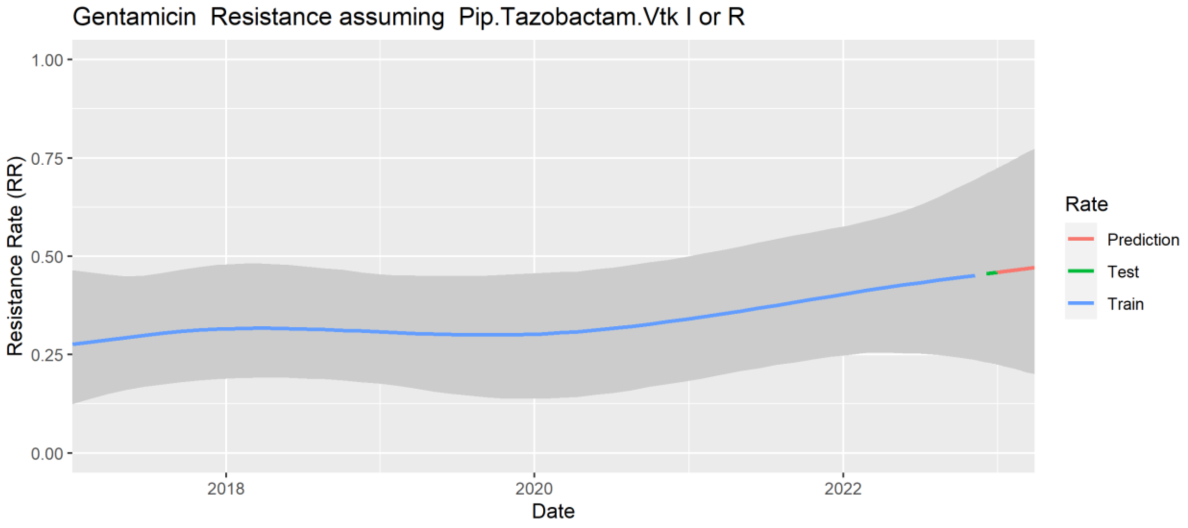

[1] "2017-01-02 Mid 0.277 (95%CI 0.124 to 0.465) Estimated Error 0.0875"

[1] "2022-12-06 Mid 0.455 (95%CI 0.230 to 0.711) Estimated Error 0.1235"

[1] "2022-12-29 Mid 0.459 (95%CI 0.225 to 0.723) Estimated Error 0.1283"

"12.6% posterior probability of decrease "

Under 18

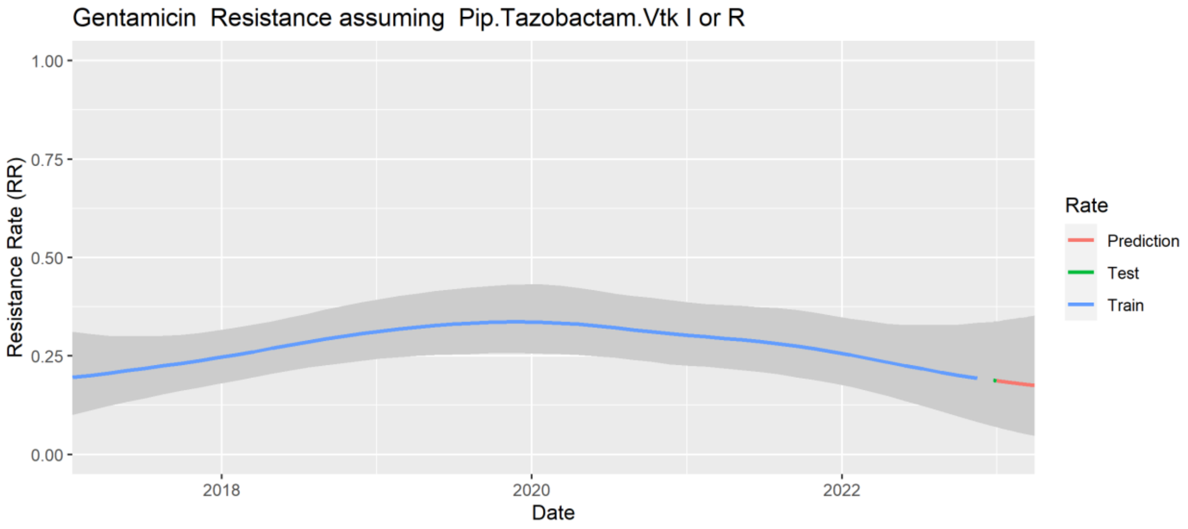

[1] "2017-01-14 Mid 0.196 (95%CI 0.101 to 0.311) Estimated Error 0.0544"

[1] "2022-12-24 Mid 0.188 (95%CI 0.071 to 0.337) Estimated Error 0.0700"

[1] "2022-12-30 Mid 0.187 (95%CI 0.069 to 0.338) Estimated Error 0.0706"

"53.8% posterior probability of decrease "

Over 80

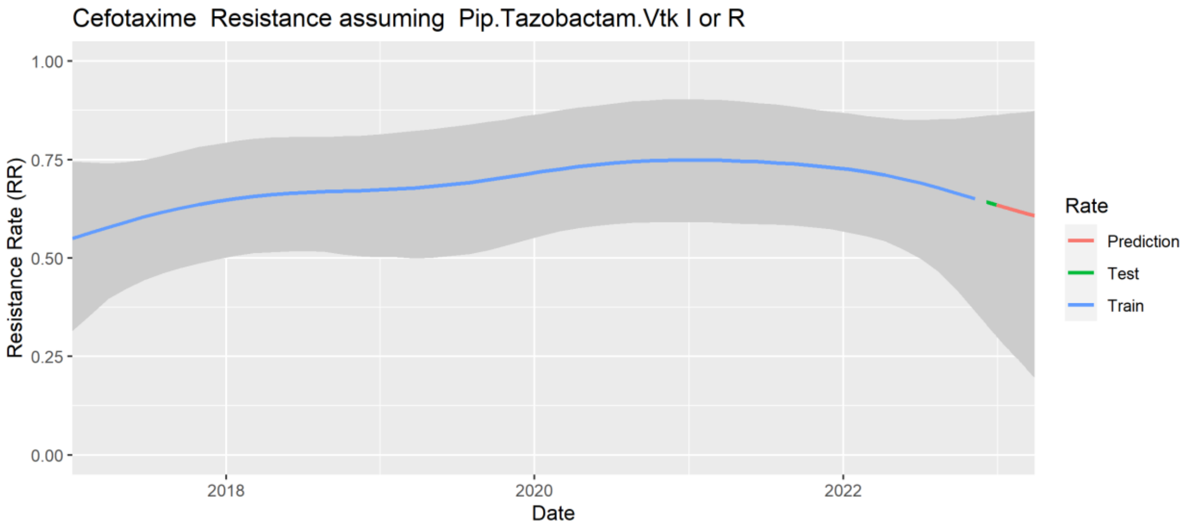

[1] "2017-01-02 Mid 0.550 (95%CI 0.315 to 0.745) Estimated Error 0.1097"

[1] "2022-12-06 Mid 0.642 (95%CI 0.330 to 0.861) Estimated Error 0.1364"

[1] "2022-12-29 Mid 0.635 (95%CI 0.302 to 0.863) Estimated Error 0.1449"

"25.8% posterior probability of decrease "

Under 18

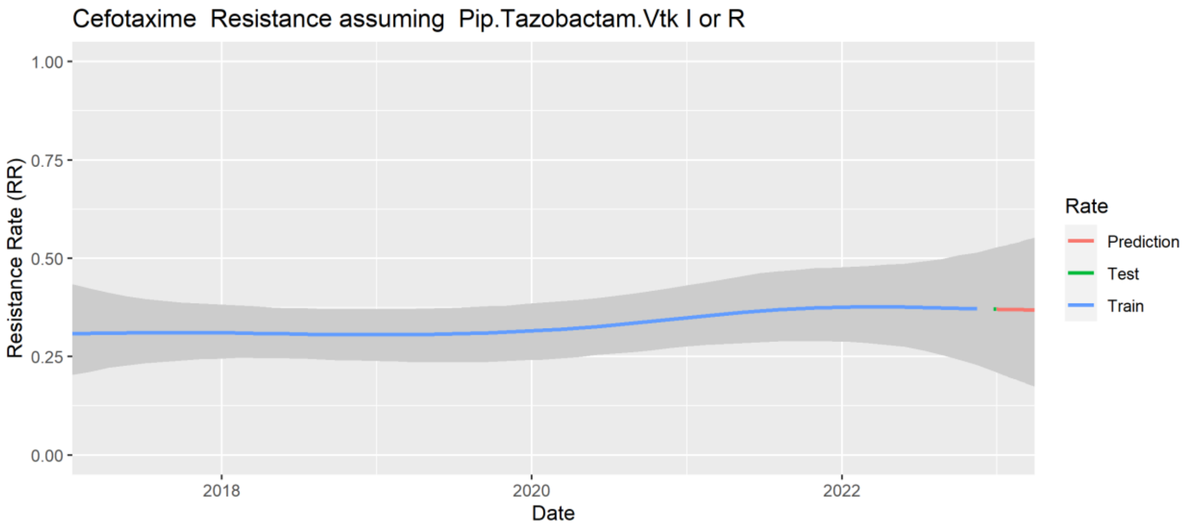

[1] "2017-01-14 Mid 0.309 (95%CI 0.204 to 0.434) Estimated Error 0.0583"

[1] "2022-12-24 Mid 0.370 (95%CI 0.213 to 0.526) Estimated Error 0.0782"

[1] "2022-12-30 Mid 0.370 (95%CI 0.210 to 0.527) Estimated Error 0.0791"

"28.0% posterior probability of decrease "

Over 80

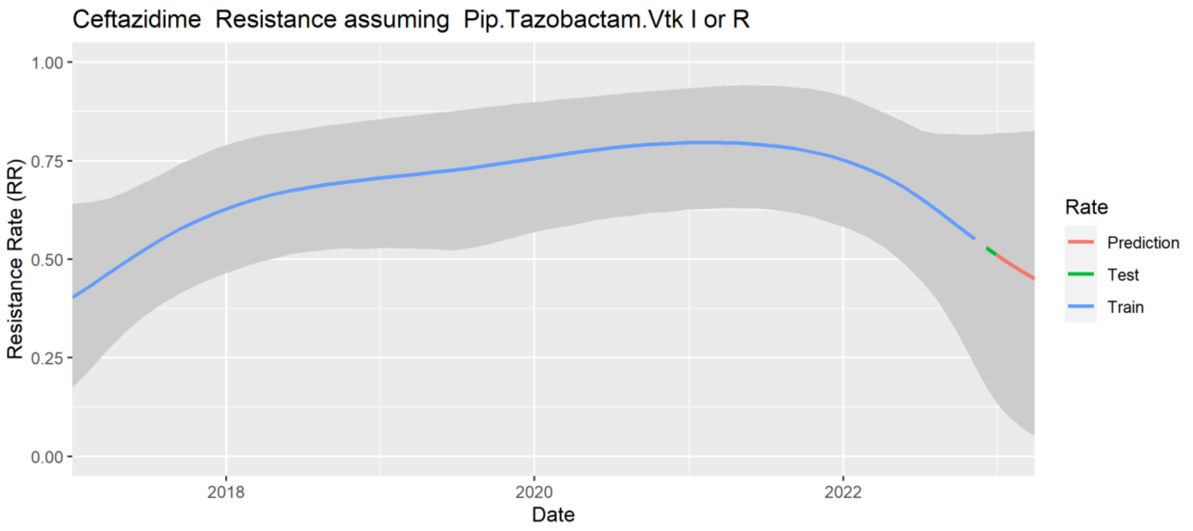

[1] "2017-01-02 Mid 0.404 (95%CI 0.175 to 0.641) Estimated Error 0.1219"

[1] "2022-12-06 Mid 0.529 (95%CI 0.174 to 0.817) Estimated Error 0.1687"

[1] "2022-12-29 Mid 0.511 (95%CI 0.138 to 0.819) Estimated Error 0.1795"

[1] "24.7% posterior probability of decrease "

Under 18

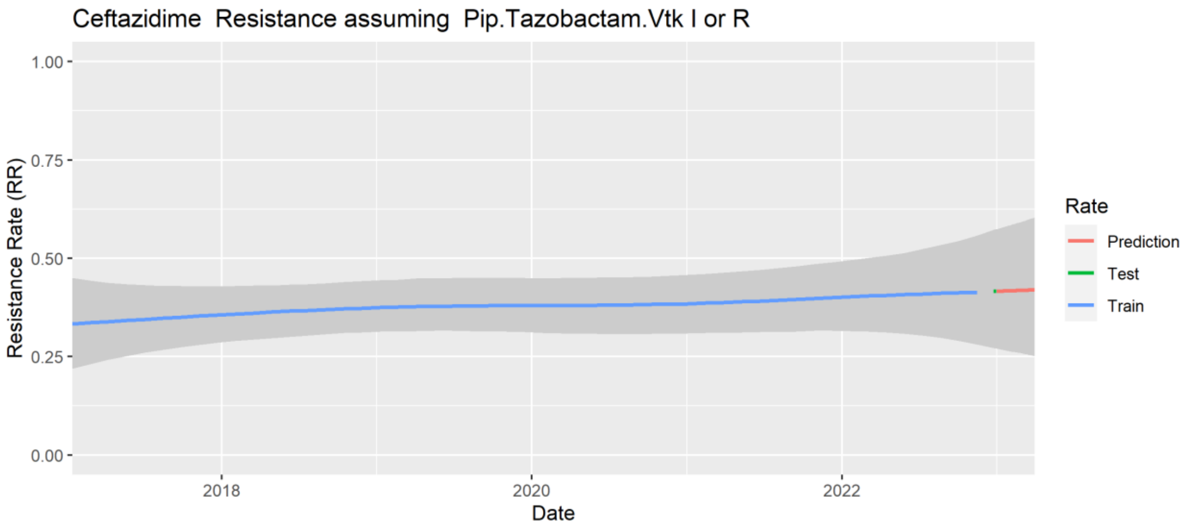

[1] "2017-01-14 Mid 0.333 (95%CI 0.219 to 0.450) Estimated Error 0.0585"

[1] "2022-12-24 Mid 0.416 (95%CI 0.271 to 0.572) Estimated Error 0.0749"

[1] "2022-12-30 Mid 0.416 (95%CI 0.270 to 0.574) Estimated Error 0.0757"

[1] "20.4% posterior probability of decrease "

Over 80

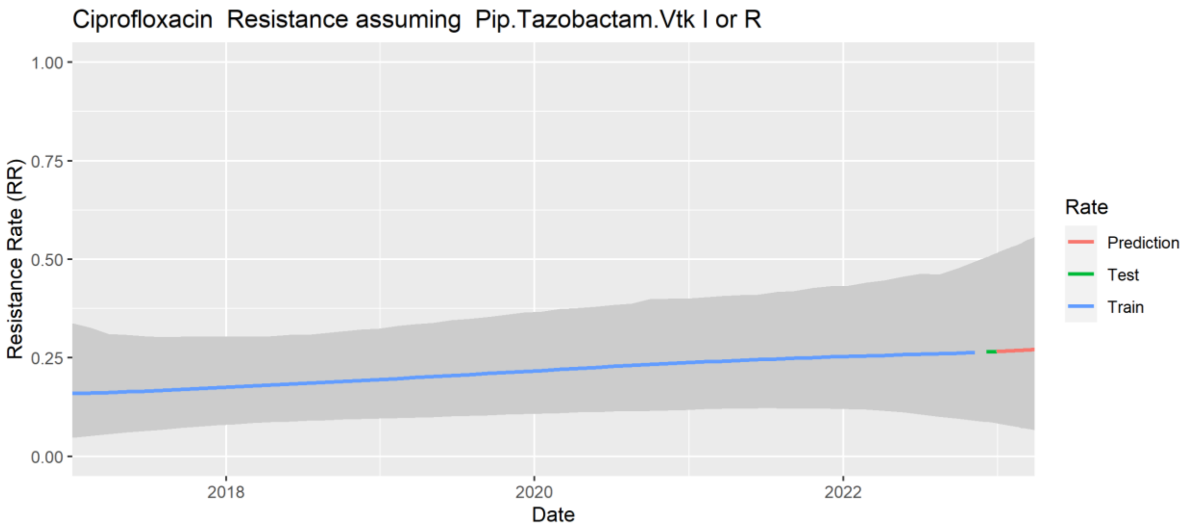

[1] "2017-01-02 Mid 0.160 (95%CI 0.048 to 0.338) Estimated Error 0.0750"

[1] "2022-12-06 Mid 0.265 (95%CI 0.087 to 0.505) Estimated Error 0.1091"

[1] "2022-12-29 Mid 0.266 (95%CI 0.084 to 0.515) Estimated Error 0.1126"

"22.4% posterior probability of decrease "

Under 18

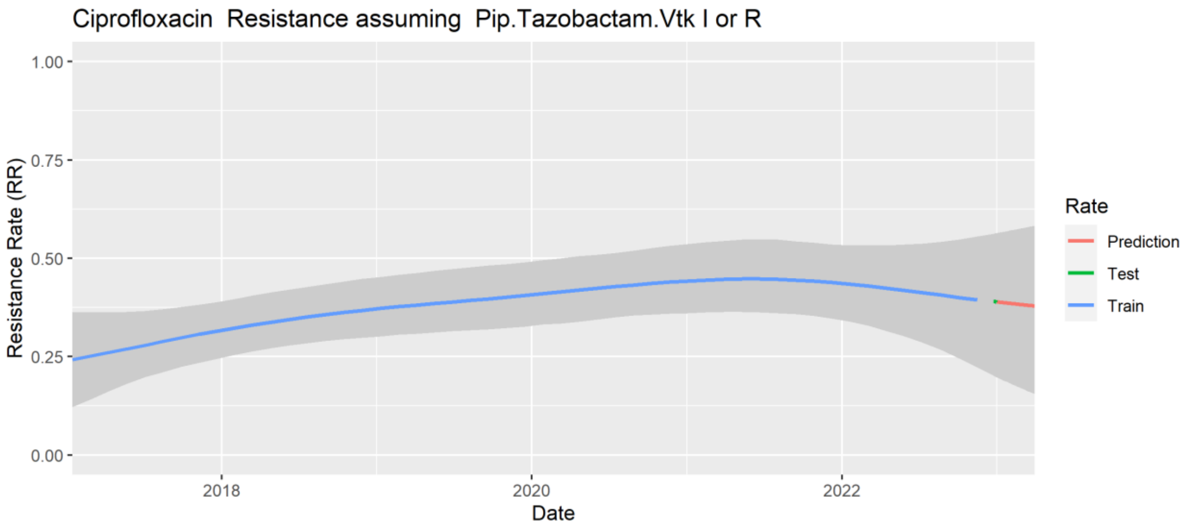

[1] "2017-01-14 Mid 0.242 (95%CI 0.122 to 0.362) Estimated Error 0.0612"

[1] "2022-12-24 Mid 0.390 (95%CI 0.201 to 0.562) Estimated Error 0.0938"

[1] "2022-12-30 Mid 0.389 (95%CI 0.198 to 0.564) Estimated Error 0.0950"

"7.4% posterior probability of decrease "

Over 80

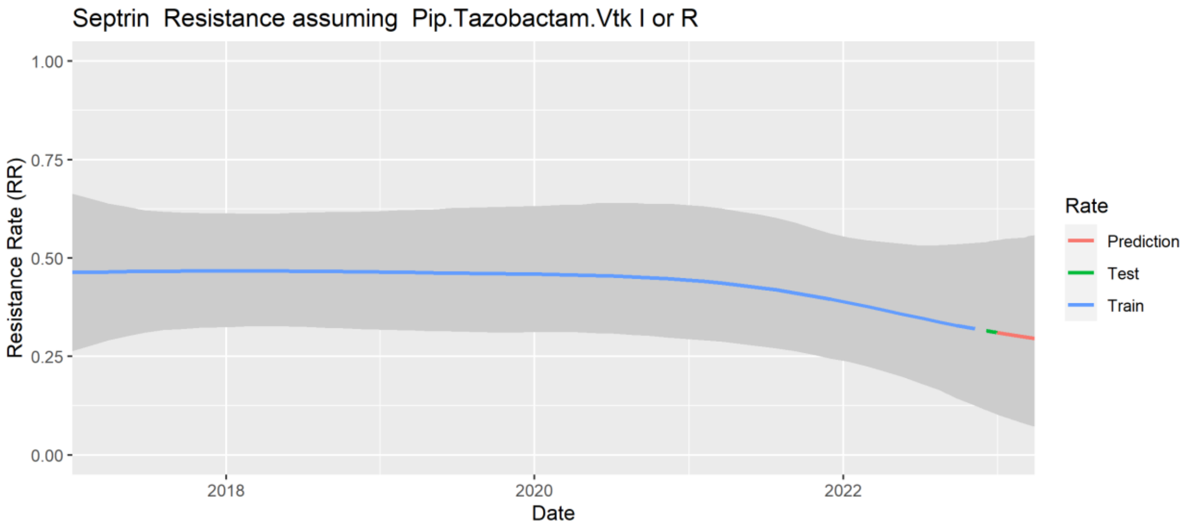

[1] "2017-01-02 Mid 0.463 (95%CI 0.264 to 0.664) Estimated Error 0.1016"

[1] "2022-12-06 Mid 0.315 (95%CI 0.113 to 0.541) Estimated Error 0.1111"

[1] "2022-12-29 Mid 0.311 (95%CI 0.103 to 0.545) Estimated Error 0.1144"

"83.2% posterior probability of decrease "

Under 18

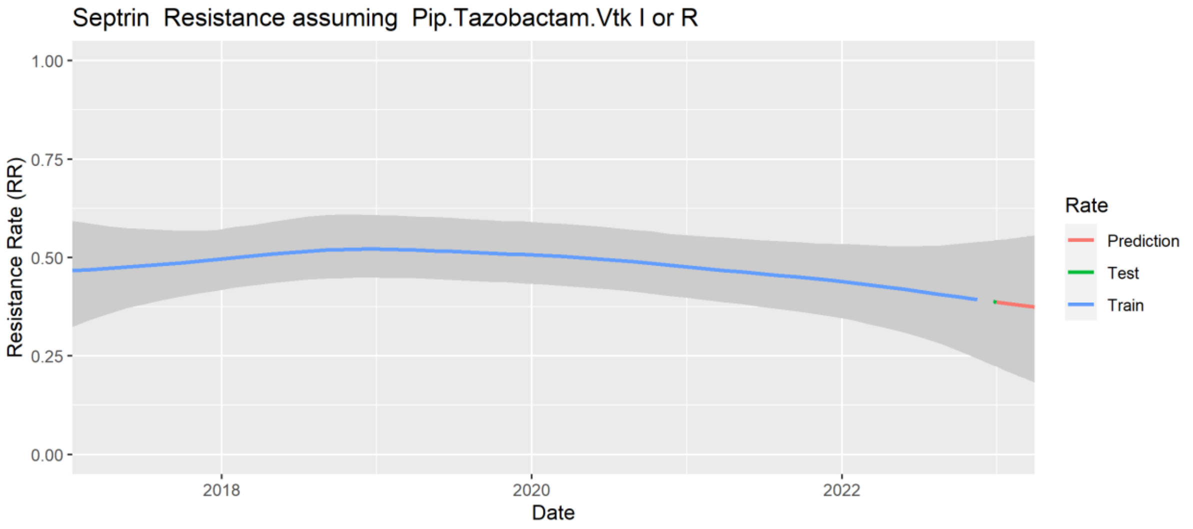

[1] "2017-01-14 Mid 0.467 (95%CI 0.324 to 0.594) Estimated Error 0.0682"

[1] "2022-12-24 Mid 0.387 (95%CI 0.225 to 0.543) Estimated Error 0.0804"

[1] "2022-12-30 Mid 0.387 (95%CI 0.223 to 0.544) Estimated Error 0.0813"

[1] "78.2% posterior probability of decrease "

Over 80

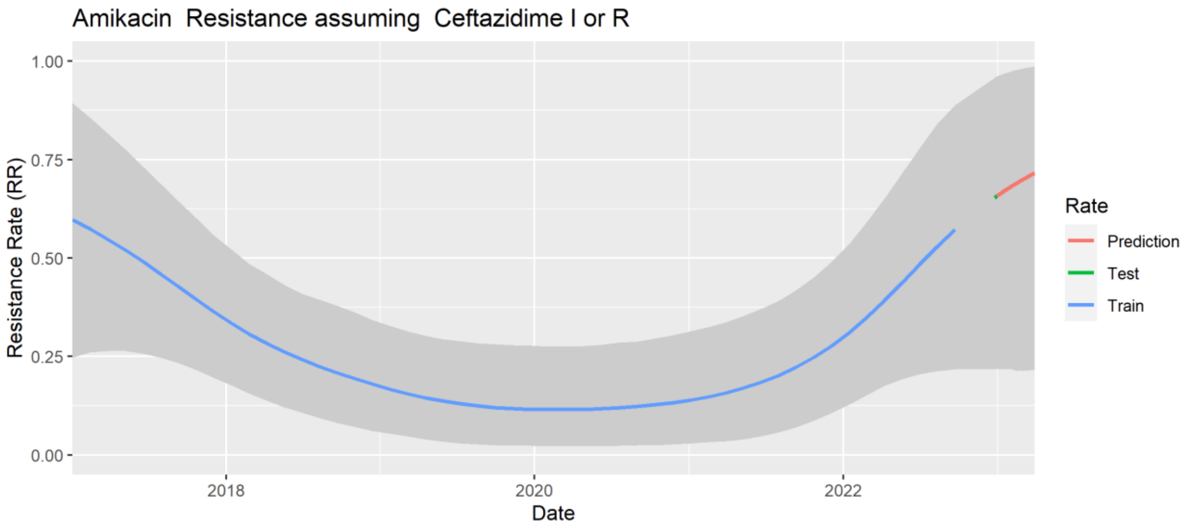

1] "2017-01-02 Mid 0.597 (95%CI 0.247 to 0.893) Estimated Error 0.1729"

[1] "2022-12-26 Mid 0.655 (95%CI 0.218 to 0.958) Estimated Error 0.2024"

[1] "2022-12-29 Mid 0.657 (95%CI 0.218 to 0.960) Estimated Error 0.2030"

"40.5% posterior probability of decrease "

Under 18

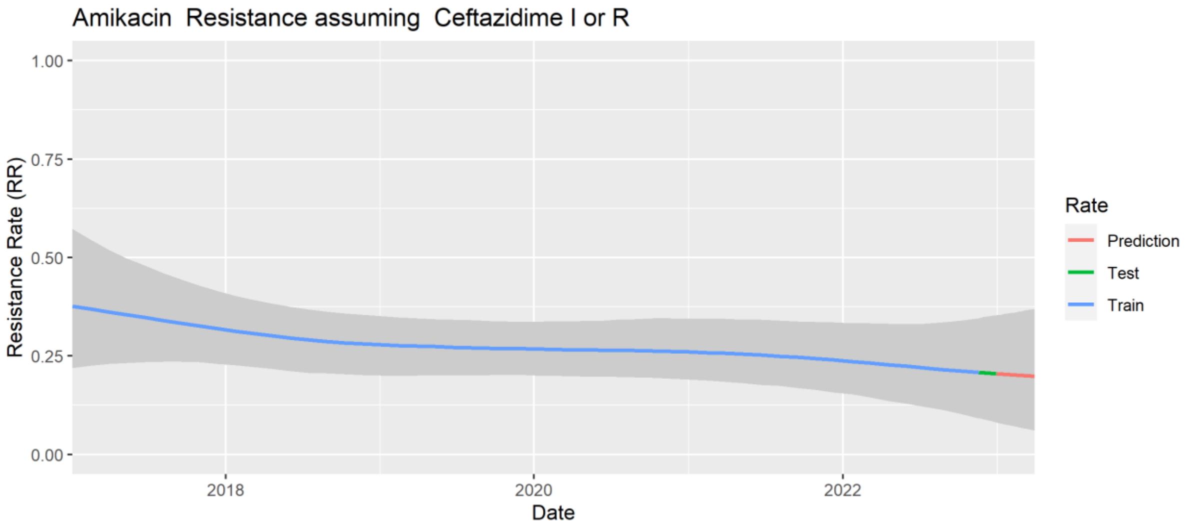

[1] "2017-01-03 Mid 0.376 (95%CI 0.219 to 0.573) Estimated Error 0.0889"

[1] "2022-11-19 Mid 0.208 (95%CI 0.092 to 0.346) Estimated Error 0.0642"

[1] "2022-12-30 Mid 0.205 (95%CI 0.081 to 0.353) Estimated Error 0.0681"

"93.5% posterior probability of decrease "

Over 80

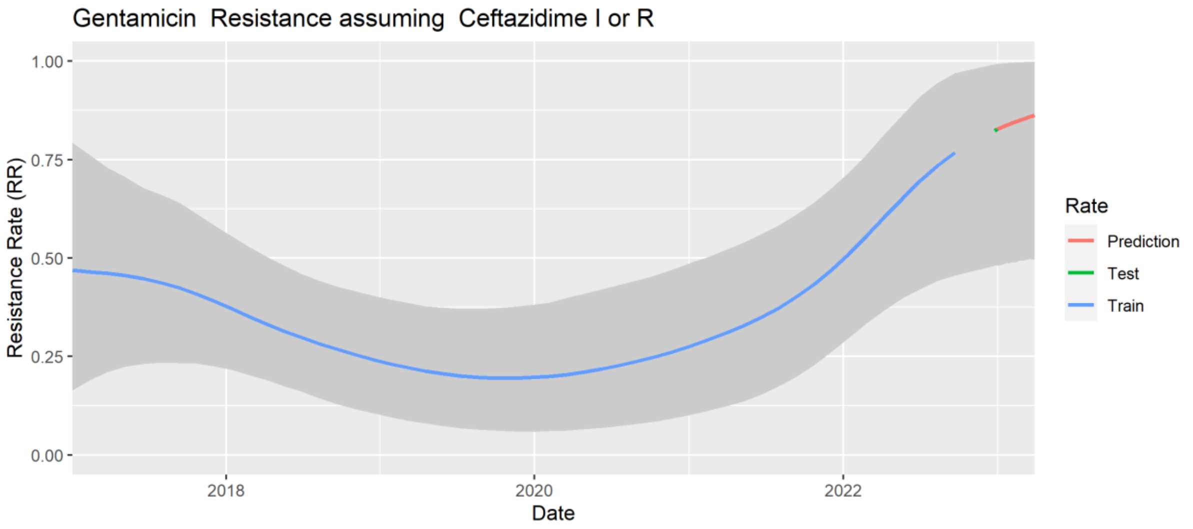

[1] "2017-01-02 Mid 0.469 (95%CI 0.164 to 0.792) Estimated Error 0.1662"

[1] "2022-12-26 Mid 0.825 (95%CI 0.480 to 0.992) Estimated Error 0.1392"

[1] "2022-12-29 Mid 0.826 (95%CI 0.480 to 0.993) Estimated Error 0.1392"

"4.9% posterior probability of decrease "

Under 18

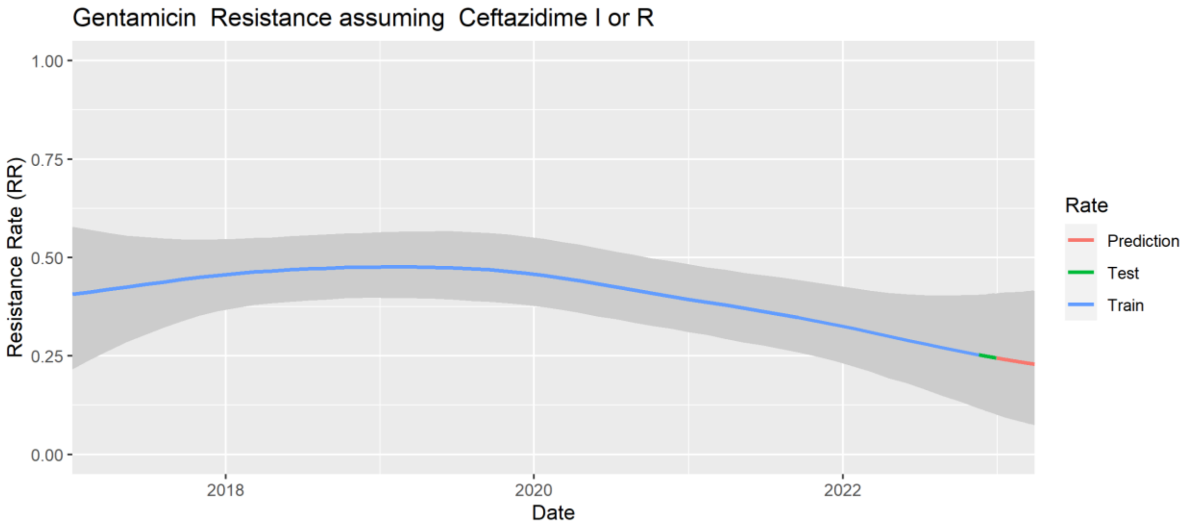

[1] "2017-01-03 Mid 0.407 (95%CI 0.217 to 0.578) Estimated Error 0.0940"

[1] "2022-11-19 Mid 0.253 (95%CI 0.116 to 0.405) Estimated Error 0.0743"

[1] "2022-12-30 Mid 0.245 (95%CI 0.101 to 0.408) Estimated Error 0.0789"

"91.6% posterior probability of decrease "

Over 80

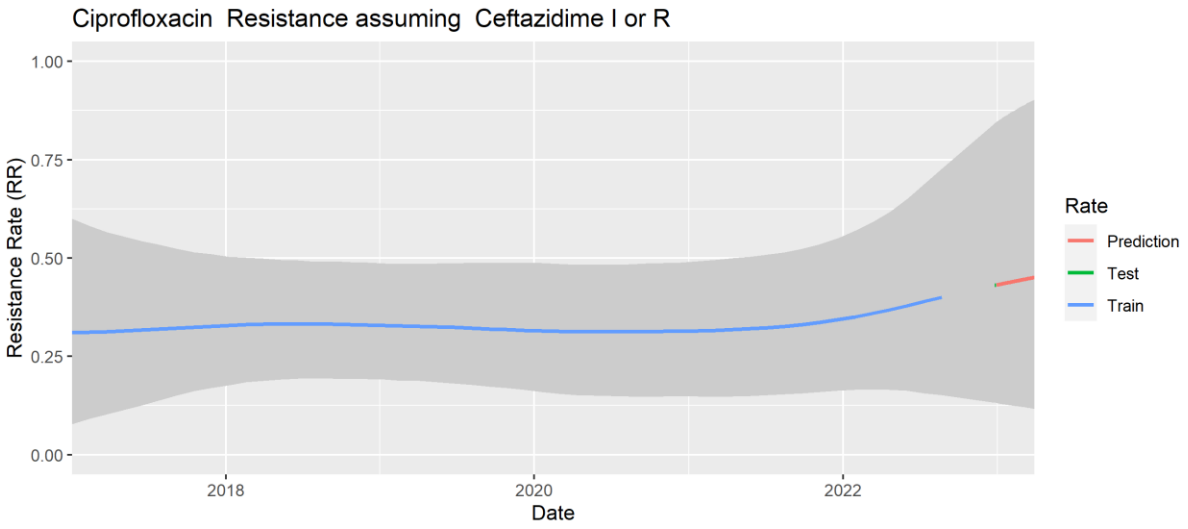

[1] "2017-01-02 Mid 0.310 (95%CI 0.078 to 0.600) Estimated Error 0.1370"

[1] "2022-12-26 Mid 0.431 (95%CI 0.133 to 0.843) Estimated Error 0.1860"

[1] "2022-12-29 Mid 0.431 (95%CI 0.132 to 0.845) Estimated Error 0.1868"

[1] "32.0% posterior probability of decrease "

Under 18

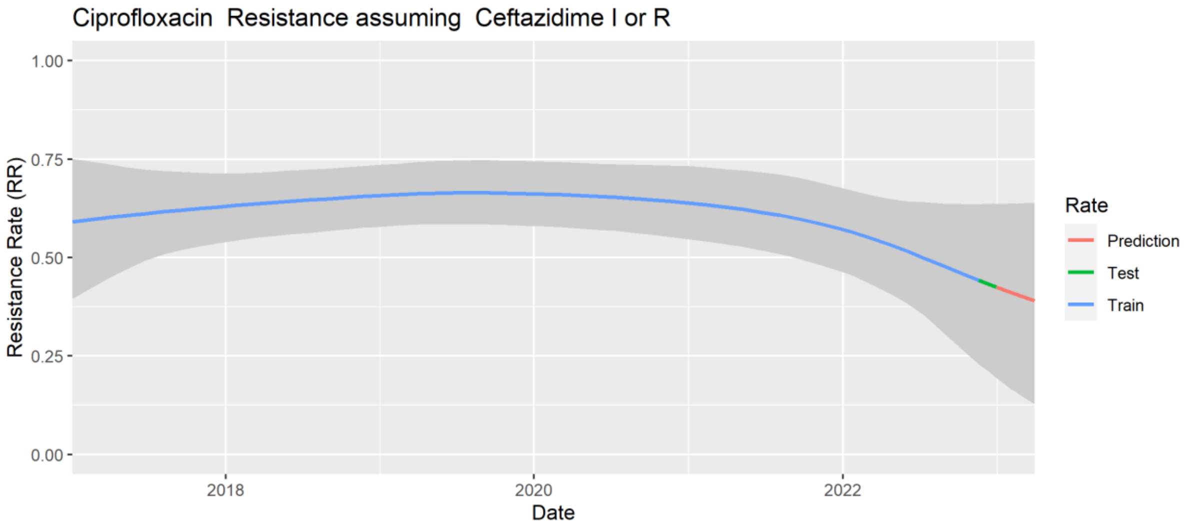

[1] "2017-01-03 Mid 0.591 (95%CI 0.396 to 0.751) Estimated Error 0.0904"

[1] "2022-11-19 Mid 0.442 (95%CI 0.228 to 0.635) Estimated Error 0.1048"

[1] "2022-12-30 Mid 0.425 (95%CI 0.194 to 0.636) Estimated Error 0.1147"

[1] "88.7% posterior probability of decrease "

Over 80

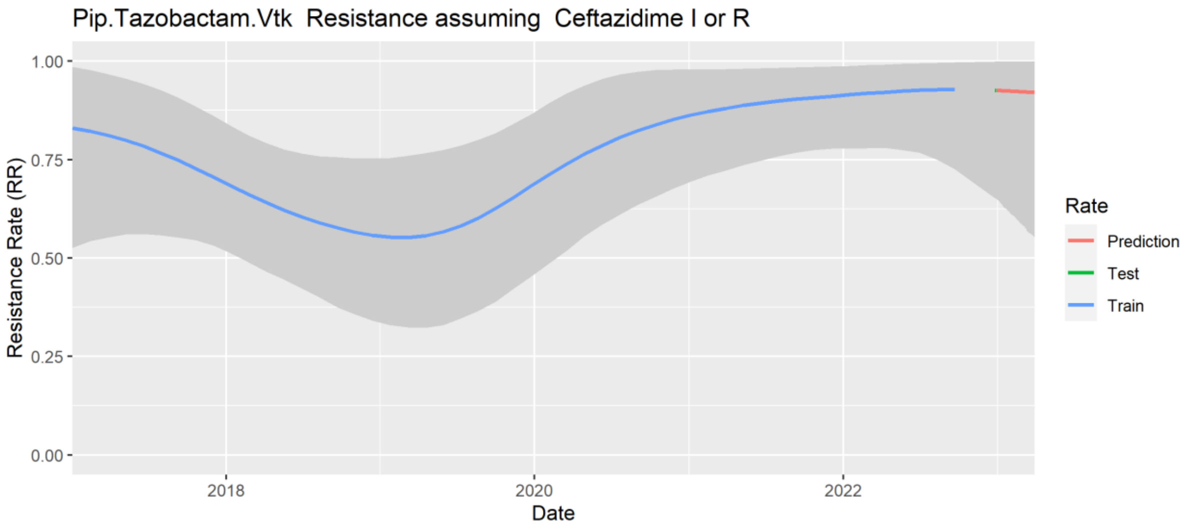

[1] "2017-01-02 Mid 0.830 (95%CI 0.526 to 0.985) Estimated Error 0.1224"

[1] "2022-12-26 Mid 0.926 (95%CI 0.652 to 0.999) Estimated Error 0.0948"

[1] "2022-12-29 Mid 0.925 (95%CI 0.651 to 0.999) Estimated Error 0.0956"

"21.0% posterior probability of decrease "

Under 18

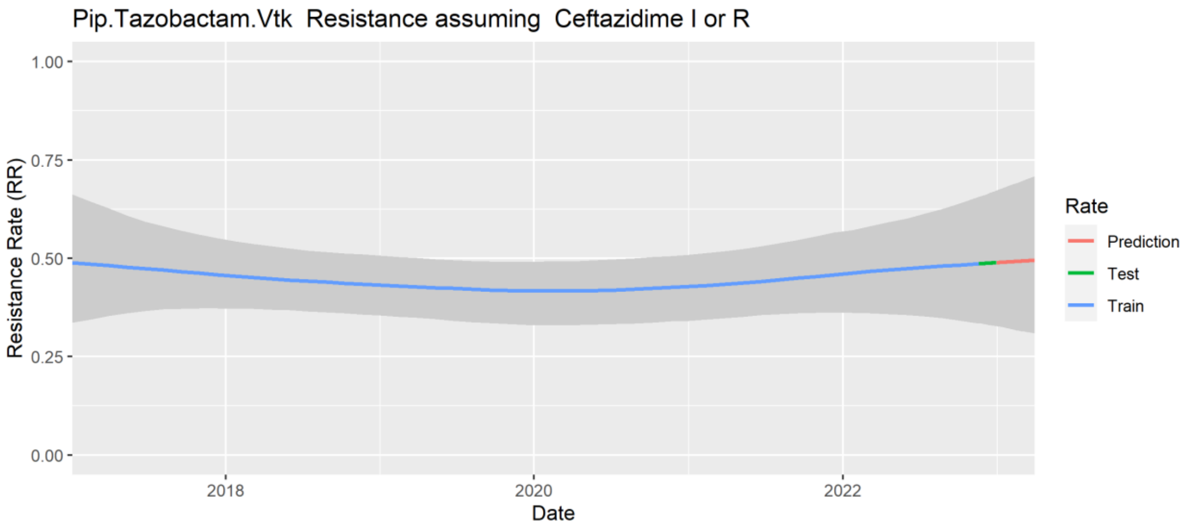

[1] "2017-01-03 Mid 0.489 (95%CI 0.337 to 0.662) Estimated Error 0.0833"

[1] "2022-11-19 Mid 0.486 (95%CI 0.335 to 0.656) Estimated Error 0.0822"

[1] "2022-12-30 Mid 0.489 (95%CI 0.327 to 0.672) Estimated Error 0.0883"

"50.8% posterior probability of decrease "

Over 80

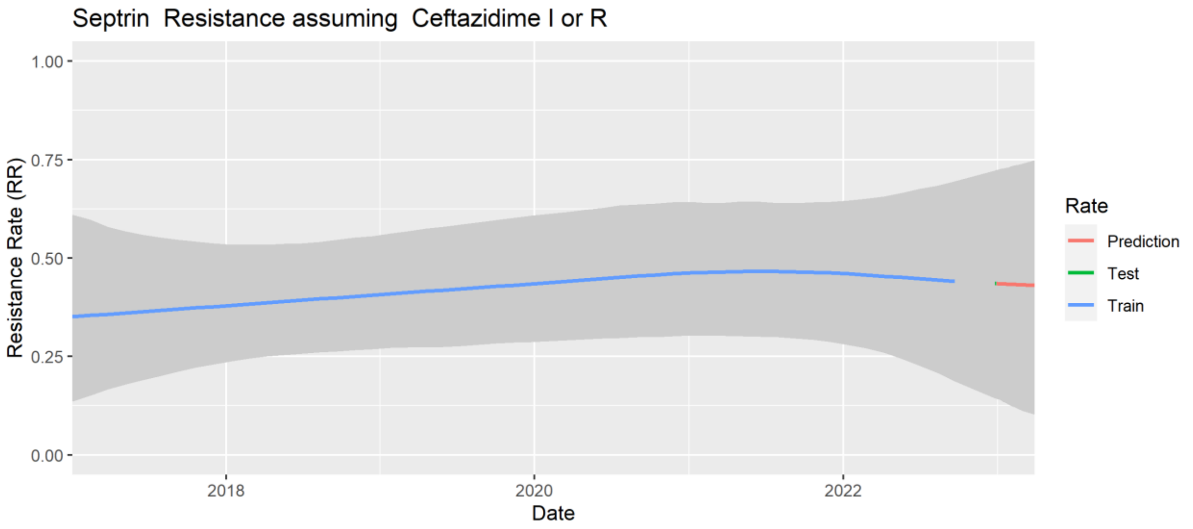

[1] "2017-01-02 Mid 0.352 (95%CI 0.136 to 0.610) Estimated Error 0.1221"

[1] "2022-12-26 Mid 0.435 (95%CI 0.144 to 0.723) Estimated Error 0.1481"

[1] "2022-12-29 Mid 0.435 (95%CI 0.142 to 0.723) Estimated Error 0.1487"

[1] "33.3% posterior probability of decrease "

Under 18

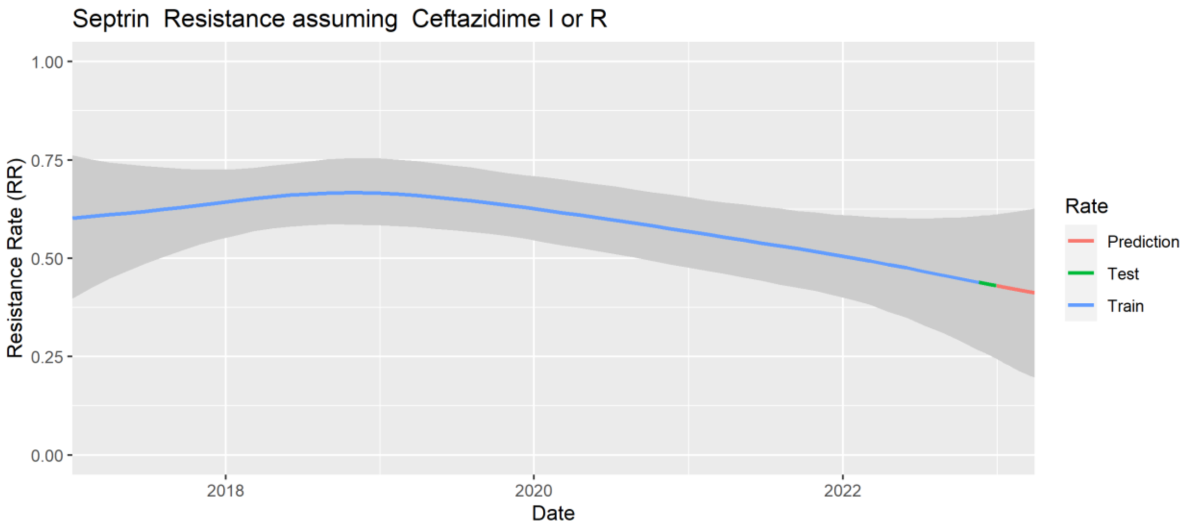

[1] "2017-01-03 Mid 0.602 (95%CI 0.398 to 0.762) Estimated Error 0.0940"

[1] "2022-11-19 Mid 0.439 (95%CI 0.265 to 0.608) Estimated Error 0.0874"

[1] "2022-12-30 Mid 0.430 (95%CI 0.245 to 0.612) Estimated Error 0.0944"

"90.5% posterior probability of decrease "

Over 80

Supplementary Material

Antibiotic resistance rate over time ICU or Haematology/oncology patients

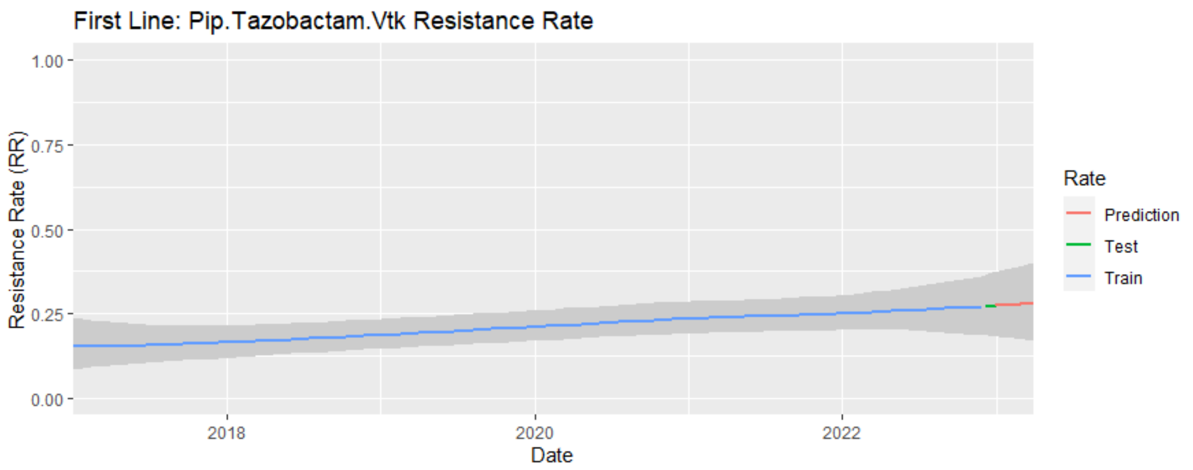

[1] "2017-01-01 Mid 0.151 (95%CI 0.085 to 0.239) Estimated Error 0.0383"

[1] "2022-12-06 Mid 0.271 (95%CI 0.184 to 0.365) Estimated Error 0.0456"

[1] "2022-12-29 Mid 0.273 (95%CI 0.181 to 0.372) Estimated Error 0.0478"

"2.9% posterior probability of decrease "

ICU patients

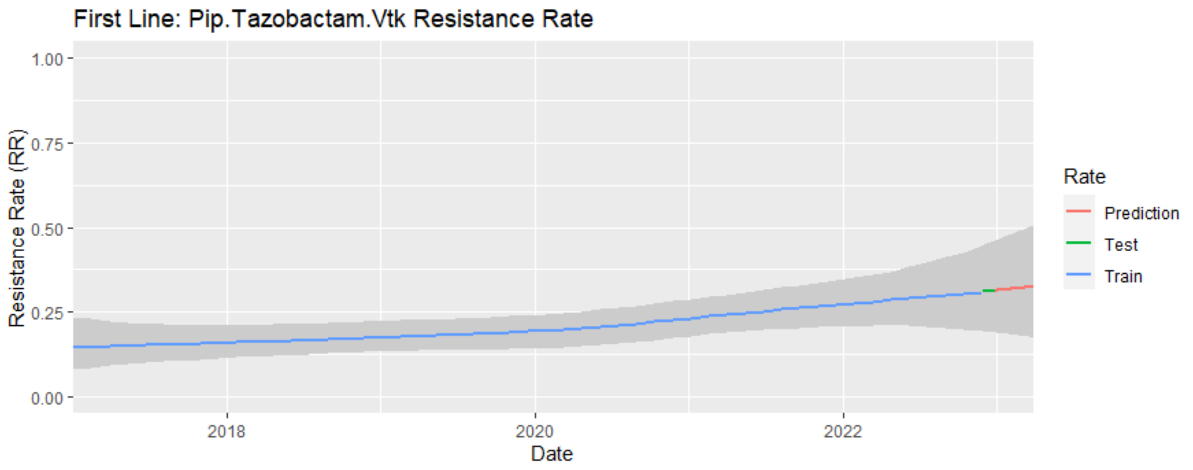

[1] "2017-01-02 Mid 0.145 (95%CI 0.081 to 0.235) Estimated Error 0.0394"

[1] "2022-11-30 Mid 0.310 (95%CI 0.192 to 0.447) Estimated Error 0.0644"

[1] "2022-12-28 Mid 0.313 (95%CI 0.188 to 0.459) Estimated Error 0.0683"

[1] "1.5% posterior probability of decrease "

Haematology/oncology patients

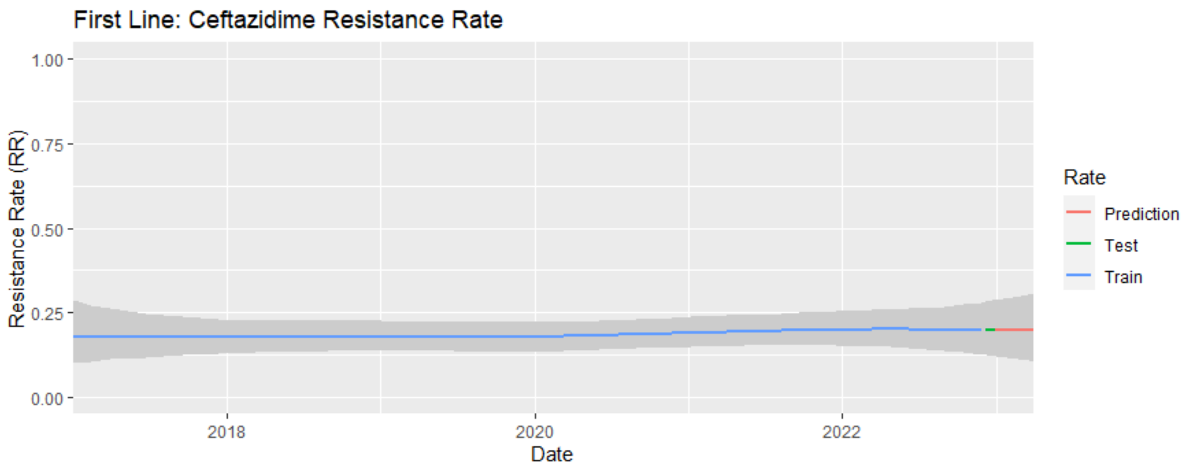

[1] "2017-01-01 Mid 0.179 (95%CI 0.101 to 0.285) Estimated Error 0.0462"

[1] "2022-12-06 Mid 0.199 (95%CI 0.123 to 0.282) Estimated Error 0.0403"

[1] "2022-12-29 Mid 0.199 (95%CI 0.119 to 0.286) Estimated Error 0.0421"

"35.6% posterior probability of decrease "

ICU patients

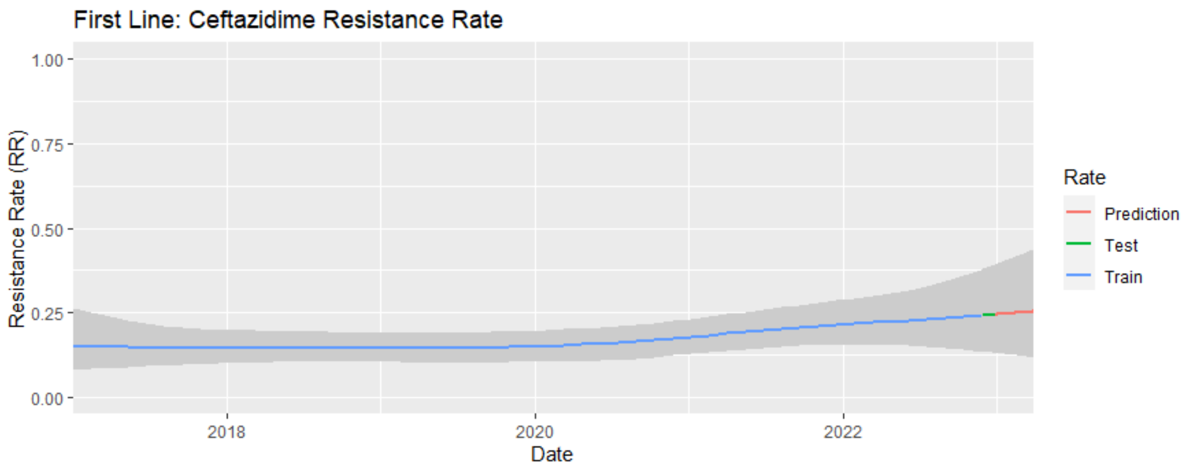

[1] "2017-01-02 Mid 0.152 (95%CI 0.078 to 0.264) Estimated Error 0.0479"

[1] "2022-11-30 Mid 0.242 (95%CI 0.134 to 0.379) Estimated Error 0.0615"

[1] "2022-12-28 Mid 0.244 (95%CI 0.130 to 0.391) Estimated Error 0.0655"

"11.9% posterior probability of decrease "

Haematology/oncology patients

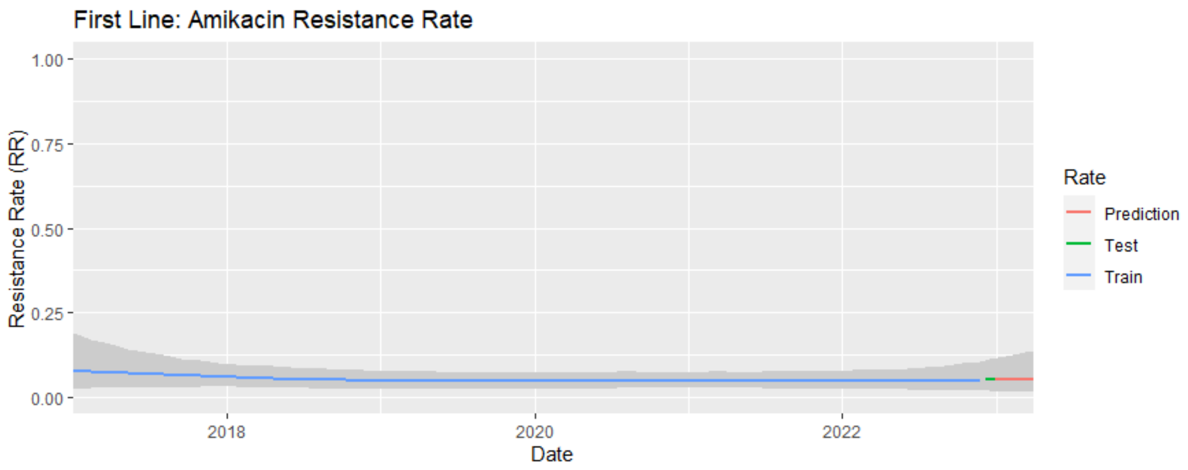

[1] "2017-01-01 Mid 0.080 (95%CI 0.024 to 0.189) Estimated Error 0.0422"

[1] "2022-12-06 Mid 0.050 (95%CI 0.017 to 0.108) Estimated Error 0.0237"

[1] "2022-12-29 Mid 0.051 (95%CI 0.016 to 0.113) Estimated Error 0.0251"

"73.2% posterior probability of decrease "

ICU patients

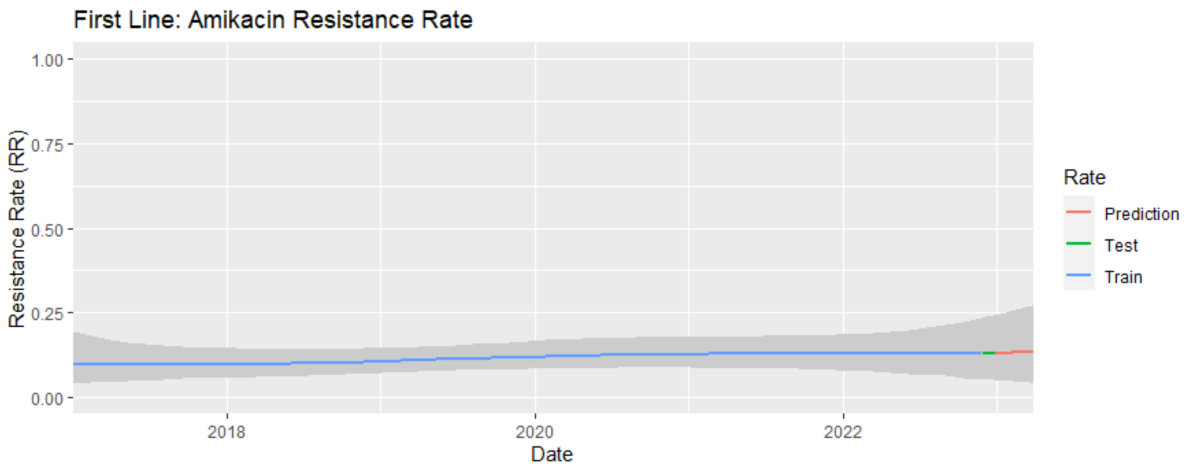

[1] "2017-01-02 Mid 0.099 (95%CI 0.039 to 0.194) Estimated Error 0.0389"

[1] "2022-11-30 Mid 0.129 (95%CI 0.052 to 0.234) Estimated Error 0.0459"

[1] "2022-12-28 Mid 0.130 (95%CI 0.049 to 0.241) Estimated Error 0.0484"

"30.0% posterior probability of decrease "

Haematology/oncology patients

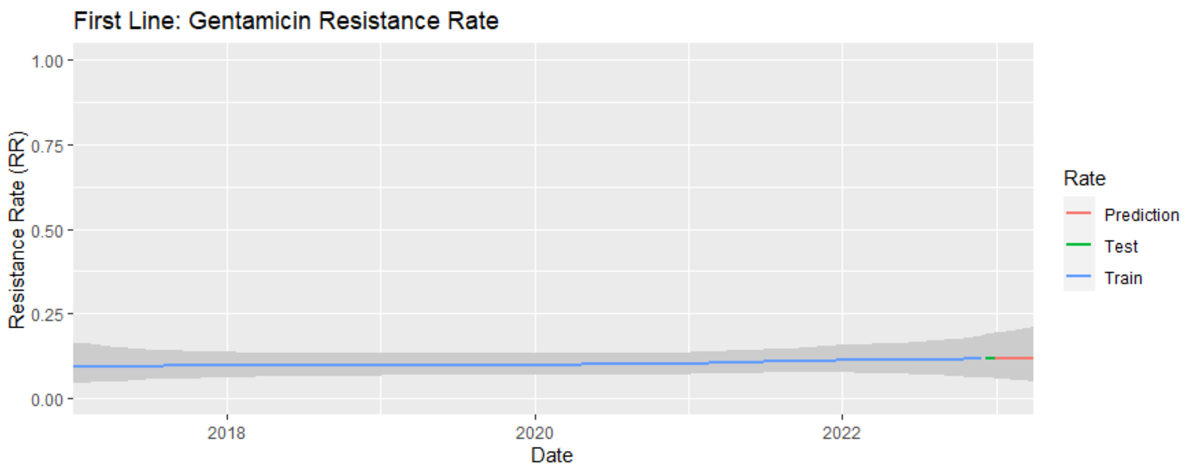

[1] "2017-01-01 Mid 0.094 (95%CI 0.042 to 0.166) Estimated Error 0.0316"

[1] "2022-12-06 Mid 0.115 (95%CI 0.059 to 0.188) Estimated Error 0.0323"

[1] "2022-12-29 Mid 0.115 (95%CI 0.057 to 0.192) Estimated Error 0.0338"

"31.6% posterior probability of decrease "

ICU patients

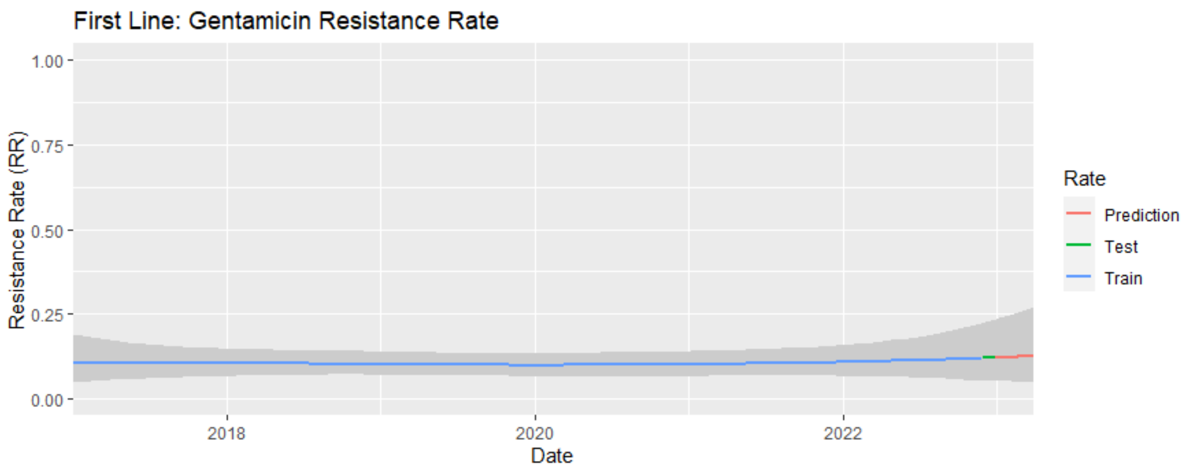

[1] "2017-01-02 Mid 0.106 (95%CI 0.049 to 0.188) Estimated Error 0.0355"

[1] "2022-11-30 Mid 0.119 (95%CI 0.053 to 0.222) Estimated Error 0.0424"

[1] "2022-12-28 Mid 0.120 (95%CI 0.052 to 0.232) Estimated Error 0.0453"

"41.4% posterior probability of decrease "

Haematology/oncology patients

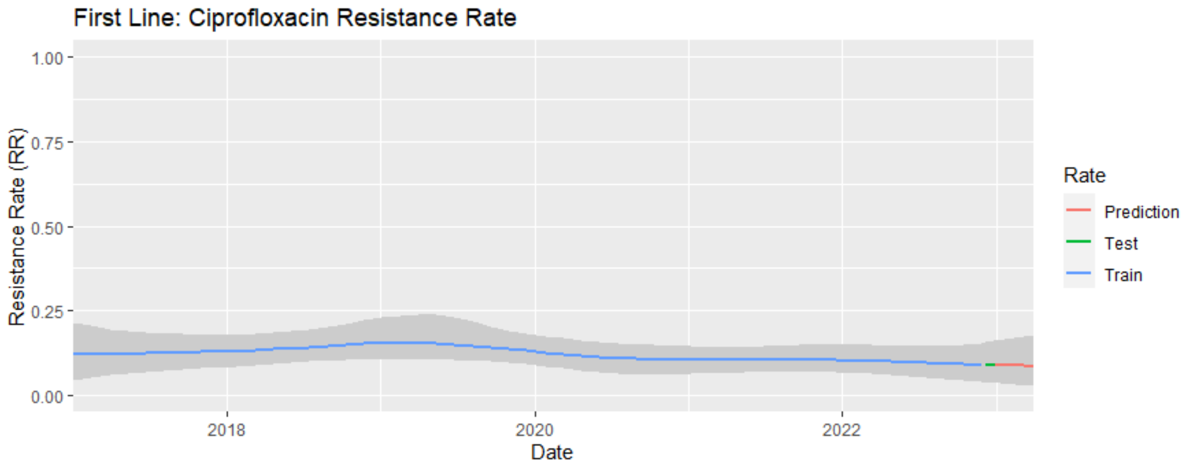

[1] "2017-01-01 Mid 0.121 (95%CI 0.045 to 0.215) Estimated Error 0.0434"

[1] "2022-12-06 Mid 0.089 (95%CI 0.037 to 0.156) Estimated Error 0.0306"

[1] "2022-12-29 Mid 0.088 (95%CI 0.035 to 0.160) Estimated Error 0.0321"

"73.6% posterior probability of decrease "

ICU patients

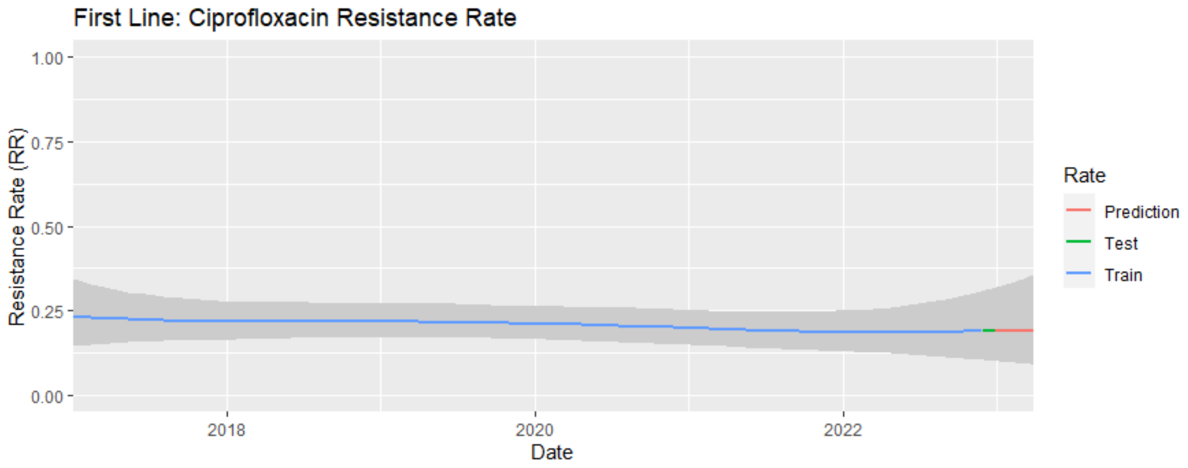

[1] "2017-01-02 Mid 0.234 (95%CI 0.145 to 0.346) Estimated Error 0.0508"

[1] "2022-11-30 Mid 0.188 (95%CI 0.103 to 0.307) Estimated Error 0.0522"

[1] "2022-12-28 Mid 0.189 (95%CI 0.099 to 0.316) Estimated Error 0.0556"

["74.6% posterior probability of decrease "

Haematology/oncology patients

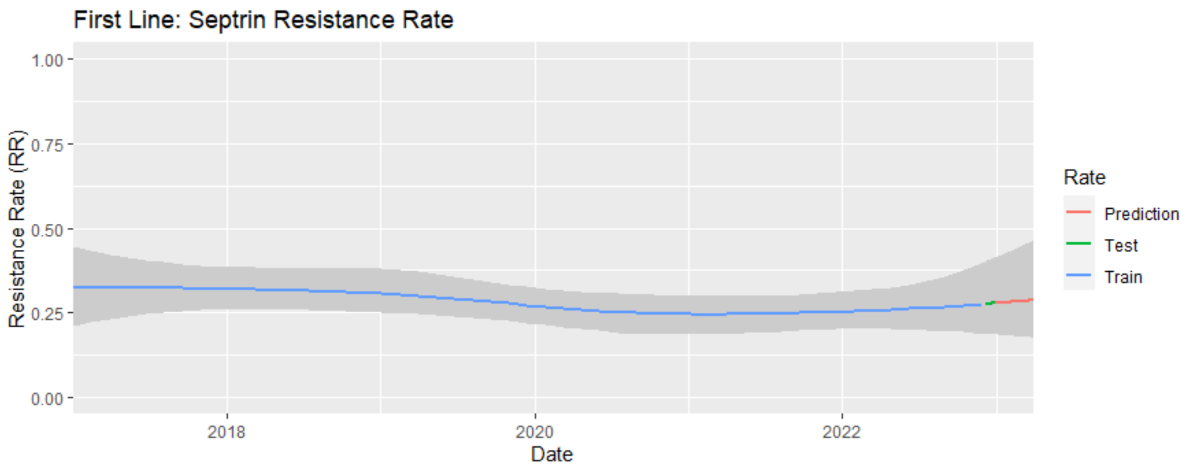

[1] "2017-01-01 Mid 0.324 (95%CI 0.209 to 0.446) Estimated Error 0.0599"

[1] "2022-12-06 Mid 0.276 (95%CI 0.186 to 0.400) Estimated Error 0.0543"

[1] "2022-12-29 Mid 0.278 (95%CI 0.184 to 0.412) Estimated Error 0.0580"

"73.8% posterior probability of decrease "

ICU patients

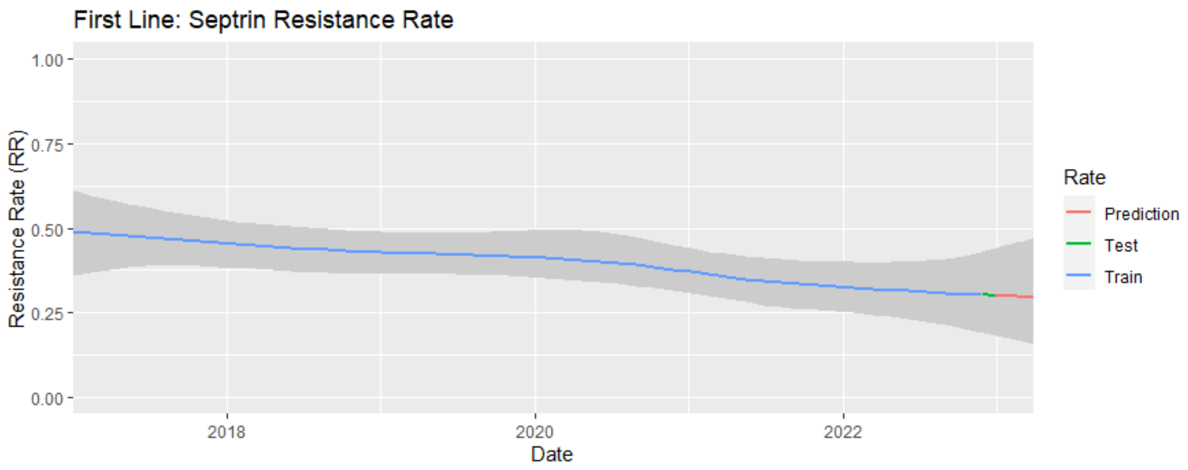

[1] "2017-01-02 Mid 0.487 (95%CI 0.359 to 0.610) Estimated Error 0.0626"

[1] "2022-11-30 Mid 0.302 (95%CI 0.188 to 0.430) Estimated Error 0.0603"

[1] "2022-12-28 Mid 0.300 (95%CI 0.181 to 0.438) Estimated Error 0.0638"

"97.9% posterior probability of decrease "

Haematology/oncology patients

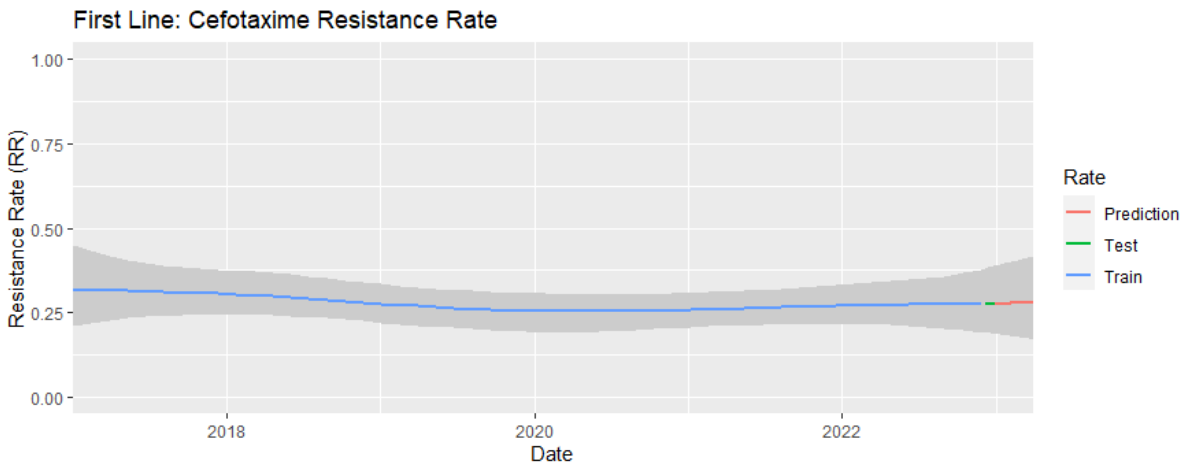

[1] "2017-01-01 Mid 0.319 (95%CI 0.210 to 0.448) Estimated Error 0.0592"

[1] "2022-12-06 Mid 0.276 (95%CI 0.190 to 0.381) Estimated Error 0.0485"

[1] "2022-12-29 Mid 0.276 (95%CI 0.186 to 0.387) Estimated Error 0.0509"

"72.2% posterior probability of decrease "

ICU patients

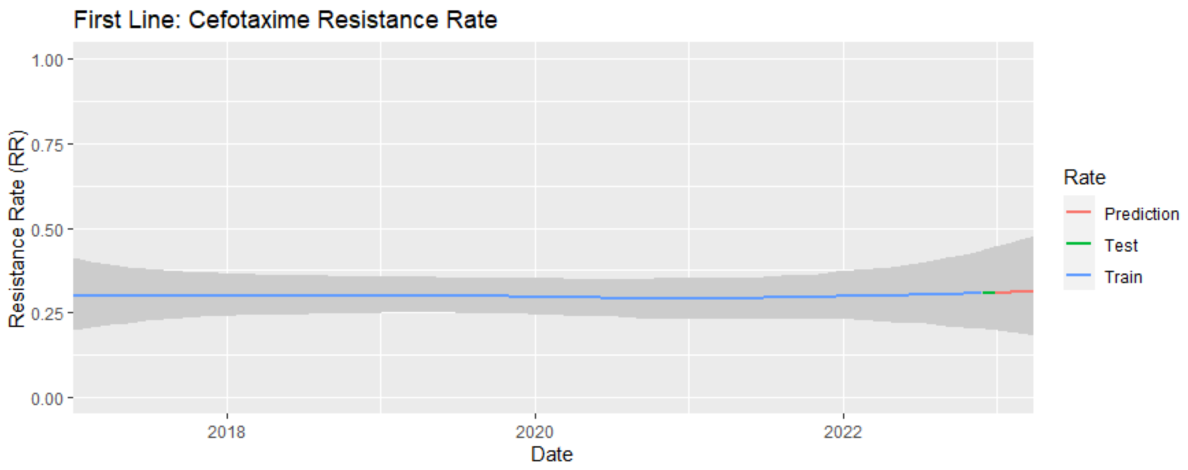

[1] "2017-01-02 Mid 0.298 (95%CI 0.196 to 0.411) Estimated Error 0.0538"

[1] "2022-11-30 Mid 0.307 (95%CI 0.200 to 0.435) Estimated Error 0.0597"

[1] "2022-12-28 Mid 0.308 (95%CI 0.196 to 0.444) Estimated Error 0.0628"

"46.3% posterior probability of decrease "

Haematology/oncology patients

Antibiotic resistance rate over time assuming pip/taz resistance

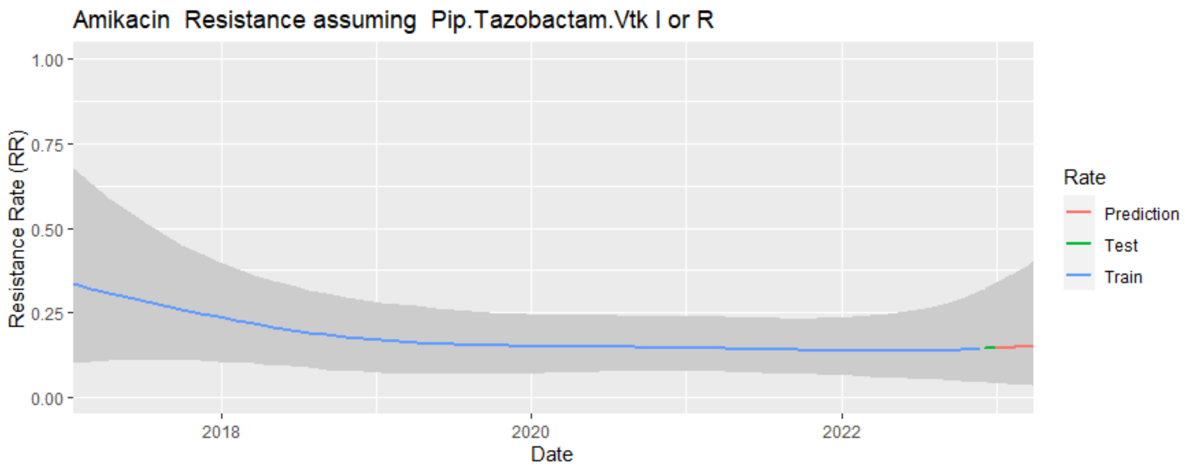

1] "2017-01-16 Mid 0.334 (95%CI 0.101 to 0.678) Estimated Error 0.1503"

[1] "2022-12-06 Mid 0.143 (95%CI 0.042 to 0.321) Estimated Error 0.0717"

[1] "2022-12-29 Mid 0.144 (95%CI 0.040 to 0.335) Estimated Error 0.0758"

"88.6% posterior probability of decrease "

ICU patients

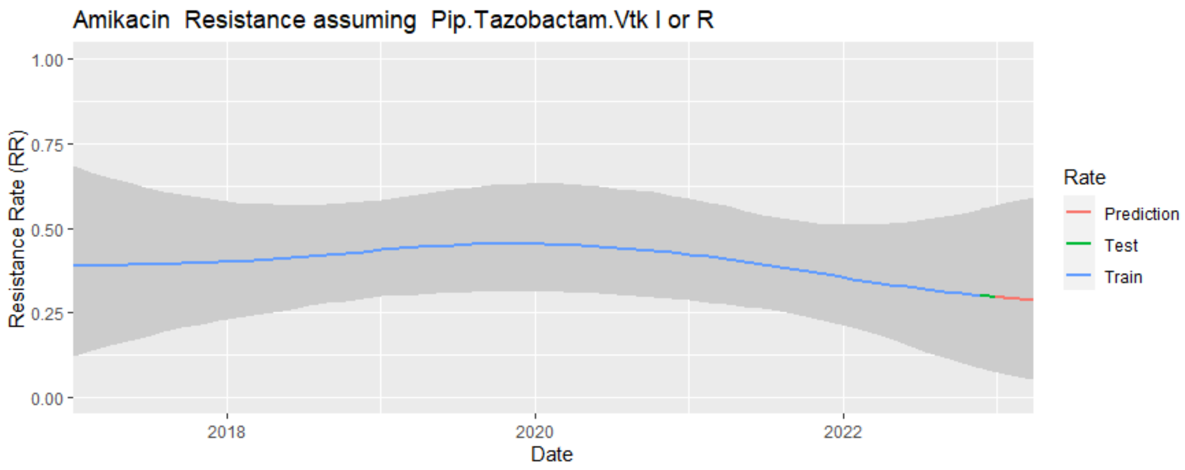

[1] "2017-01-02 Mid 0.389 (95%CI 0.121 to 0.685) Estimated Error 0.1475"

[1] "2022-11-23 Mid 0.300 (95%CI 0.084 to 0.556) Estimated Error 0.1216"

[1] "2022-12-28 Mid 0.296 (95%CI 0.072 to 0.565) Estimated Error 0.1276"

"68.1% posterior probability of decrease "

Haematology/oncology patients

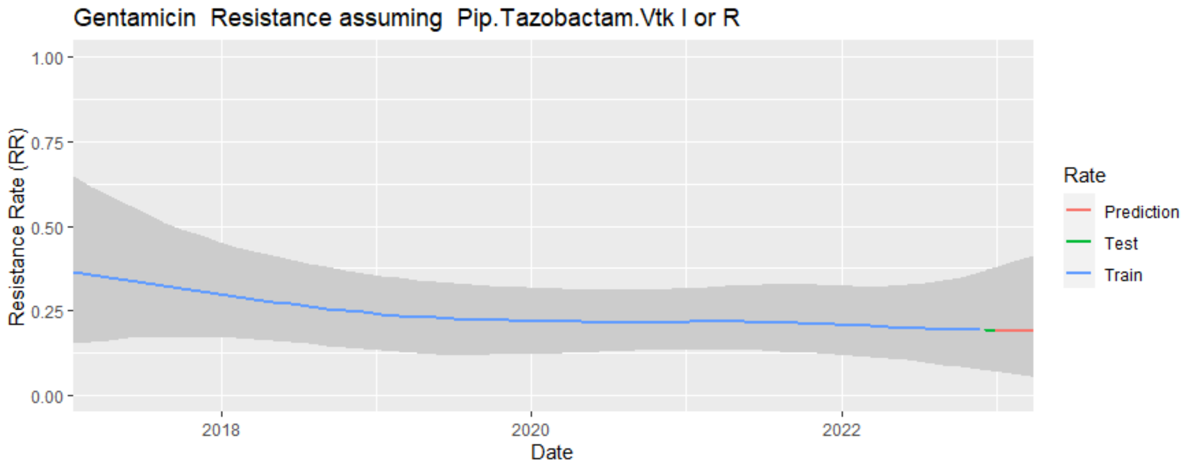

[1] "2017-01-16 Mid 0.366 (95%CI 0.153 to 0.649) Estimated Error 0.1258"

[1] "2022-12-06 Mid 0.192 (95%CI 0.072 to 0.368) Estimated Error 0.0757"

[1] "2022-12-29 Mid 0.191 (95%CI 0.068 to 0.377) Estimated Error 0.0787"

"88.5% posterior probability of decrease "

ICU patients

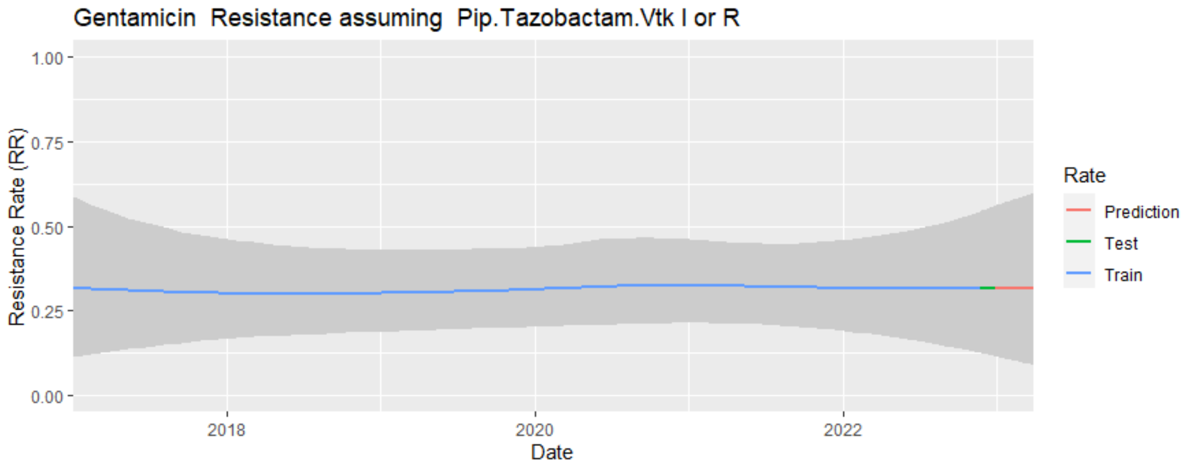

[1] "2017-01-02 Mid 0.318 (95%CI 0.113 to 0.588) Estimated Error 0.1225"

[1] "2022-11-23 Mid 0.314 (95%CI 0.123 to 0.544) Estimated Error 0.1098"

[1] "2022-12-28 Mid 0.315 (95%CI 0.114 to 0.559) Estimated Error 0.1161"

"49.6% posterior probability of decrease "

Haematology/oncology patients

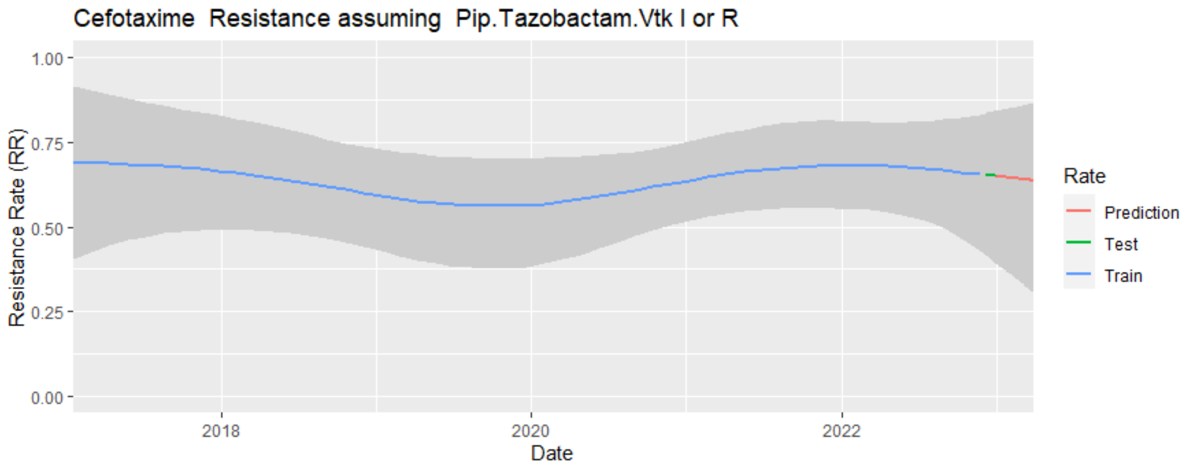

[1] "2017-01-16 Mid 0.690 (95%CI 0.404 to 0.915) Estimated Error 0.1333"

[1] "2022-12-06 Mid 0.654 (95%CI 0.417 to 0.836) Estimated Error 0.1062"

[1] "2022-12-29 Mid 0.650 (95%CI 0.393 to 0.842) Estimated Error 0.1130"

"58.0% posterior probability of decrease "

ICU patients

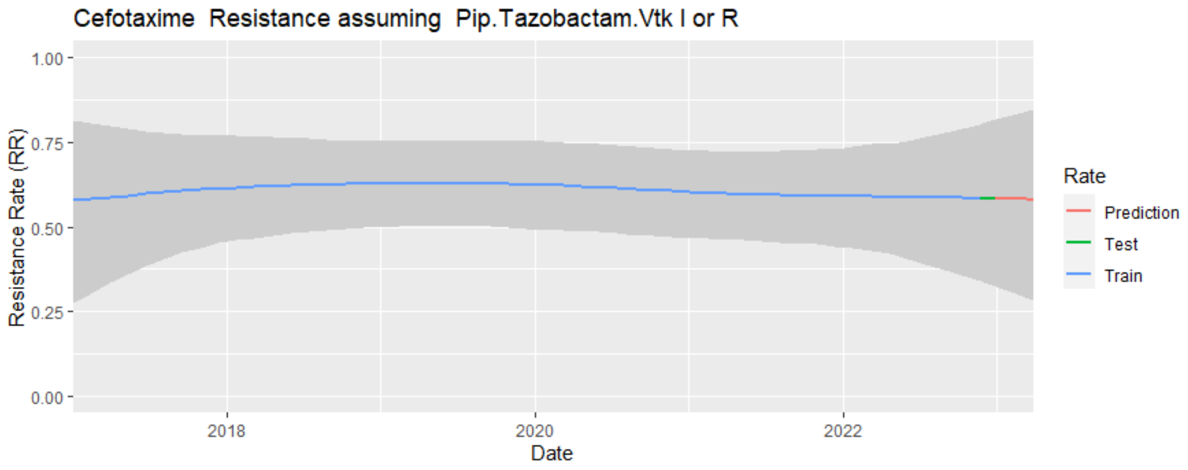

[1] "2017-01-02 Mid 0.579 (95%CI 0.275 to 0.815) Estimated Error 0.1387"

[1] "2022-11-23 Mid 0.584 (95%CI 0.339 to 0.804) Estimated Error 0.1186"

[1] "2022-12-28 Mid 0.584 (95%CI 0.323 to 0.816) Estimated Error 0.1254"

"50.4% posterior probability of decrease "

Haematology/oncology patients

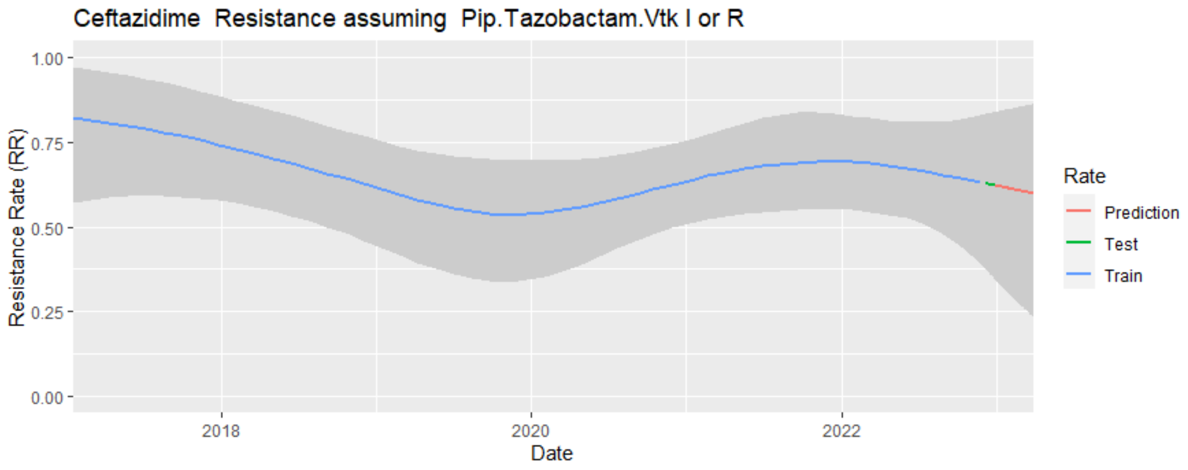

[1] "2017-01-16 Mid 0.822 (95%CI 0.572 to 0.974) Estimated Error 0.1083"

[1] "2022-12-06 Mid 0.628 (95%CI 0.375 to 0.833) Estimated Error 0.1175"

[1] "2022-12-29 Mid 0.622 (95%CI 0.345 to 0.839) Estimated Error 0.1264"

"88.8% posterior probability of decrease "

ICU patients

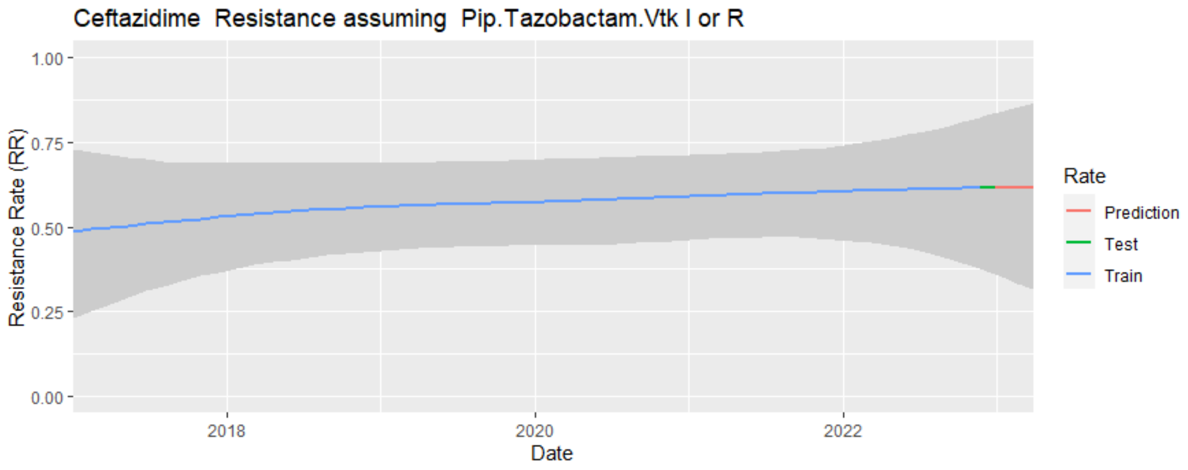

"2017-01-02 Mid 0.487 (95%CI 0.231 to 0.730) Estimated Error 0.1277"

[1] "2022-11-23 Mid 0.615 (95%CI 0.374 to 0.823) Estimated Error 0.1140"

[1] "2022-12-28 Mid 0.616 (95%CI 0.359 to 0.835) Estimated Error 0.1206"

"23.6% posterior probability of decrease "

Haematology/oncology patients

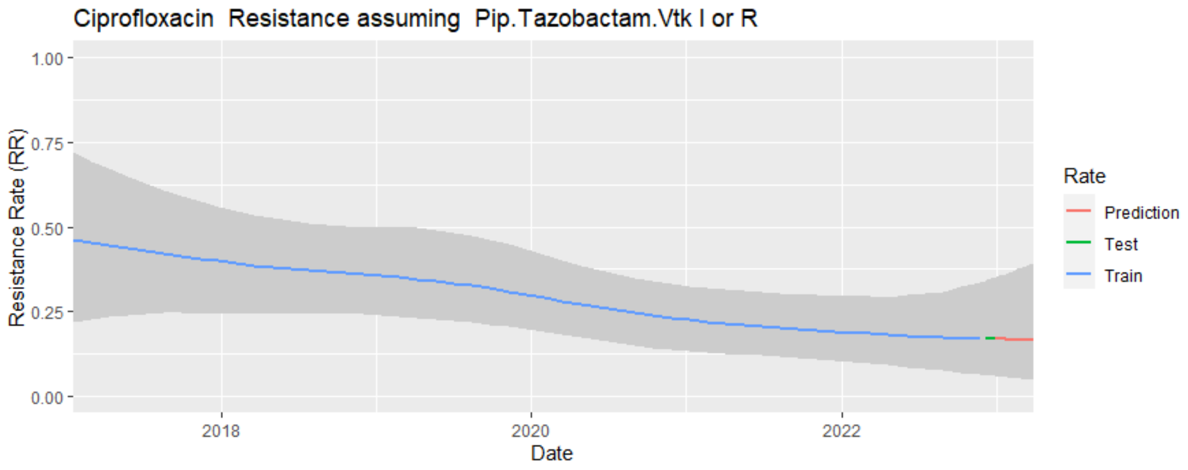

[1] "2017-01-16 Mid 0.463 (95%CI 0.217 to 0.721) Estimated Error 0.1283"

[1] "2022-12-06 Mid 0.169 (95%CI 0.061 to 0.341) Estimated Error 0.0715"

[1] "2022-12-29 Mid 0.168 (95%CI 0.058 to 0.349) Estimated Error 0.0745"

"97.5% posterior probability of decrease "

ICU patients

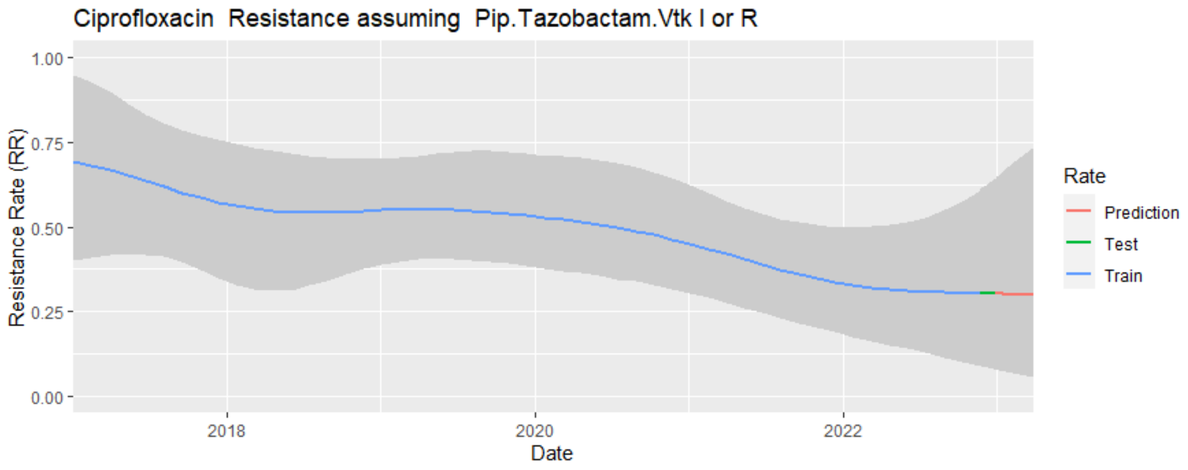

[1] "2017-01-02 Mid 0.694 (95%CI 0.399 to 0.950) Estimated Error 0.1421"

[1] "2022-11-23 Mid 0.302 (95%CI 0.087 to 0.610) Estimated Error 0.1342"

[1] "2022-12-28 Mid 0.302 (95%CI 0.077 to 0.642) Estimated Error 0.1443"

"96.9% posterior probability of decrease "

Haematology/oncology patients

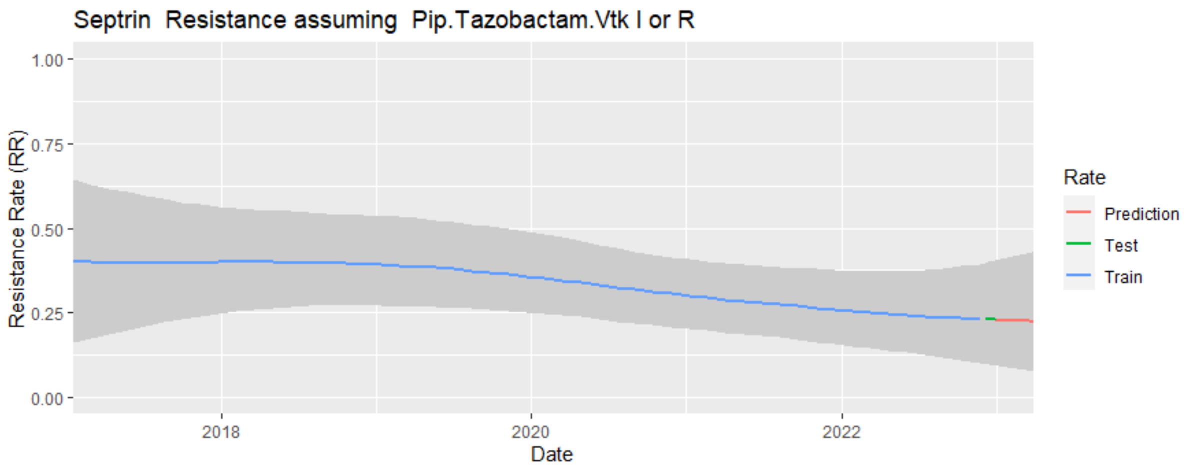

[1] "2017-01-16 Mid 0.399 (95%CI 0.160 to 0.643) Estimated Error 0.1261"

[1] "2022-12-06 Mid 0.229 (95%CI 0.096 to 0.397) Estimated Error 0.0776"

[1] "2022-12-29 Mid 0.228 (95%CI 0.092 to 0.403) Estimated Error 0.0802"

[1] "85.9% posterior probability of decrease "

ICU patients

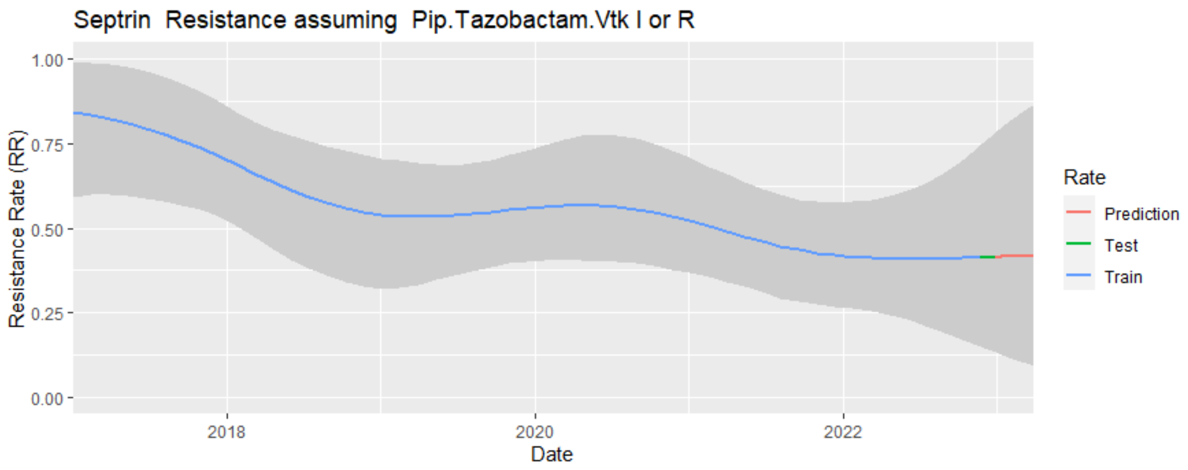

[1] "2017-01-02 Mid 0.845 (95%CI 0.592 to 0.994) Estimated Error 0.1113"

[1] "2022-11-23 Mid 0.413 (95%CI 0.149 to 0.745) Estimated Error 0.1543"

[1] "2022-12-28 Mid 0.414 (95%CI 0.132 to 0.781) Estimated Error 0.1677"

"99.0% posterior probability of decrease "

Haematology/oncology patients

Antibiotic resistance rate over time assuming ceftazidime resistance

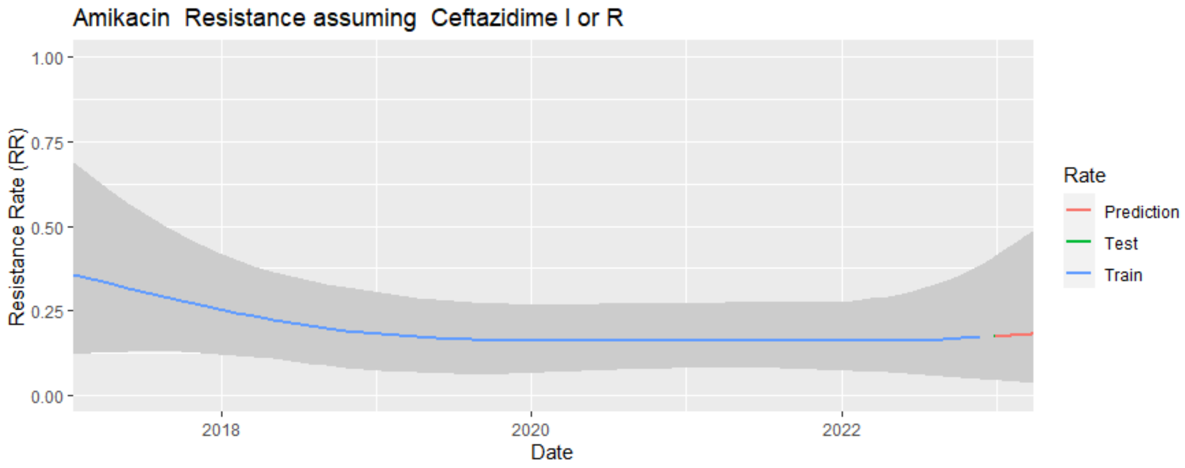

[1] "2017-01-16 Mid 0.355 (95%CI 0.121 to 0.689) Estimated Error 0.1485"

[1] "2022-12-26 Mid 0.172 (95%CI 0.043 to 0.409) Estimated Error 0.0940"

[1] "2022-12-29 Mid 0.172 (95%CI 0.043 to 0.411) Estimated Error 0.0946"

"85.9% posterior probability of decrease "

ICU patients

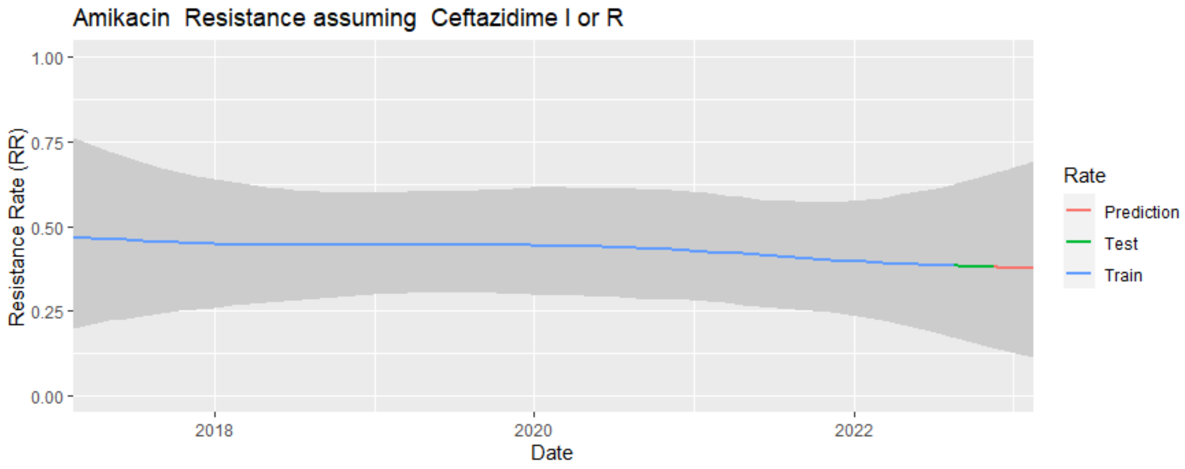

[1] "2017-02-11 Mid 0.470 (95%CI 0.198 to 0.761) Estimated Error 0.1445"

[1] "2022-08-17 Mid 0.383 (95%CI 0.170 to 0.624) Estimated Error 0.1177"

[1] "2022-11-16 Mid 0.379 (95%CI 0.138 to 0.656) Estimated Error 0.1340"

"66.9% posterior probability of decrease "

Haematology/oncology patients

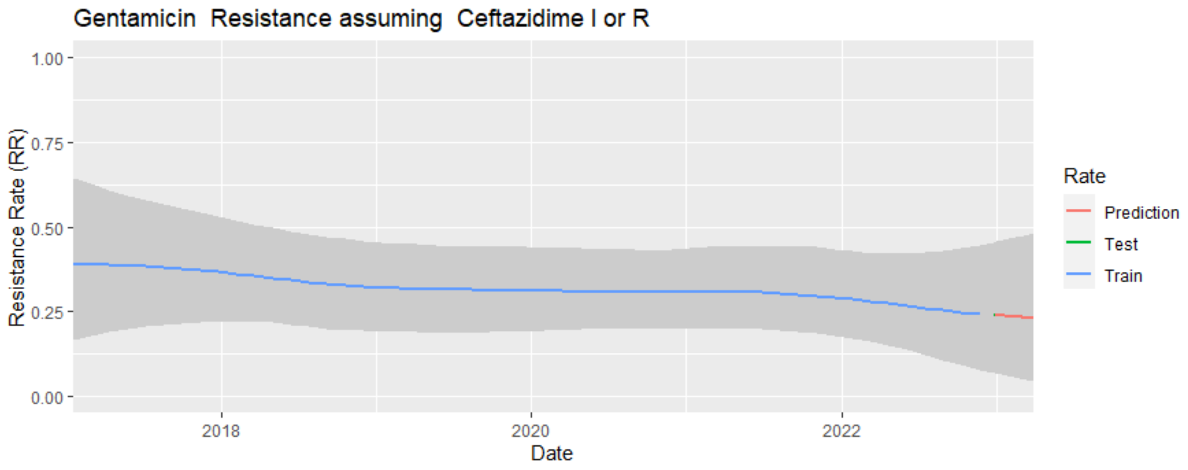

[1] "2017-01-16 Mid 0.392 (95%CI 0.165 to 0.646) Estimated Error 0.1236"

[1] "2022-12-26 Mid 0.239 (95%CI 0.067 to 0.454) Estimated Error 0.0989"

[1] "2022-12-29 Mid 0.239 (95%CI 0.066 to 0.455) Estimated Error 0.0993"

"82.3% posterior probability of decrease "

ICU patients

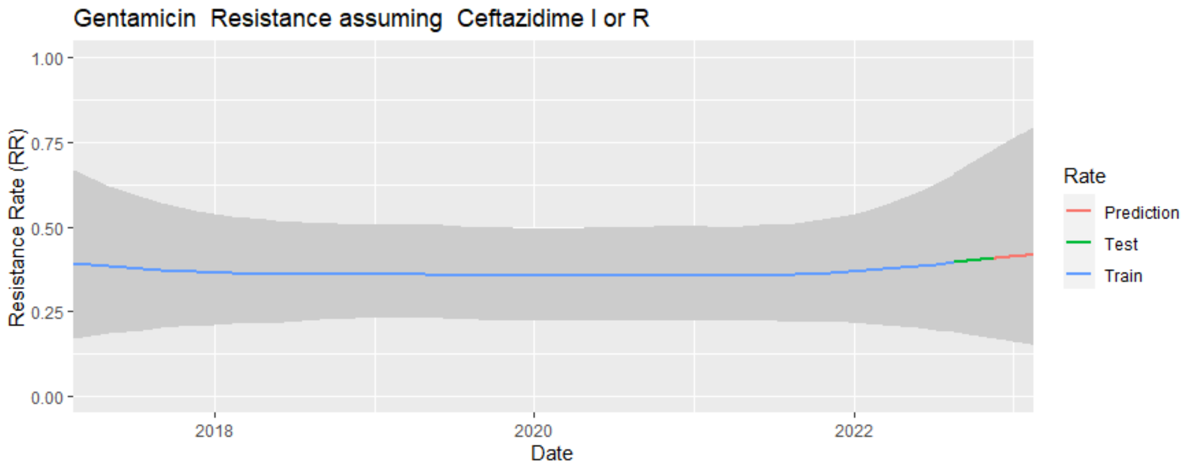

[1] "2017-02-11 Mid 0.393 (95%CI 0.169 to 0.667) Estimated Error 0.1281"

[1] "2022-08-17 Mid 0.395 (95%CI 0.188 to 0.658) Estimated Error 0.1204"

[1] "2022-11-16 Mid 0.407 (95%CI 0.168 to 0.728) Estimated Error 0.1424"

"49.9% posterior probability of decrease "

Haematology/oncology patients

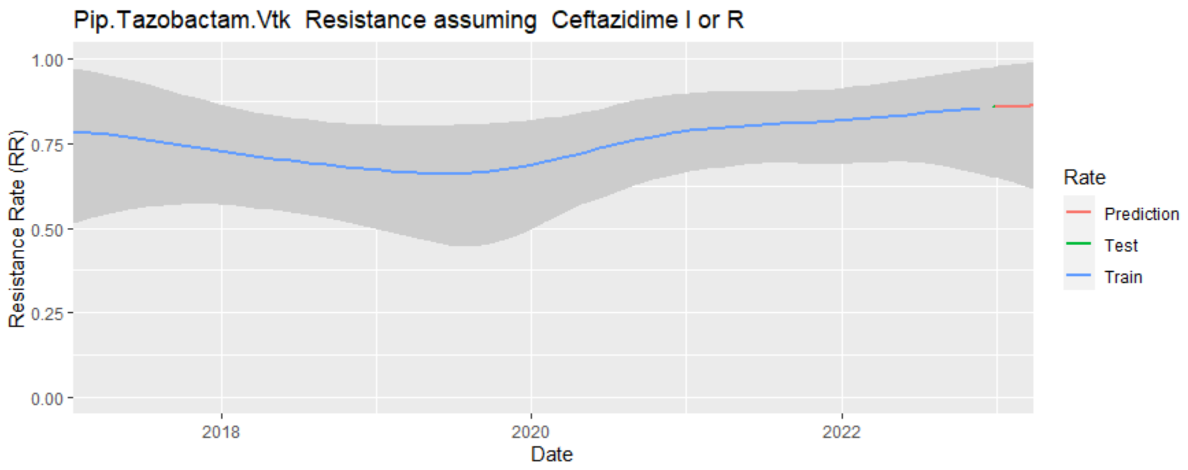

[1] "2017-01-16 Mid 0.787 (95%CI 0.513 to 0.973) Estimated Error 0.1211"

[1] "2022-12-26 Mid 0.857 (95%CI 0.650 to 0.978) Estimated Error 0.0859"

[1] "2022-12-29 Mid 0.858 (95%CI 0.649 to 0.978) Estimated Error 0.0864"

"31.0% posterior probability of decrease "

ICU patients

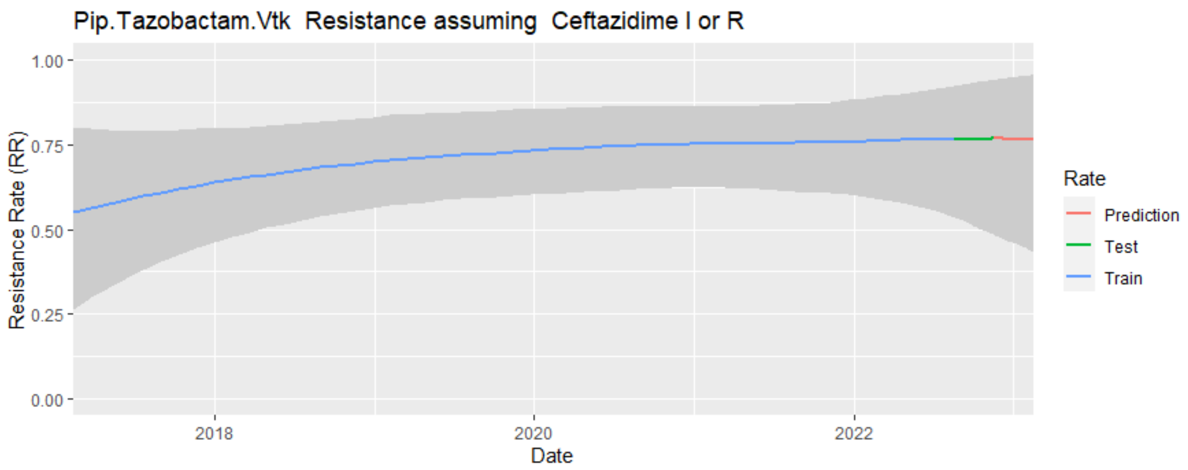

[1] "2017-02-11 Mid 0.552 (95%CI 0.263 to 0.801) Estimated Error 0.1389"

[1] "2022-08-17 Mid 0.768 (95%CI 0.535 to 0.923) Estimated Error 0.1008"

[1] "2022-11-16 Mid 0.768 (95%CI 0.484 to 0.941) Estimated Error 0.1173"

"11.0% posterior probability of decrease "

Haematology/oncology patients

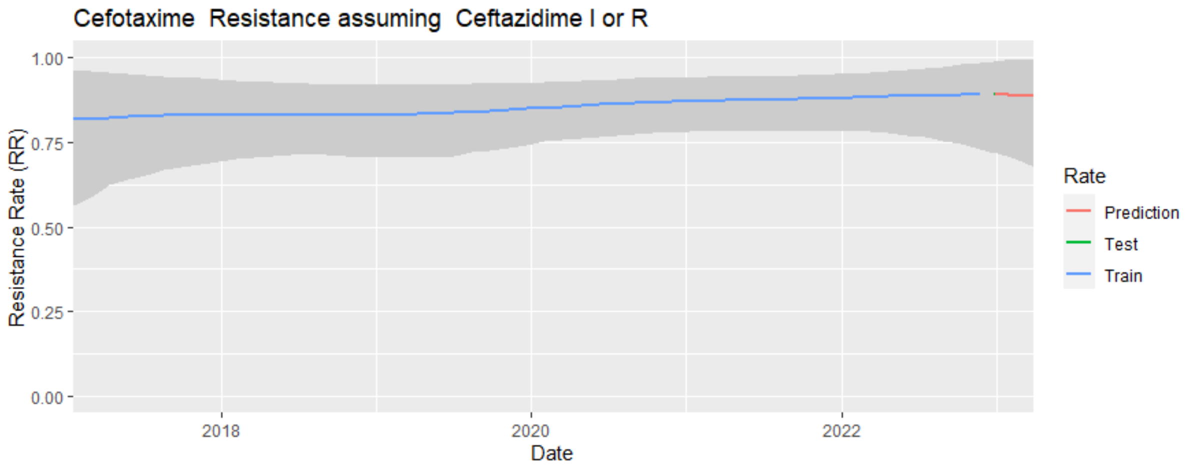

[1] "2017-01-16 Mid 0.817 (95%CI 0.562 to 0.965) Estimated Error 0.1031"

[1] "2022-12-26 Mid 0.890 (95%CI 0.718 to 0.990) Estimated Error 0.0692"

[1] "2022-12-29 Mid 0.890 (95%CI 0.717 to 0.990) Estimated Error 0.0696"

"28.9% posterior probability of decrease "

ICU patients

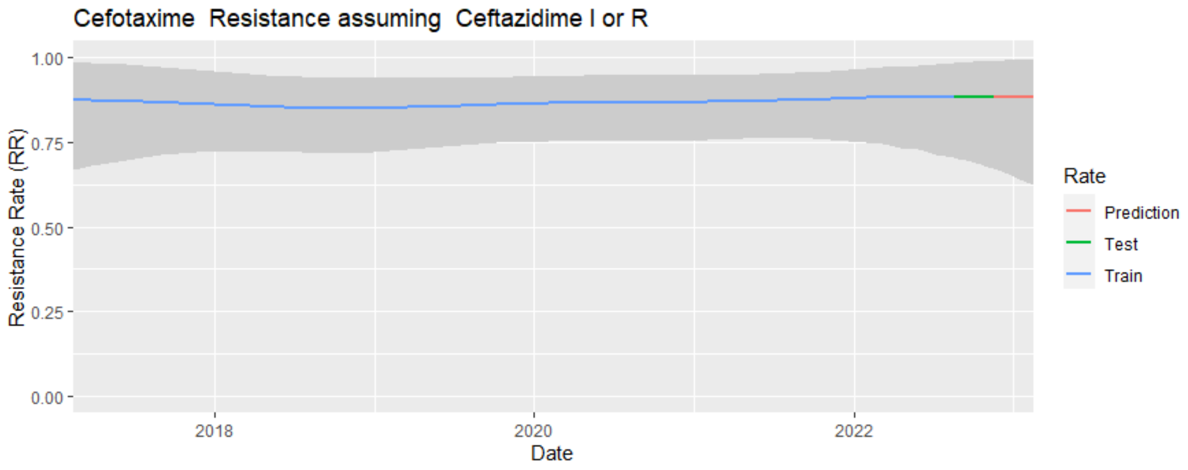

[1] "2017-02-11 Mid 0.875 (95%CI 0.670 to 0.989) Estimated Error 0.0844"

[1] "2022-08-17 Mid 0.885 (95%CI 0.704 to 0.985) Estimated Error 0.0736"

[1] "2022-11-16 Mid 0.884 (95%CI 0.671 to 0.990) Estimated Error 0.0851"

"47.4% posterior probability of decrease "

Haematology/oncology patients

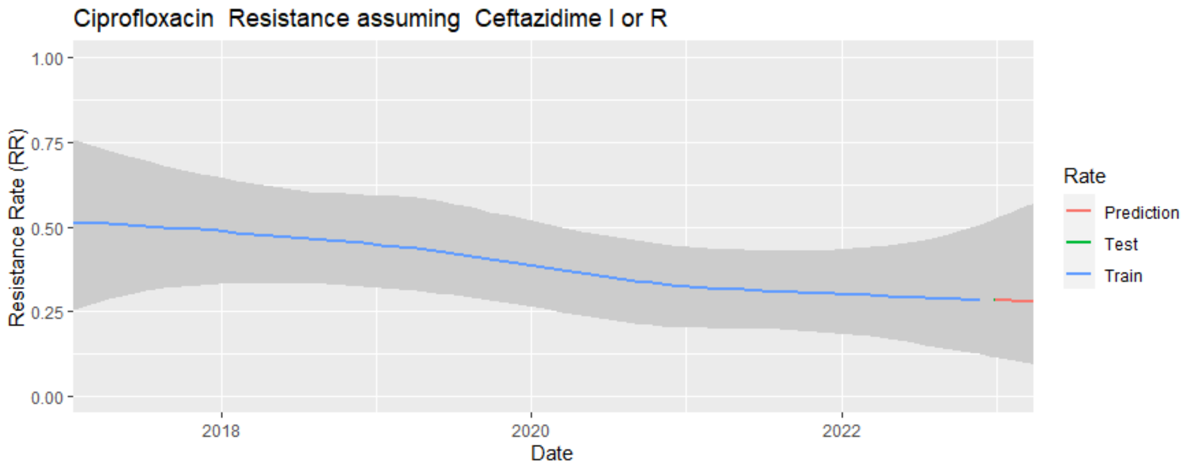

[1] "2017-01-16 Mid 0.515 (95%CI 0.254 to 0.757) Estimated Error 0.1281"

[1] "2022-12-26 Mid 0.282 (95%CI 0.114 to 0.522) Estimated Error 0.1025"

[1] "2022-12-29 Mid 0.282 (95%CI 0.113 to 0.523) Estimated Error 0.1030"

"90.8% posterior probability of decrease "
ICU patients

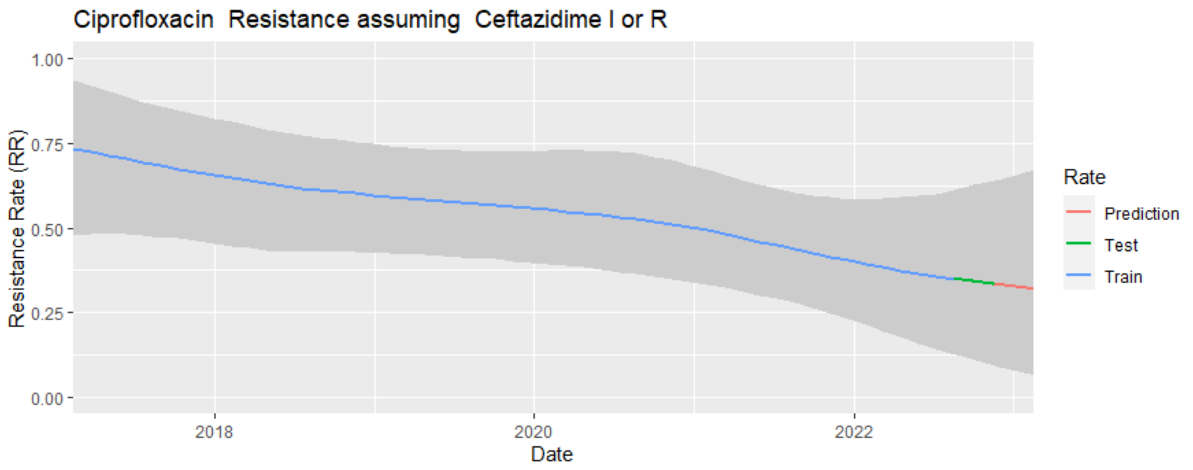

[1] "2017-02-11 Mid 0.733 (95%CI 0.478 to 0.935) Estimated Error 0.1188"

[1] "2022-08-17 Mid 0.350 (95%CI 0.125 to 0.611) Estimated Error 0.1243"

[1] "2022-11-16 Mid 0.333 (95%CI 0.092 to 0.637) Estimated Error 0.1399"

"97.4% posterior probability of decrease "Haematology/oncology patients

Haematology/oncology patients

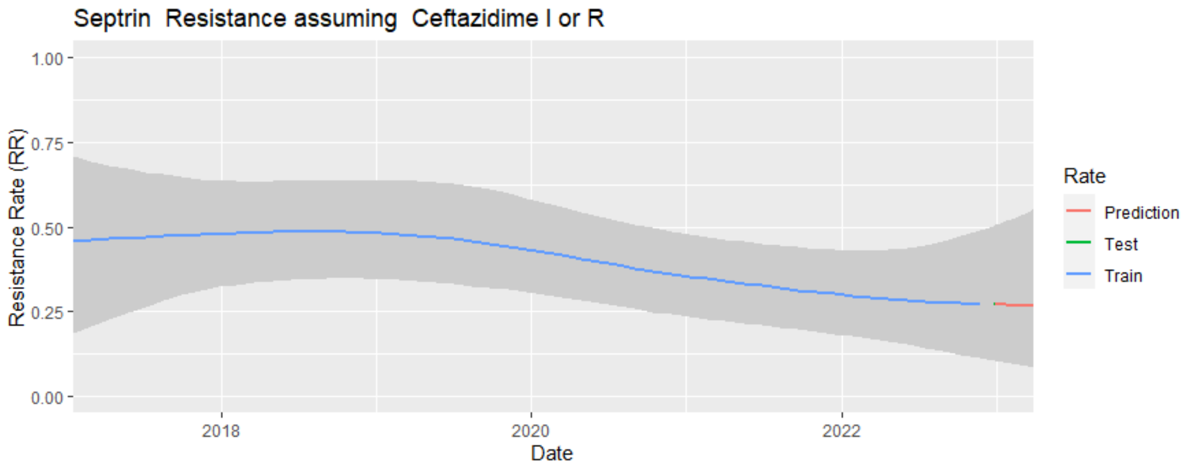

[1] "2017-01-16 Mid 0.459 (95%CI 0.186 to 0.707) Estimated Error 0.1352"

[1] "2022-12-26 Mid 0.270 (95%CI 0.103 to 0.502) Estimated Error 0.1019"

[1] "2022-12-29 Mid 0.270 (95%CI 0.103 to 0.504) Estimated Error 0.1024"

"85.7% posterior probability of decrease "

ICU patients

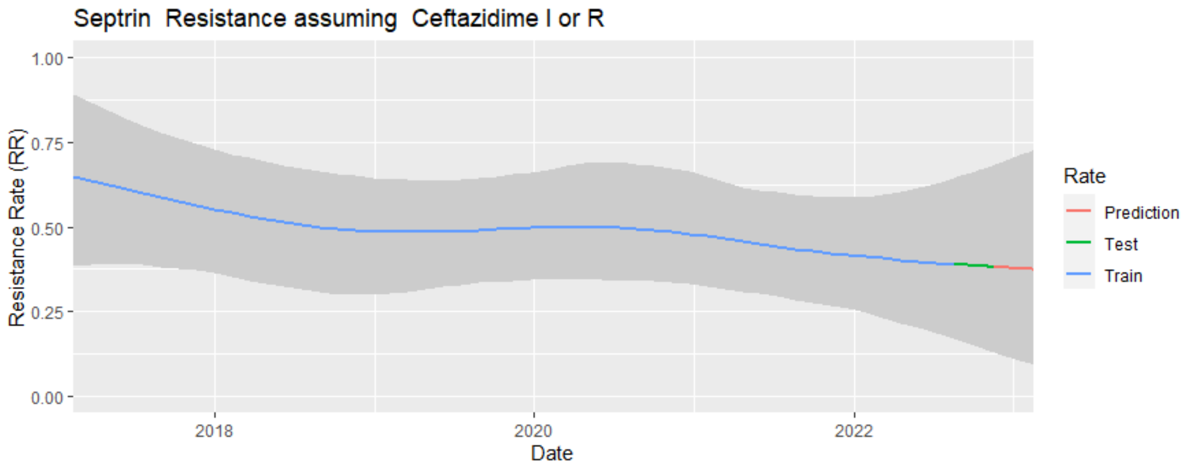

[1] "2017-02-11 Mid 0.648 (95%CI 0.383 to 0.892) Estimated Error 0.1331"

[1] "2022-08-17 Mid 0.390 (95%CI 0.168 to 0.645) Estimated Error 0.1213"

[1] "2022-11-16 Mid 0.381 (95%CI 0.127 to 0.681) Estimated Error 0.1411"

"90.4% posterior probability of decrease "

Haematology/oncology patients

Supplementary Material

Antibiotic resistance rate over time Urine/Renal or GI Source

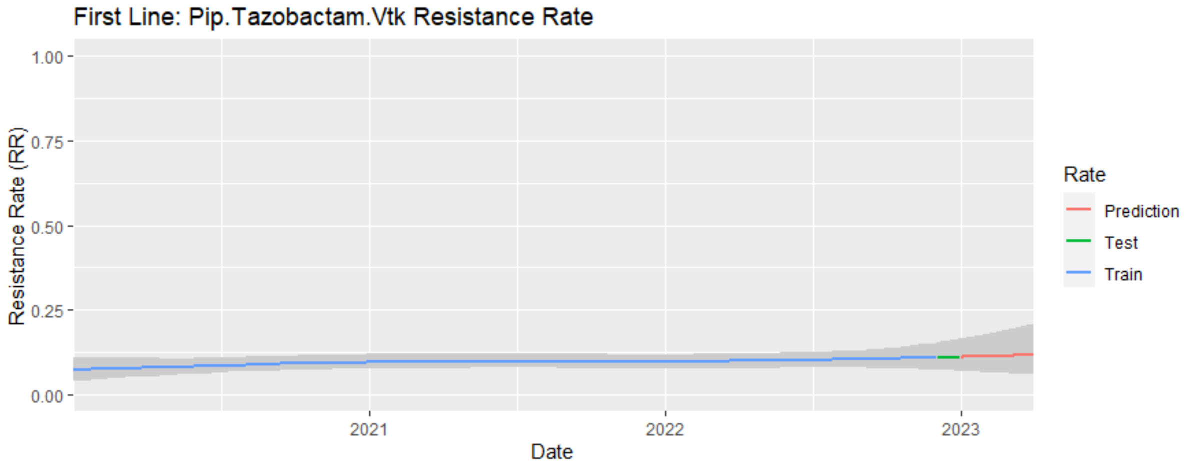

[1] "2020-01-01 Mid 0.073 (95%CI 0.039 to 0.110) Estimated Error 0.0179"

[1] "2022-12-03 Mid 0.109 (95%CI 0.072 to 0.154) Estimated Error 0.0208"

[1] "2022-12-30 Mid 0.110 (95%CI 0.069 to 0.164) Estimated Error 0.0239"

"9.1% posterior probability of decrease "

Urine/renal Source

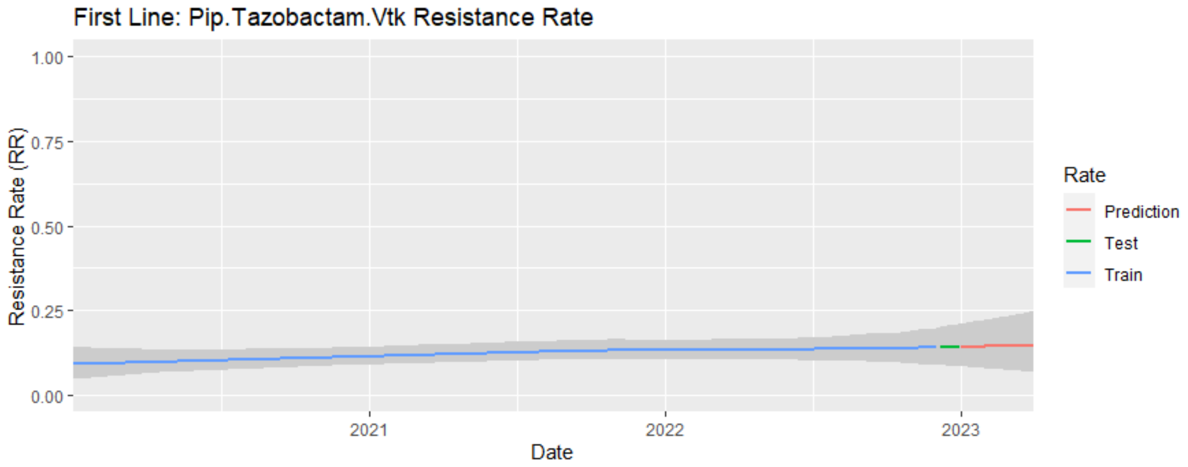

[1] "2020-01-02 Mid 0.092 (95%CI 0.049 to 0.143) Estimated Error 0.0238"

[1] "2022-12-06 Mid 0.140 (95%CI 0.087 to 0.201) Estimated Error 0.0288"

[1] "2022-12-31 Mid 0.141 (95%CI 0.083 to 0.209) Estimated Error 0.0320"

"9.6% posterior probability of decrease "

GI Source

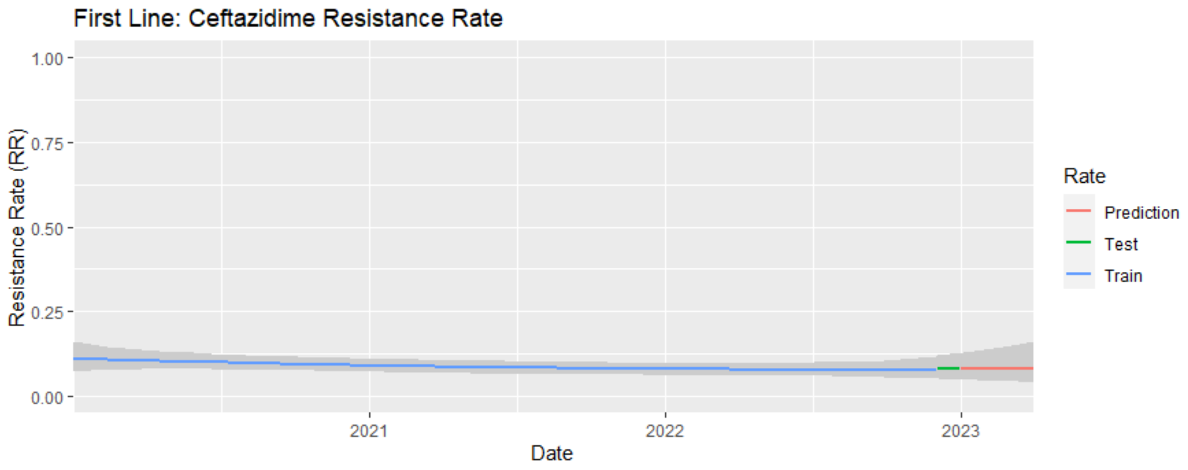

[1] "2020-01-01 Mid 0.111 (95%CI 0.073 to 0.160) Estimated Error 0.0221"

[1] "2022-12-03 Mid 0.078 (95%CI 0.049 to 0.119) Estimated Error 0.0172"

[1] "2022-12-30 Mid 0.078 (95%CI 0.047 to 0.126) Estimated Error 0.0196"

"88.5% posterior probability of decrease "

Urine/renal Source

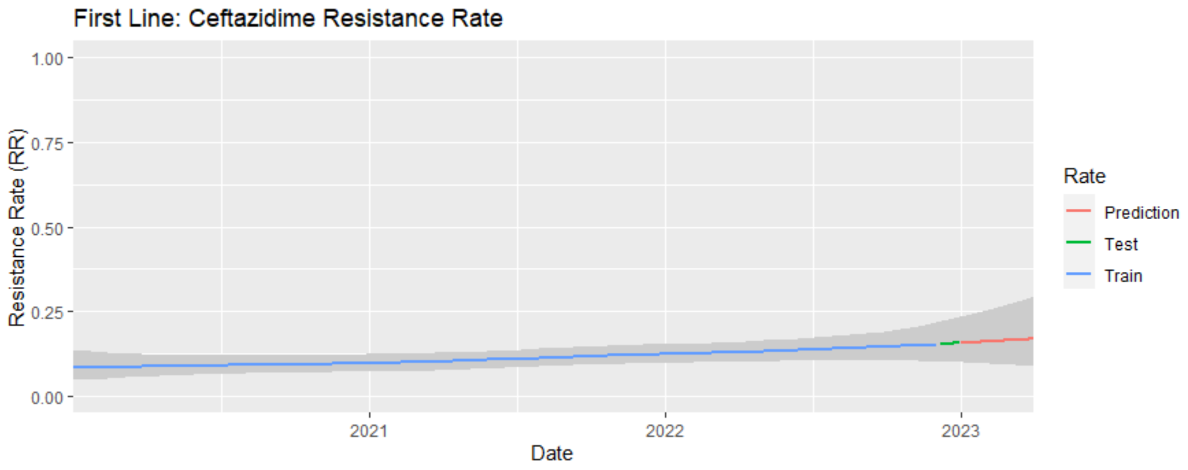

[1] "2020-01-02 Mid 0.083 (95%CI 0.046 to 0.135) Estimated Error 0.0220"

[1] "2022-12-06 Mid 0.153 (95%CI 0.101 to 0.219) Estimated Error 0.0299"

[1] "2022-12-31 Mid 0.156 (95%CI 0.099 to 0.232) Estimated Error 0.0338"

"3.1% posterior probability of decrease "

GI Source

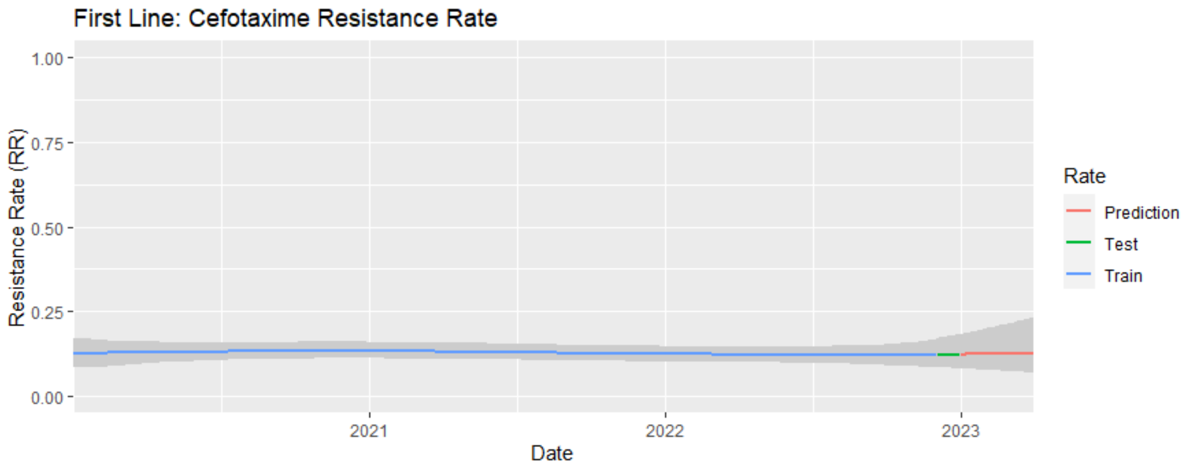

[1] "2020-01-01 Mid 0.126 (95%CI 0.082 to 0.172) Estimated Error 0.0223"

[1] "2022-12-03 Mid 0.122 (95%CI 0.084 to 0.171) Estimated Error 0.0216"

[1] "2022-12-30 Mid 0.122 (95%CI 0.080 to 0.181) Estimated Error 0.0250"

"56.9% posterior probability of decrease "

Urine/renal Source

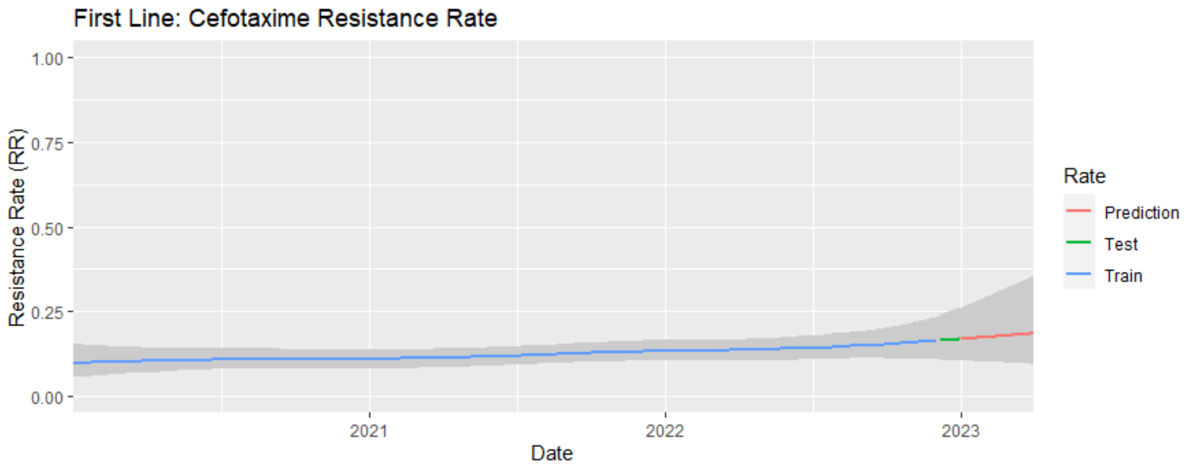

[1] "2020-01-02 Mid 0.098 (95%CI 0.054 to 0.155) Estimated Error 0.0254"

[1] "2022-12-06 Mid 0.164 (95%CI 0.106 to 0.240) Estimated Error 0.0333"

[1] "2022-12-31 Mid 0.168 (95%CI 0.104 to 0.259) Estimated Error 0.0387"

"5.4% posterior probability of decrease "GI Source

GI Source

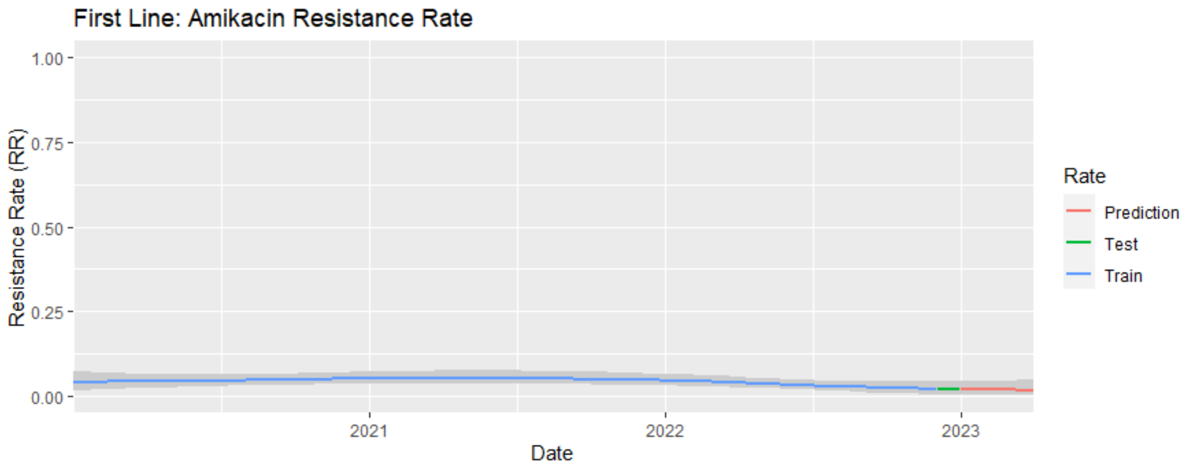

[1] "2020-01-01 Mid 0.041 (95%CI 0.016 to 0.077) Estimated Error 0.0158"

[1] "2022-12-03 Mid 0.020 (95%CI 0.004 to 0.043) Estimated Error 0.0101"

[1] "2022-12-30 Mid 0.019 (95%CI 0.003 to 0.043) Estimated Error 0.0107"

"89.9% posterior probability of decrease "

Urine/renal Source

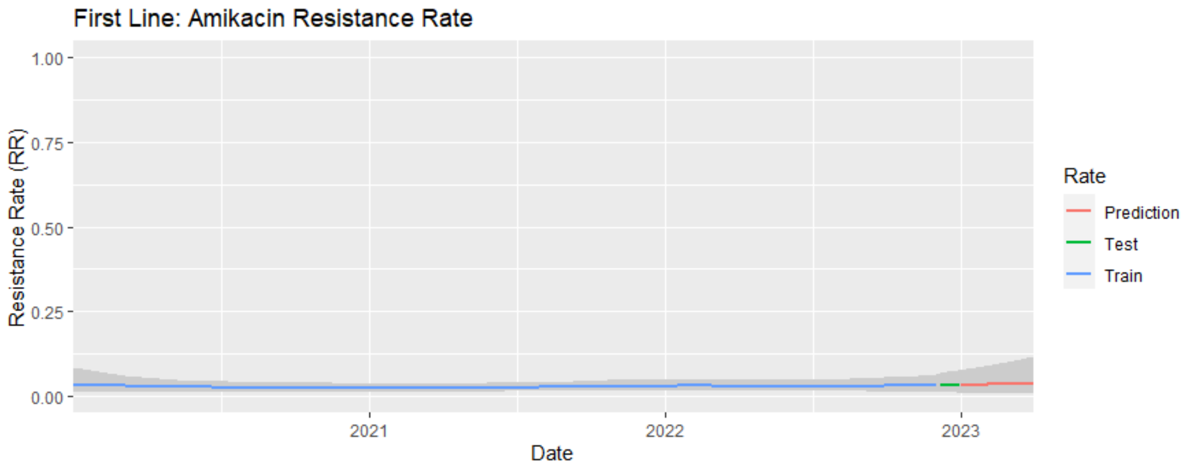

[1] "2020-01-02 Mid 0.034 (95%CI 0.011 to 0.084) Estimated Error 0.0187"

[1] "2022-12-06 Mid 0.031 (95%CI 0.009 to 0.067) Estimated Error 0.0150"

[1] "2022-12-31 Mid 0.032 (95%CI 0.009 to 0.075) Estimated Error 0.0174"

"53.5% posterior probability of decrease "

GI Source

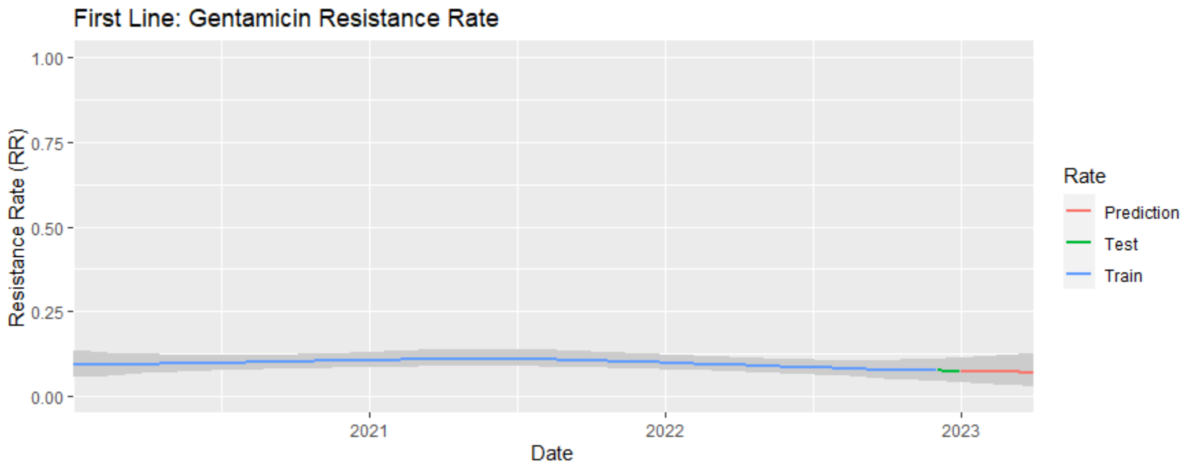

[1] "2020-01-01 Mid 0.093 (95%CI 0.054 to 0.137) Estimated Error 0.0211"

[1] "2022-12-03 Mid 0.074 (95%CI 0.043 to 0.109) Estimated Error 0.0173"

[1] "2022-12-30 Mid 0.073 (95%CI 0.039 to 0.112) Estimated Error 0.0191"

"76.0% posterior probability of decrease "

Urine/renal Source

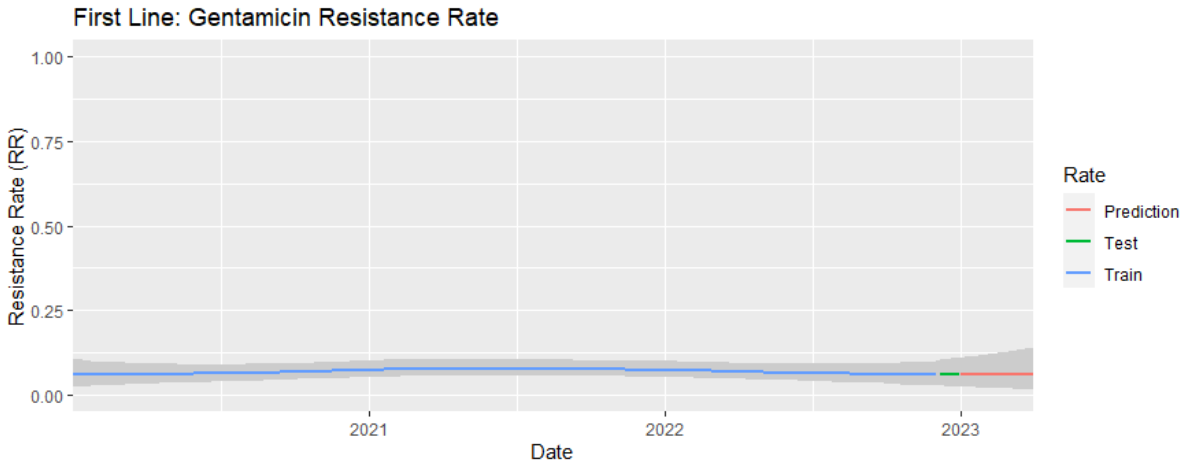

[1] "2020-01-02 Mid 0.059 (95%CI 0.025 to 0.106) Estimated Error 0.0210"

[1] "2022-12-06 Mid 0.059 (95%CI 0.026 to 0.103) Estimated Error 0.0199"

[1] "2022-12-31 Mid 0.059 (95%CI 0.023 to 0.109) Estimated Error 0.0220"

"49.3% posterior probability of decrease "

GI Source

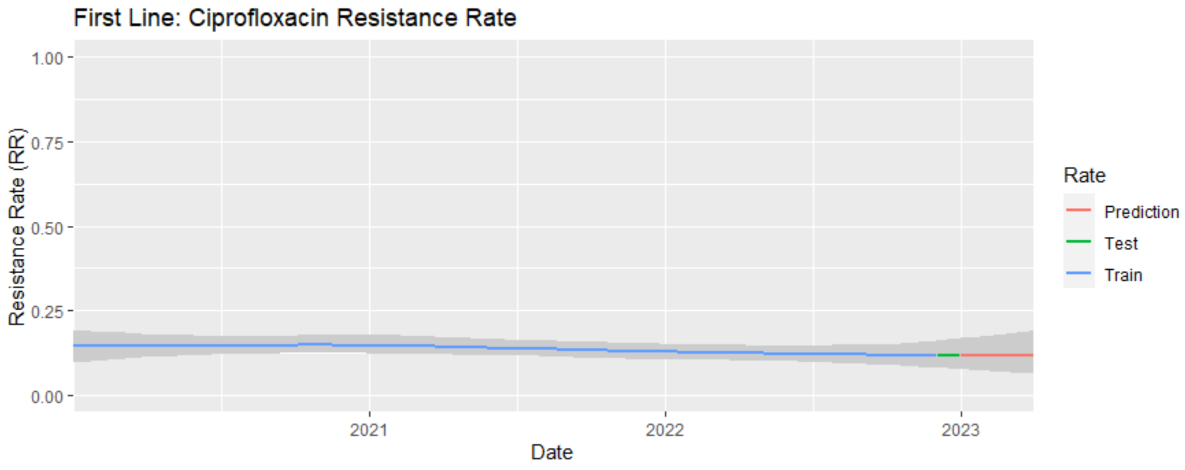

[1] "2020-01-01 Mid 0.145 (95%CI 0.097 to 0.194) Estimated Error 0.0244"

[1] "2022-12-03 Mid 0.116 (95%CI 0.080 to 0.161) Estimated Error 0.0203"

[1] "2022-12-30 Mid 0.116 (95%CI 0.076 to 0.167) Estimated Error 0.0228"

"81.8% posterior probability of decrease "

Urine/renal Source

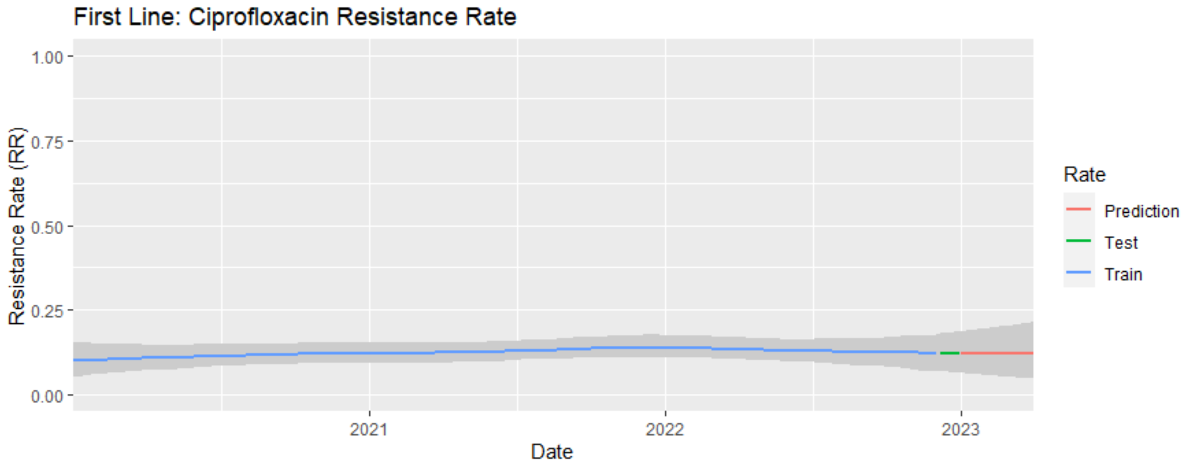

[1] "2020-01-02 Mid 0.101 (95%CI 0.053 to 0.157) Estimated Error 0.0265"

[1] "2022-12-06 Mid 0.122 (95%CI 0.068 to 0.180) Estimated Error 0.0284"

[1] "2022-12-31 Mid 0.122 (95%CI 0.063 to 0.185) Estimated Error 0.0312"

"27.5% posterior probability of decrease "

GI Source

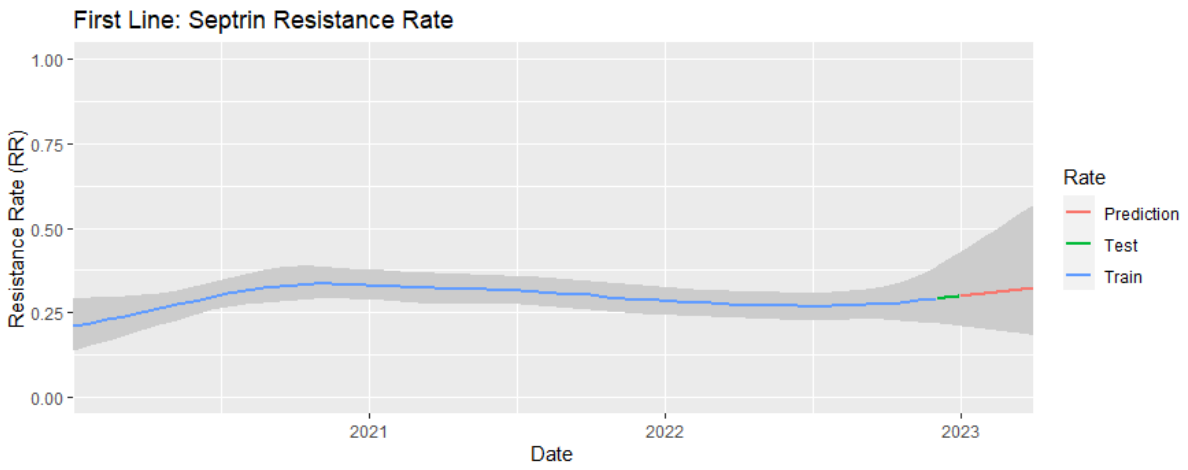

[1] "2020-01-01 Mid 0.210 (95%CI 0.135 to 0.293) Estimated Error 0.0407"

[1] "2022-12-03 Mid 0.291 (95%CI 0.217 to 0.389) Estimated Error 0.0432"

[1] "2022-12-30 Mid 0.298 (95%CI 0.209 to 0.426) Estimated Error 0.0539"

"9.4% posterior probability of decrease "

Urine/renal Source

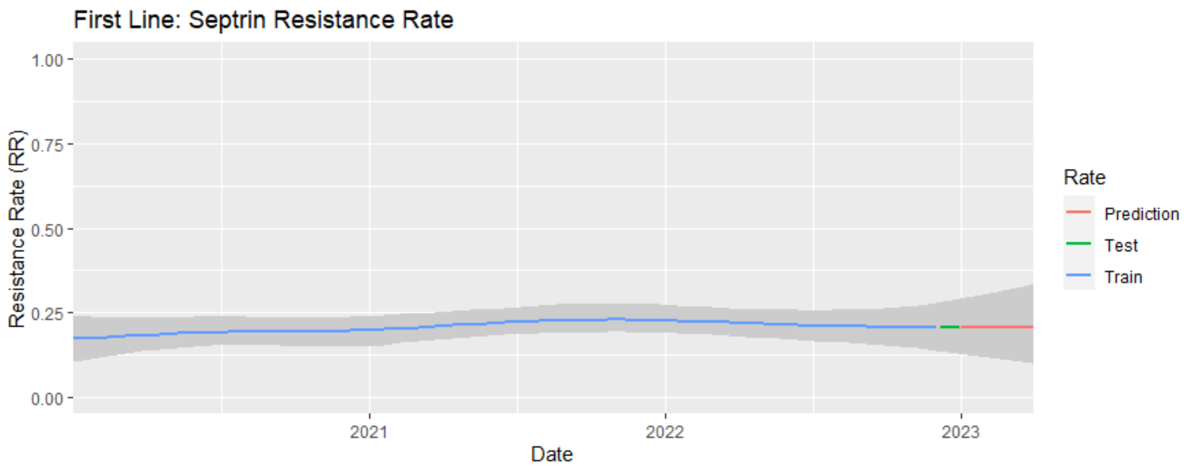

[1] "2020-01-02 Mid 0.172 (95%CI 0.103 to 0.243) Estimated Error 0.0353"

[1] "2022-12-06 Mid 0.206 (95%CI 0.134 to 0.280) Estimated Error 0.0366"

[1] "2022-12-31 Mid 0.205 (95%CI 0.126 to 0.290) Estimated Error 0.0409"

"23.7% posterior probability of decrease "

GI Source

Antibiotic resistance rate over time assuming pip/taz resistance

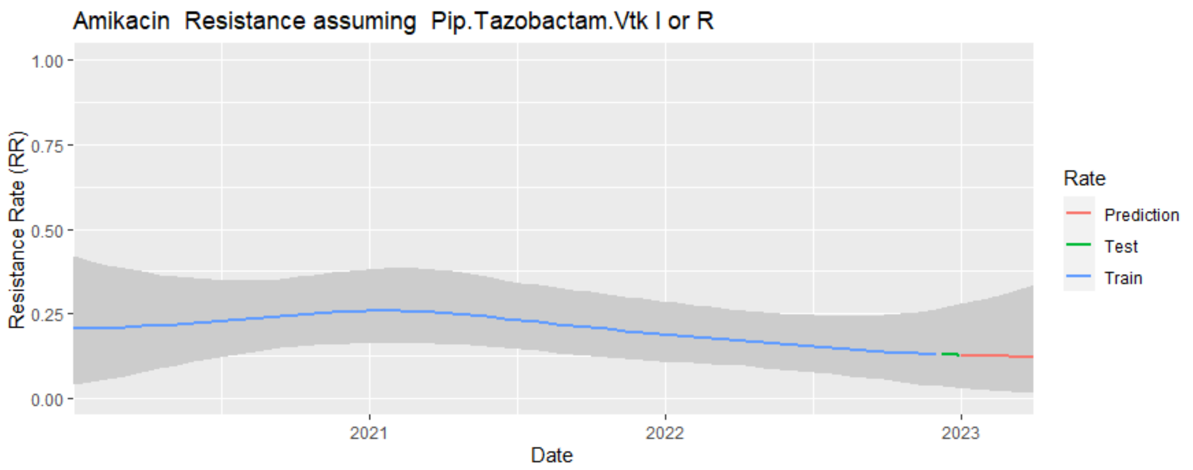

[1] "2020-01-01 Mid 0.205 (95%CI 0.040 to 0.422) Estimated Error 0.1009"

[1] "2022-12-07 Mid 0.128 (95%CI 0.034 to 0.267) Estimated Error 0.0607"

[1] "2022-12-30 Mid 0.126 (95%CI 0.028 to 0.277) Estimated Error 0.0648"

"74.1% posterior probability of decrease "

Urine/renal Source

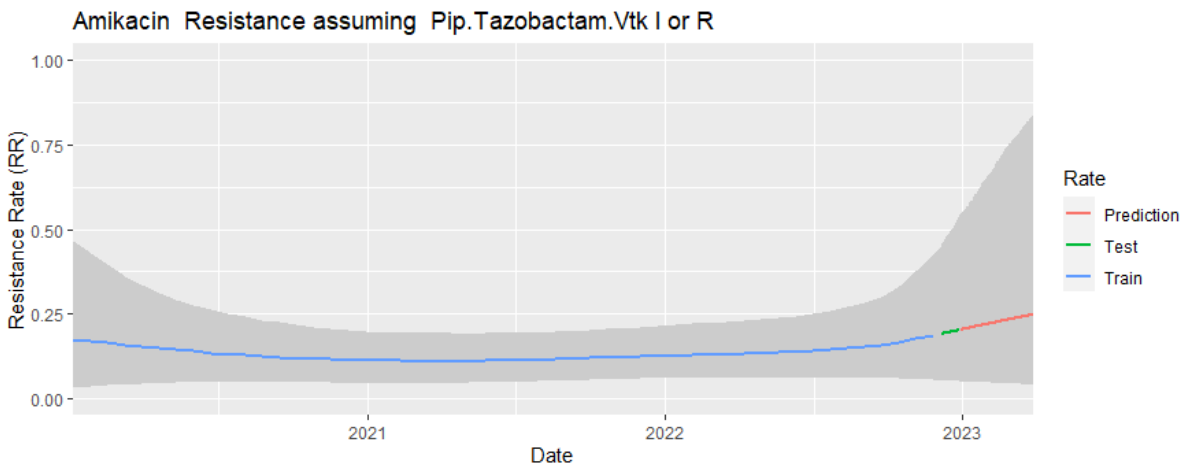

[1] "2020-01-06 Mid 0.175 (95%CI 0.033 to 0.465) Estimated Error 0.1147"

[1] "2022-12-06 Mid 0.191 (95%CI 0.052 to 0.453) Estimated Error 0.1050"

[1] "2022-12-28 Mid 0.202 (95%CI 0.049 to 0.541) Estimated Error 0.1232"

"43.9% posterior probability of decrease "

GI Source

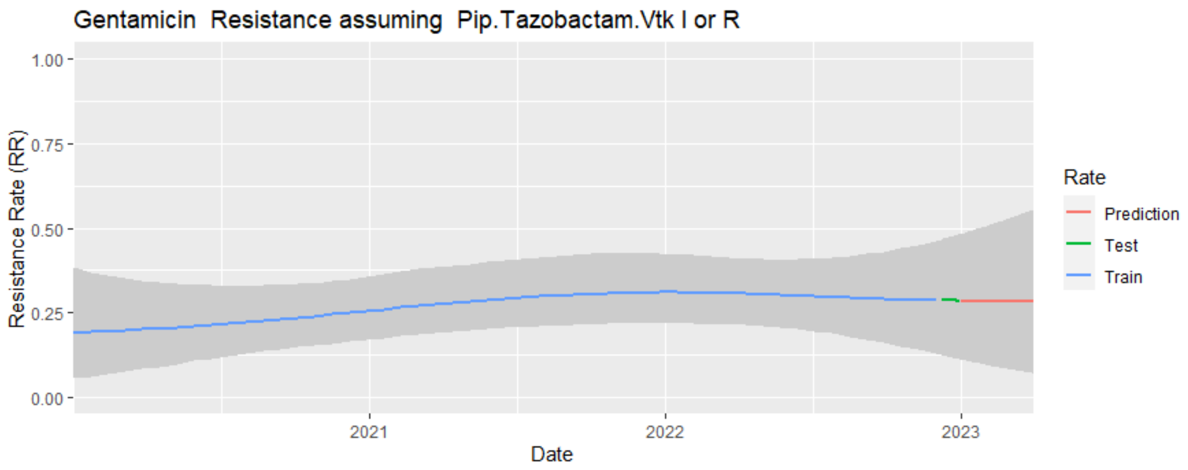

[1] "2020-01-01 Mid 0.191 (95%CI 0.055 to 0.383) Estimated Error 0.0860"

[1] "2022-12-07 Mid 0.286 (95%CI 0.123 to 0.467) Estimated Error 0.0886"

[1] "2022-12-30 Mid 0.285 (95%CI 0.110 to 0.481) Estimated Error 0.0958"

"21.3% posterior probability of decrease "

Urine/renal Source

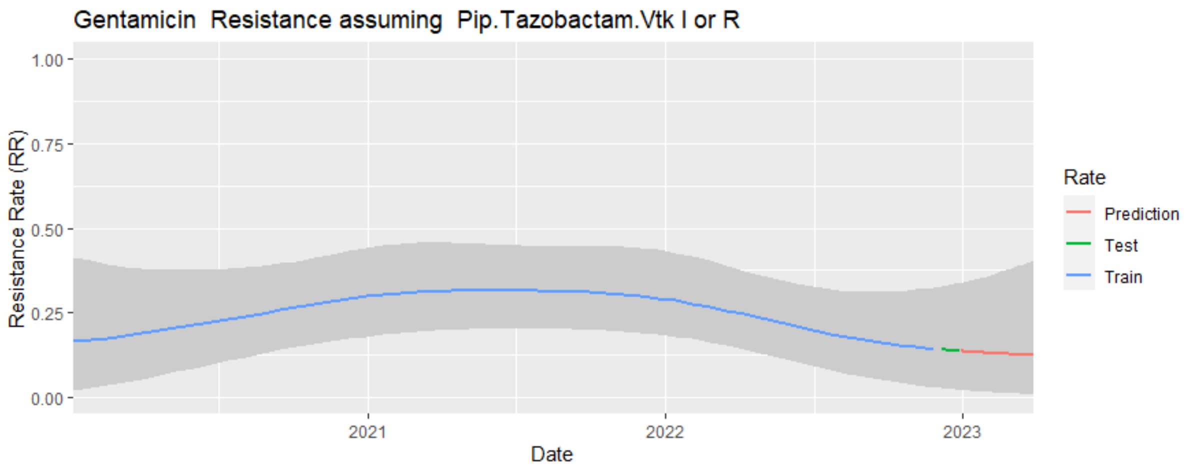

[1] "2020-01-06 Mid 0.164 (95%CI 0.020 to 0.415) Estimated Error 0.1054"

[1] "2022-12-06 Mid 0.140 (95%CI 0.025 to 0.326) Estimated Error 0.0803"

[1] "2022-12-28 Mid 0.135 (95%CI 0.020 to 0.336) Estimated Error 0.0849"

"56.4% posterior probability of decrease "

GI Source

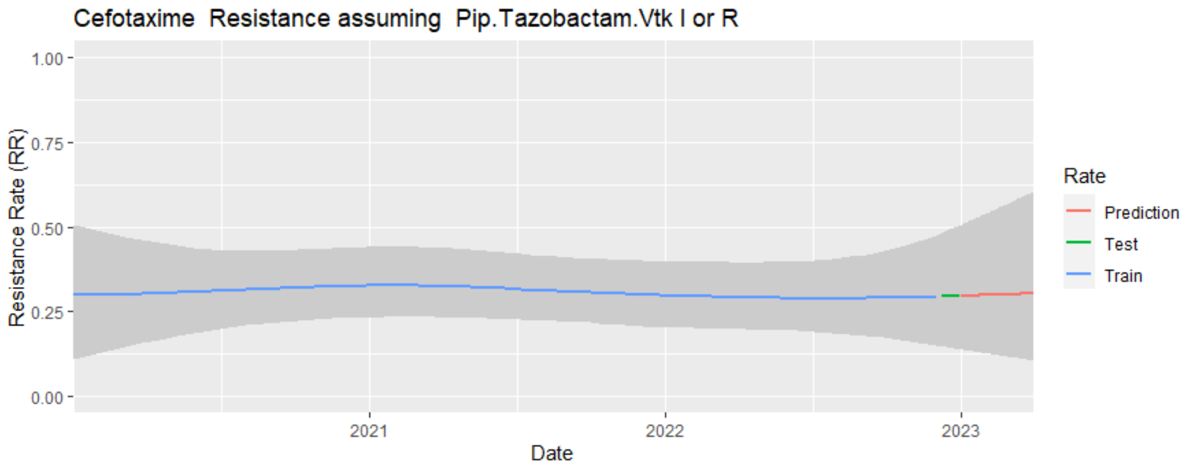

[1] "2020-01-01 Mid 0.298 (95%CI 0.110 to 0.505) Estimated Error 0.1007"

[1] "2022-12-07 Mid 0.294 (95%CI 0.145 to 0.481) Estimated Error 0.0856"

[1] "2022-12-30 Mid 0.295 (95%CI 0.136 to 0.503) Estimated Error 0.0933"

"51.5% posterior probability of decrease "

Urine/renal Source

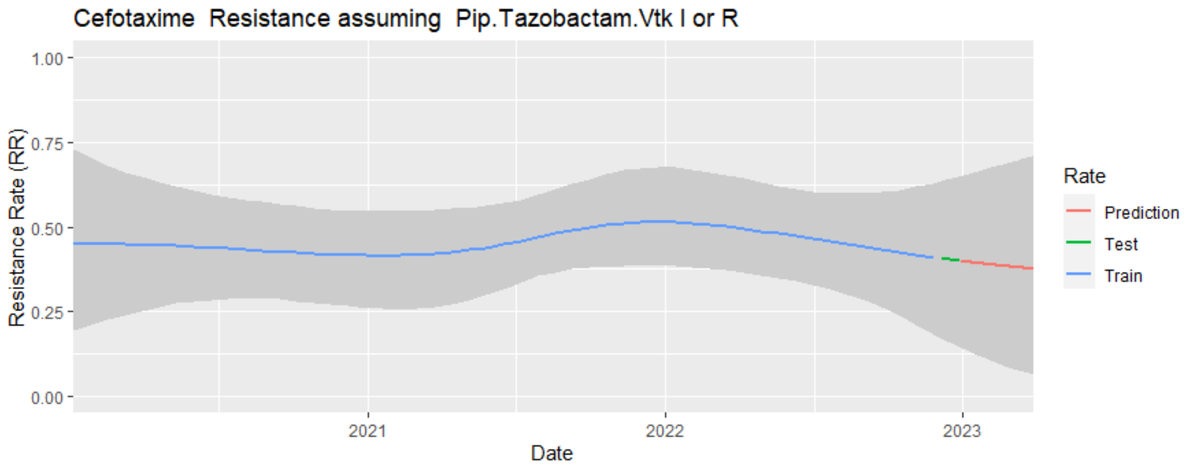

[1] "2020-01-06 Mid 0.450 (95%CI 0.193 to 0.730) Estimated Error 0.1376"

[1] "2022-12-06 Mid 0.406 (95%CI 0.169 to 0.635) Estimated Error 0.1188"

[1] "2022-12-28 Mid 0.399 (95%CI 0.143 to 0.648) Estimated Error 0.1297"

"57.5% posterior probability of decrease "

GI Source

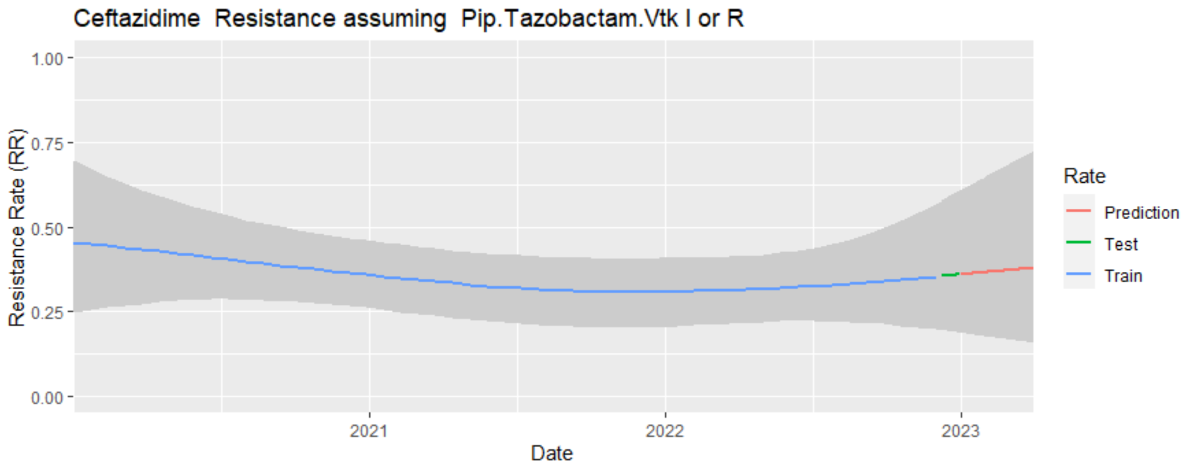

[1] "2020-01-01 Mid 0.454 (95%CI 0.245 to 0.697) Estimated Error 0.1157"

[1] "2022-12-07 Mid 0.354 (95%CI 0.194 to 0.577) Estimated Error 0.0981"

[1] "2022-12-30 Mid 0.359 (95%CI 0.187 to 0.606) Estimated Error 0.1075"

[1] "75.2% posterior probability of decrease "

Urine/renal Source

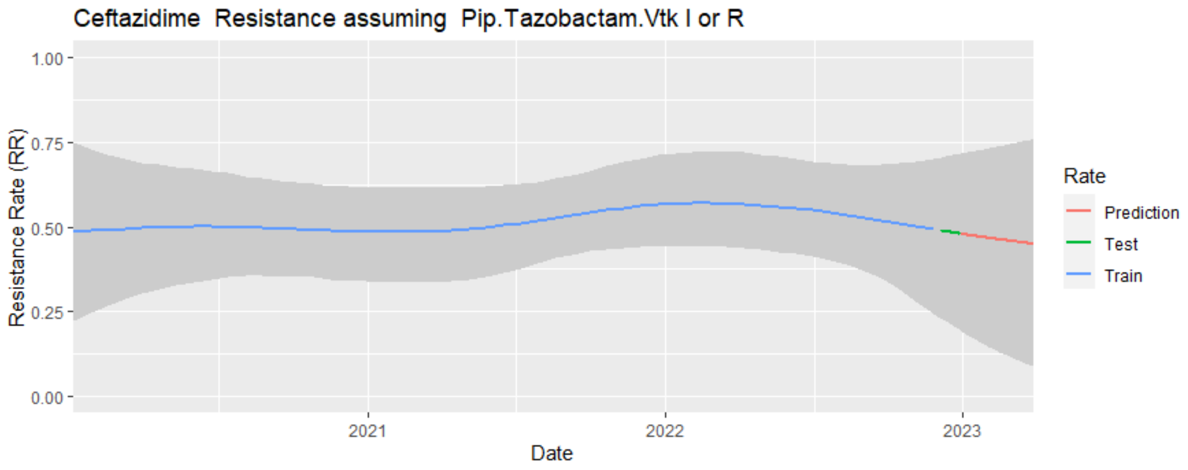

[1] "2020-01-06 Mid 0.486 (95%CI 0.220 to 0.750) Estimated Error 0.1335"

[1] "2022-12-06 Mid 0.488 (95%CI 0.228 to 0.706) Estimated Error 0.1218"

[1] "2022-12-28 Mid 0.479 (95%CI 0.195 to 0.715) Estimated Error 0.1338"

"48.4% posterior probability of decrease "

GI Source

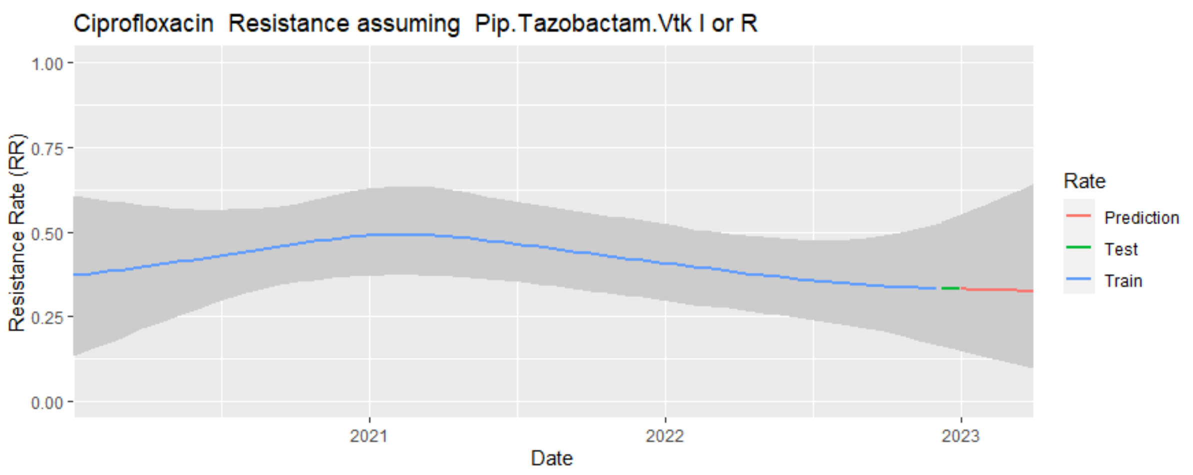

[1] "2020-01-01 Mid 0.370 (95%CI 0.133 to 0.608) Estimated Error 0.1245"

[1] "2022-12-07 Mid 0.332 (95%CI 0.160 to 0.528) Estimated Error 0.0945"

[1] "2022-12-30 Mid 0.330 (95%CI 0.147 to 0.548) Estimated Error 0.1029"

"59.4% posterior probability of decrease "

Urine/renal Source

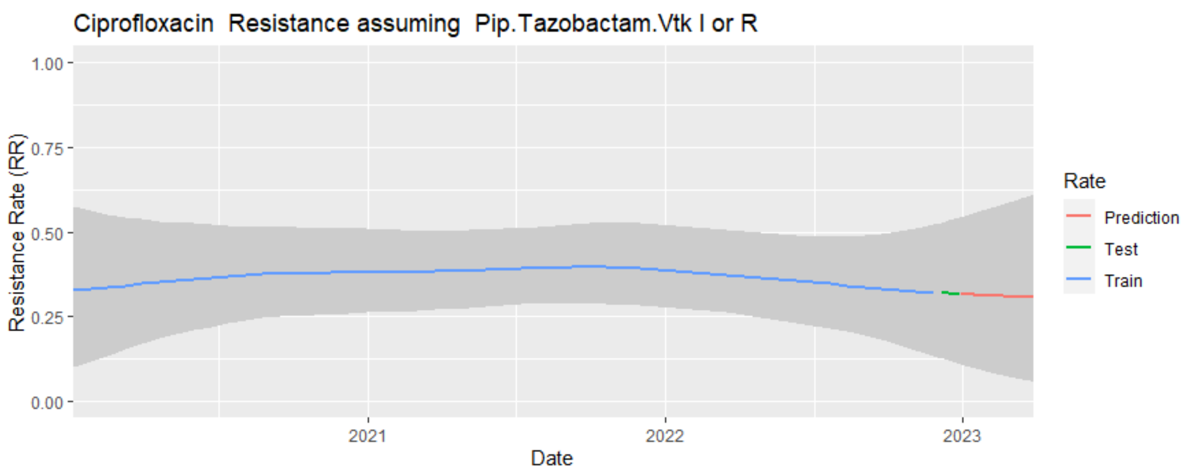

[1] "2020-01-06 Mid 0.326 (95%CI 0.099 to 0.574) Estimated Error 0.1231"

[1] "2022-12-06 Mid 0.319 (95%CI 0.125 to 0.527) Estimated Error 0.1033"

[1] "2022-12-28 Mid 0.316 (95%CI 0.108 to 0.542) Estimated Error 0.1107"

"51.0% posterior probability of decrease "

GI Source

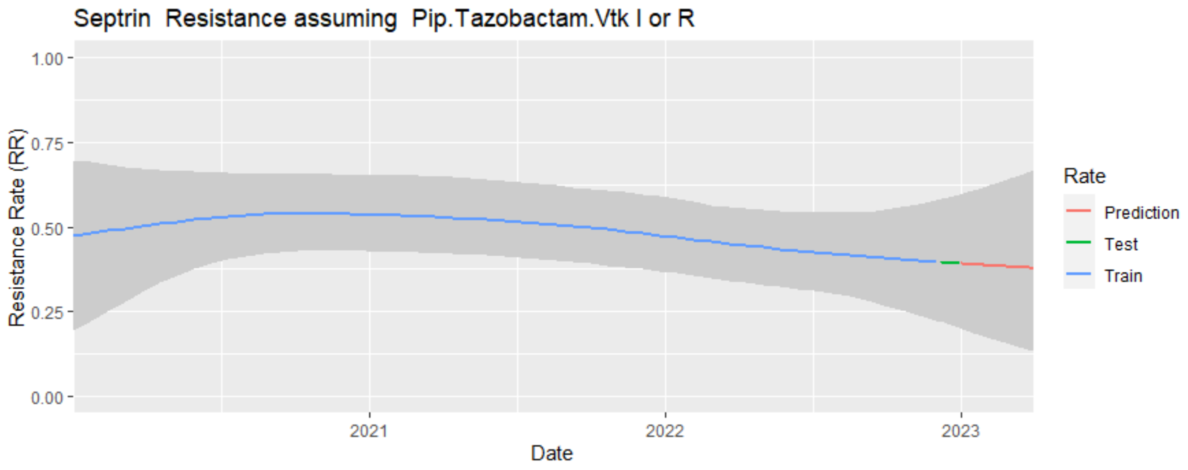

[1] "2020-01-01 Mid 0.475 (95%CI 0.193 to 0.699) Estimated Error 0.1309"

[1] "2022-12-07 Mid 0.395 (95%CI 0.219 to 0.581) Estimated Error 0.0929"

[1] "2022-12-30 Mid 0.391 (95%CI 0.199 to 0.594) Estimated Error 0.1009"

"70.2% posterior probability of decrease "

Urine/renal Source

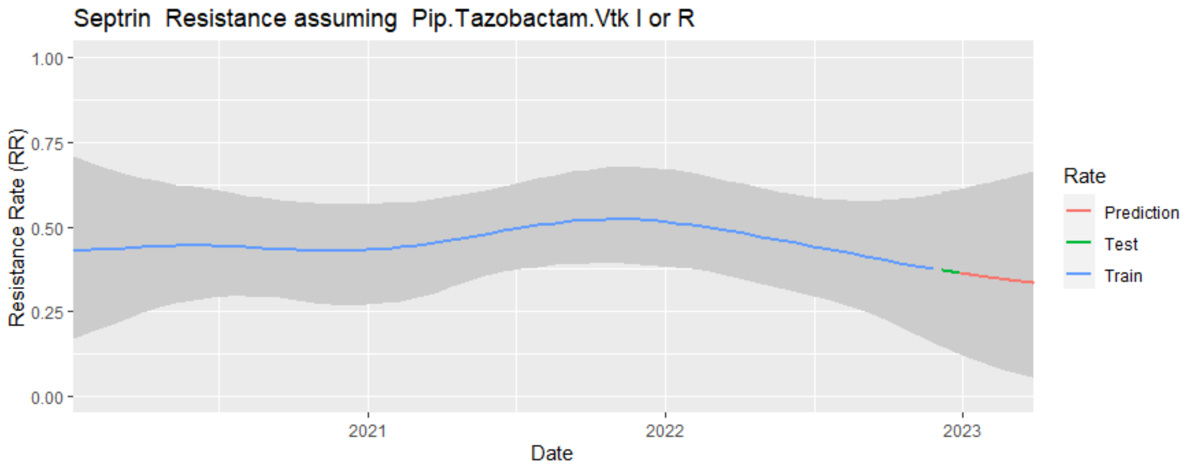

[1] "2020-01-06 Mid 0.428 (95%CI 0.169 to 0.707) Estimated Error 0.1382"

[1] "2022-12-06 Mid 0.371 (95%CI 0.145 to 0.602) Estimated Error 0.1183"

[1] "2022-12-28 Mid 0.362 (95%CI 0.122 to 0.611) Estimated Error 0.1280"

"61.5% posterior probability of decrease "

GI Source

Antibiotic resistance rate over time assuming ceftazidime resistance

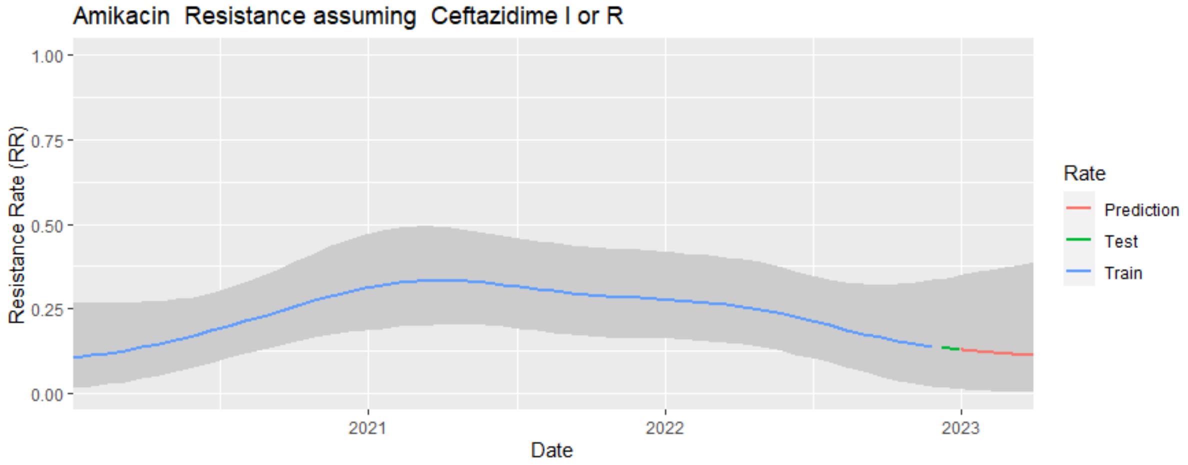

[1] "2020-01-03 Mid 0.105 (95%CI 0.014 to 0.271) Estimated Error 0.0679"

[1] "2022-12-07 Mid 0.134 (95%CI 0.015 to 0.336) Estimated Error 0.0849"

[1] "2022-12-30 Mid 0.127 (95%CI 0.010 to 0.348) Estimated Error 0.0893"

"38.5% posterior probability of decrease "

Urine/renal Source

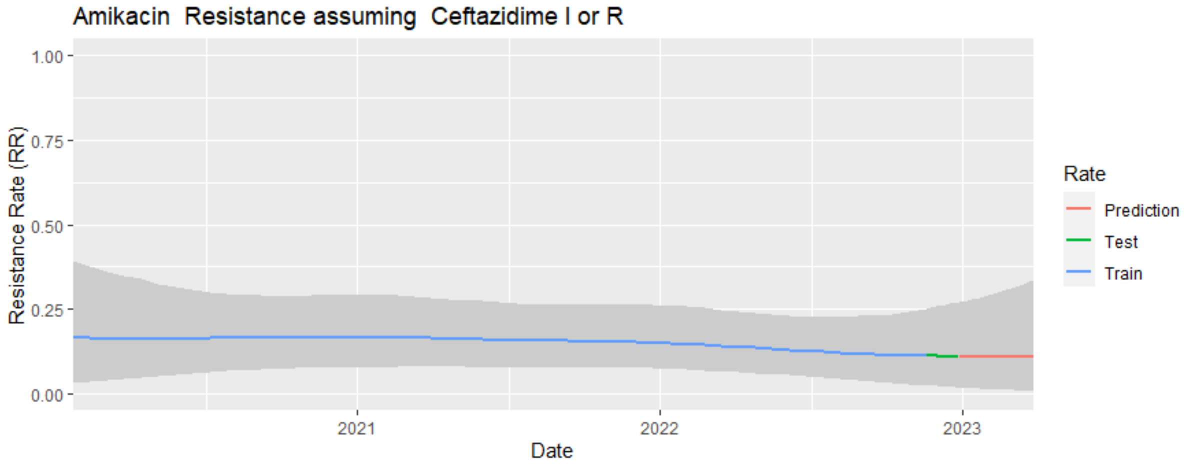

[1] "2020-01-24 Mid 0.164 (95%CI 0.029 to 0.394) Estimated Error 0.0957"

[1] "2022-11-17 Mid 0.111 (95%CI 0.023 to 0.250) Estimated Error 0.0594"

[1] "2022-12-26 Mid 0.109 (95%CI 0.016 to 0.268) Estimated Error 0.0666"

"66.6% posterior probability of decrease "

GI Source

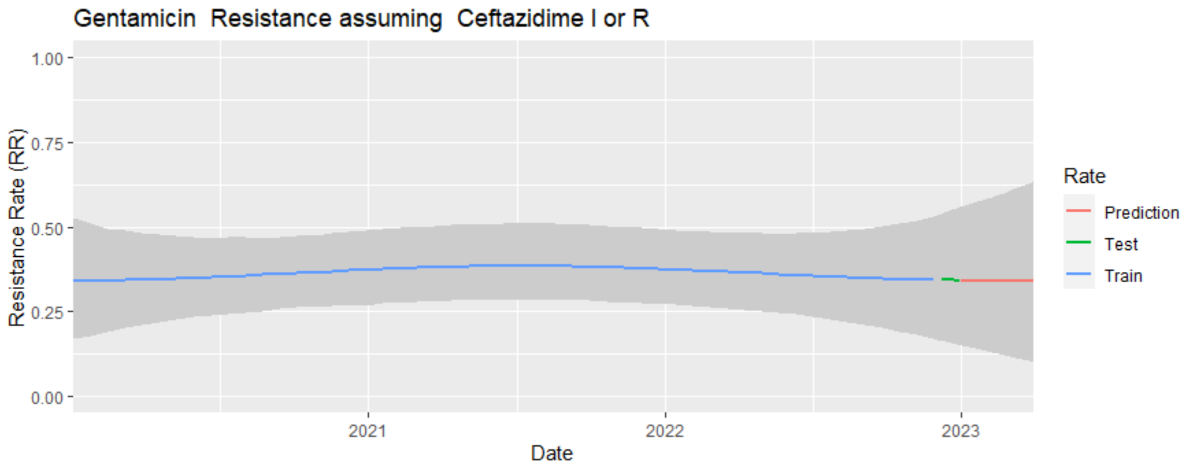

[1] "2020-01-03 Mid 0.339 (95%CI 0.168 to 0.526) Estimated Error 0.0917"

[1] "2022-12-07 Mid 0.342 (95%CI 0.163 to 0.539) Estimated Error 0.0965"

[1] "2022-12-30 Mid 0.341 (95%CI 0.149 to 0.557) Estimated Error 0.1037"

"48.5% posterior probability of decrease "

Urine/renal Source

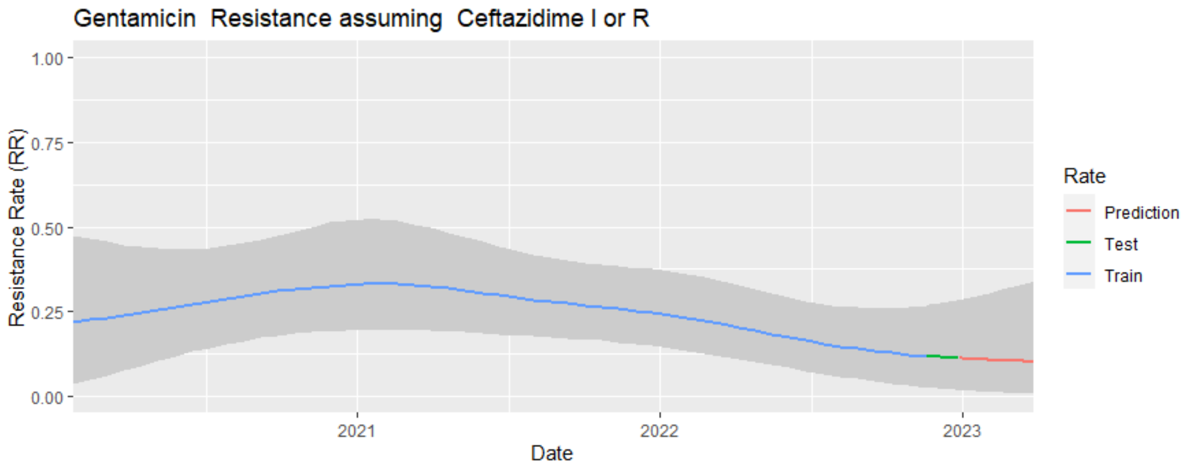

[1] "2020-01-24 Mid 0.218 (95%CI 0.036 to 0.476) Estimated Error 0.1182"

[1] "2022-11-17 Mid 0.118 (95%CI 0.024 to 0.268) Estimated Error 0.0647"

[1] "2022-12-26 Mid 0.111 (95%CI 0.016 to 0.282) Estimated Error 0.0716"

"78.1% posterior probability of decrease "

GI Source

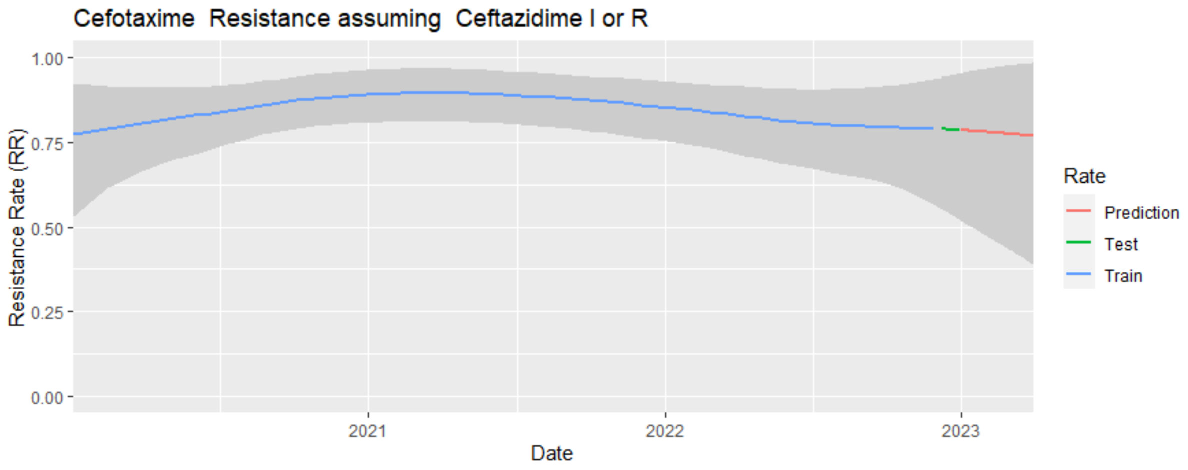

[1] "2020-01-03 Mid 0.774 (95%CI 0.531 to 0.925) Estimated Error 0.1028"

[1] "2022-12-07 Mid 0.789 (95%CI 0.552 to 0.942) Estimated Error 0.1009"

[1] "2022-12-30 Mid 0.786 (95%CI 0.520 to 0.953) Estimated Error 0.1120"

"46.4% posterior probability of decrease "

Urine/renal Source

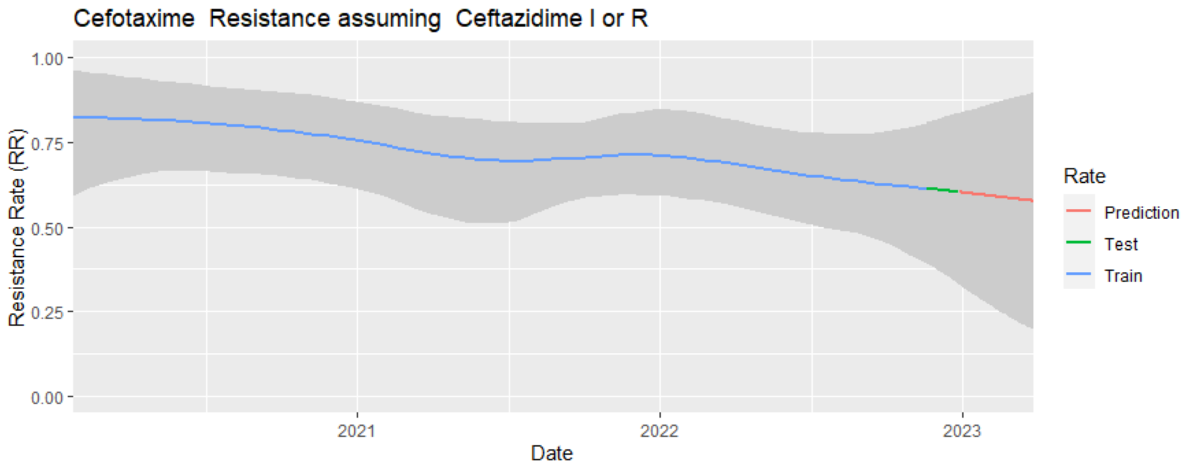

[1] "2020-01-24 Mid 0.824 (95%CI 0.592 to 0.965) Estimated Error 0.0958"

[1] "2022-11-17 Mid 0.614 (95%CI 0.393 to 0.808) Estimated Error 0.1047"

[1] "2022-12-26 Mid 0.603 (95%CI 0.332 to 0.836) Estimated Error 0.1261"

"92.7% posterior probability of decrease "

GI Source

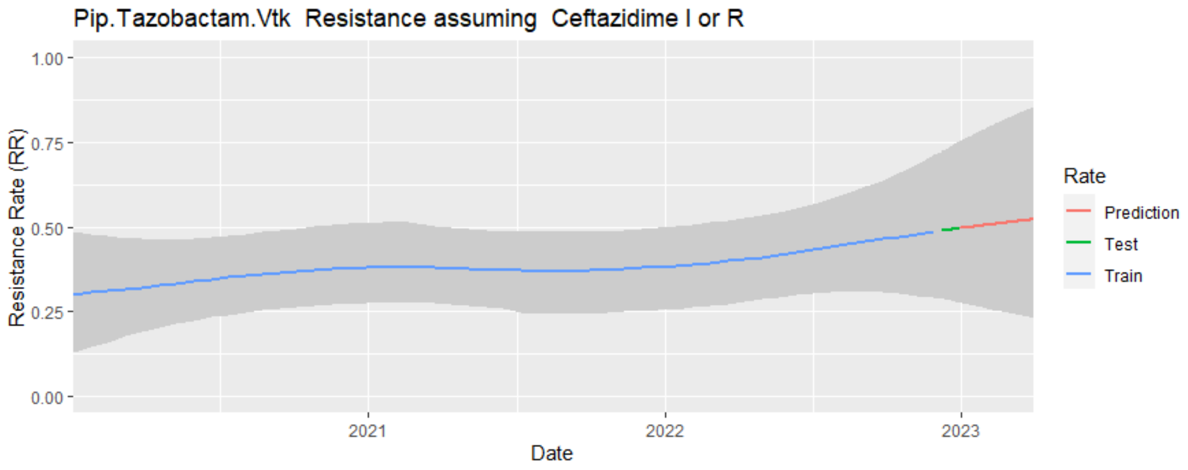

[1] "2020-01-03 Mid 0.301 (95%CI 0.128 to 0.488) Estimated Error 0.0911"

[1] "2022-12-07 Mid 0.488 (95%CI 0.286 to 0.722) Estimated Error 0.1116"

[1] "2022-12-30 Mid 0.495 (95%CI 0.275 to 0.753) Estimated Error 0.1213"

"10.3% posterior probability of decrease "

Urine/renal Source

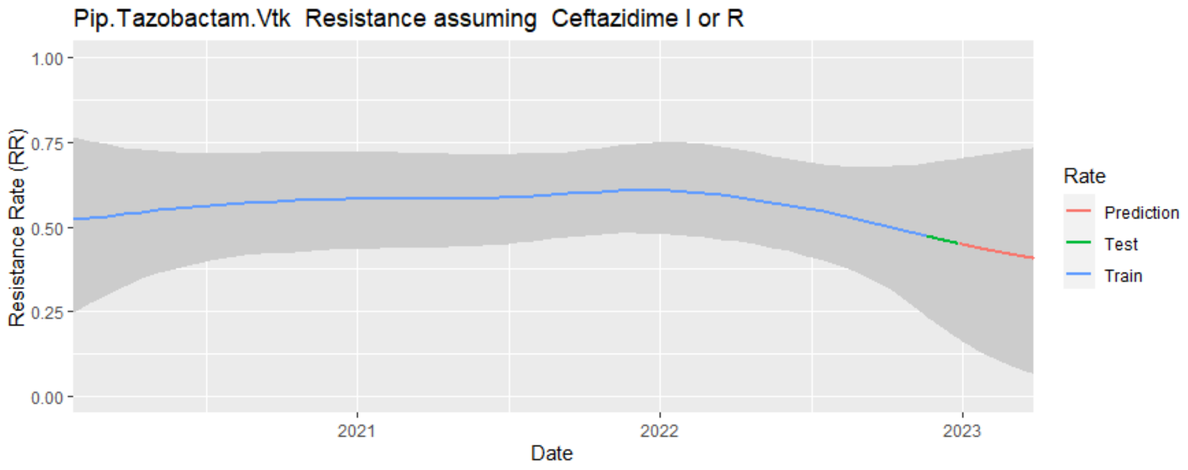

[1] "2020-01-24 Mid 0.521 (95%CI 0.245 to 0.764) Estimated Error 0.1344"

[1] "2022-11-17 Mid 0.472 (95%CI 0.236 to 0.688) Estimated Error 0.1166"

[1] "2022-12-26 Mid 0.450 (95%CI 0.169 to 0.700) Estimated Error 0.1379"

"60.9% posterior probability of decrease "

GI Source

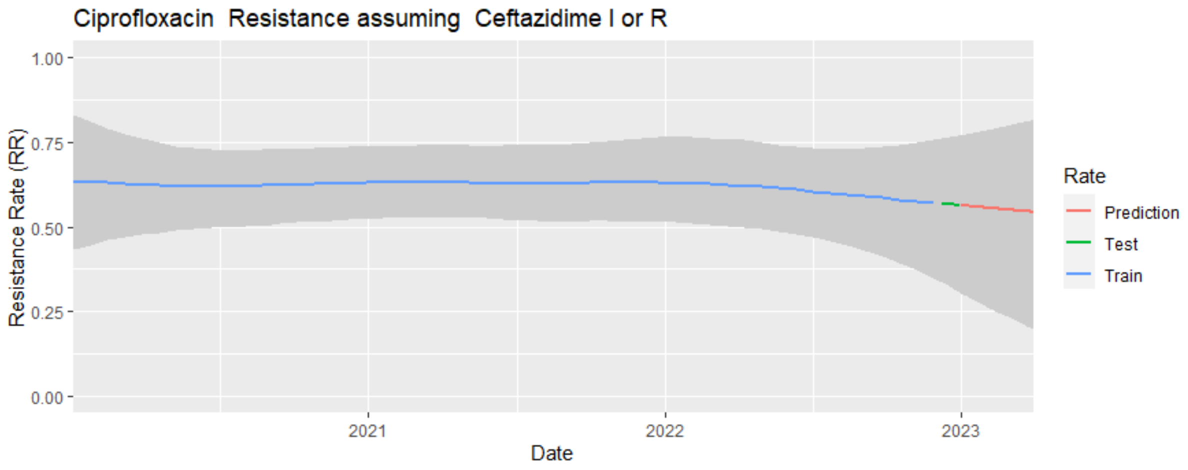

[1] "2020-01-03 Mid 0.635 (95%CI 0.433 to 0.830) Estimated Error 0.0991"

[1] "2022-12-07 Mid 0.569 (95%CI 0.336 to 0.761) Estimated Error 0.1083"

[1] "2022-12-30 Mid 0.564 (95%CI 0.305 to 0.769) Estimated Error 0.1181"

"65.9% posterior probability of decrease "

Urine/renal Source

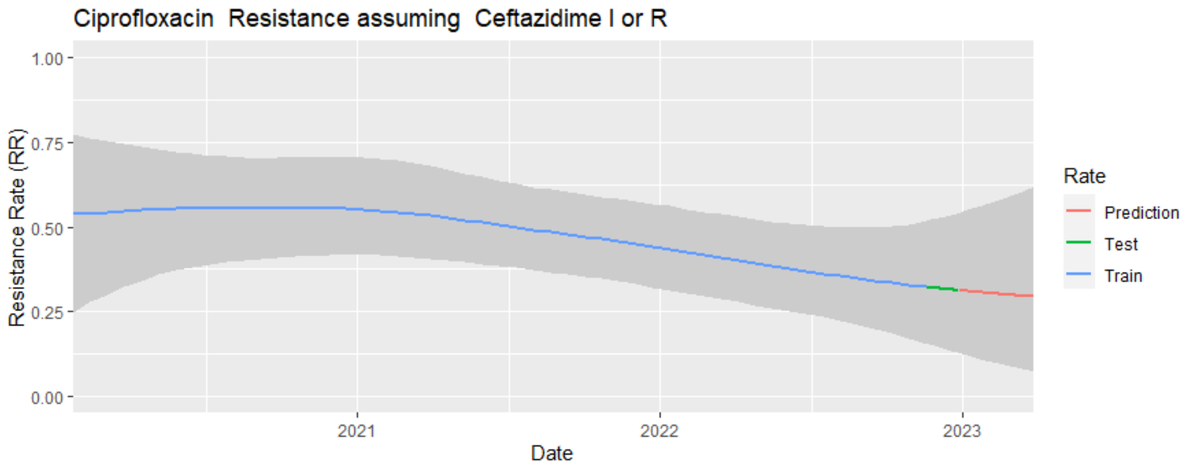

[1] "2020-01-24 Mid 0.537 (95%CI 0.247 to 0.774) Estimated Error 0.1355"

[1] "2022-11-17 Mid 0.322 (95%CI 0.154 to 0.518) Estimated Error 0.0925"

[1] "2022-12-26 Mid 0.312 (95%CI 0.126 to 0.539) Estimated Error 0.1054"

"90.3% posterior probability of decrease "

GI Source

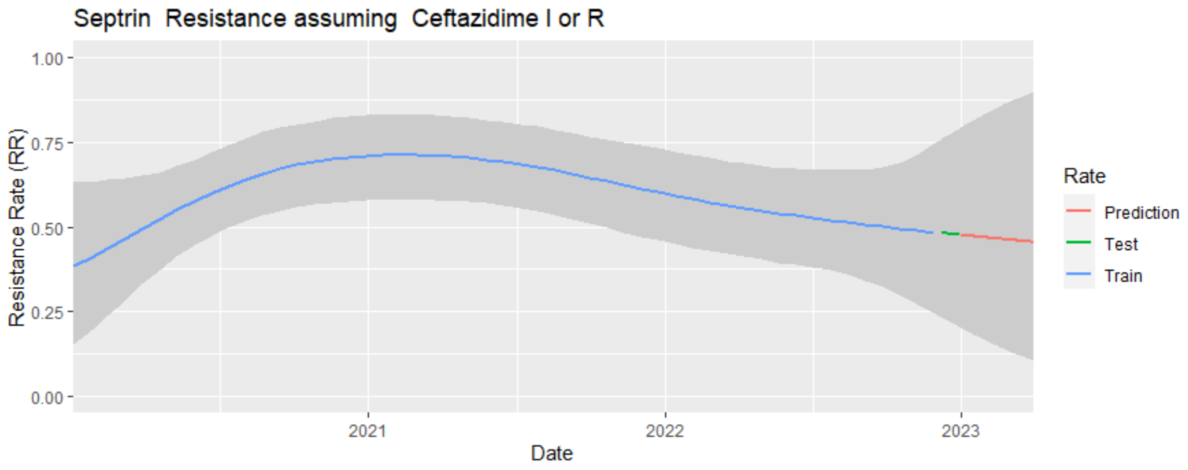

[1] "2020-01-03 Mid 0.383 (95%CI 0.152 to 0.633) Estimated Error 0.1267"

[1] "2022-12-07 Mid 0.482 (95%CI 0.232 to 0.759) Estimated Error 0.1332"

[1] "2022-12-30 Mid 0.476 (95%CI 0.202 to 0.791) Estimated Error 0.1488"

"31.1% posterior probability of decrease "

Urine/renal Source

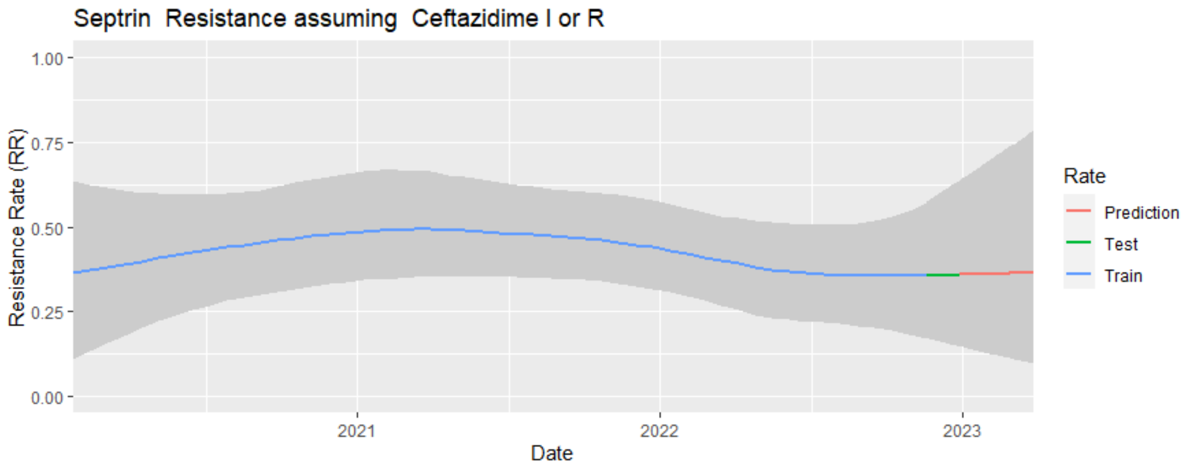

[1] "2020-01-24 Mid 0.365 (95%CI 0.109 to 0.635) Estimated Error 0.1392"

[1] "2022-11-17 Mid 0.356 (95%CI 0.169 to 0.573) Estimated Error 0.1039"

[1] "2022-12-26 Mid 0.358 (95%CI 0.146 to 0.633) Estimated Error 0.1243"

"52.6% posterior probability of decrease "

GI Source

Supplementary Material (EA Tables)

Posterior probability of resistance (Mid) with upper and lower 95% credible intervals

		Meropenem			Pip.Taz.			Ceftazidime			Cefotaxime.			Cefuroxime	
**Initial.Antibiotic**	**Mid**	**Upper**	**Lower**	**Mid**	**Upper**	**Lower**	**Mid**	**Upper**	**Lower**	**Mid**	**Upper**	**Lower**	**Mid**	**Upper**	**Lower**
**Meropenem**	NA	NA	NA	0.67	0.83	0.47	0.62	0.78	0.43	0.89	0.99	0.62	0.95	1.00	0.82
**Pip.Taz**	0.12	0.18	0.07	NA	NA	NA	0.47	0.56	0.38	0.44	0.53	0.33	0.63	0.71	0.53
**Ceftazidime**	0.13	0.21	0.07	0.57	0.68	0.43	NA	NA	NA	0.78	0.86	0.67	0.97	0.99	0.93
**Cefotaxime**	0.12	0.18	0.08	0.34	0.44	0.24	0.51	0.59	0.44	NA	NA	NA	0.99	1.00	0.98
**Cefuroxime**	0.07	0.11	0.05	0.29	0.36	0.22	0.36	0.42	0.31	0.57	0.62	0.52	NA	NA	NA
**Gentamicin**	0.15	0.28	0.07	0.46	0.58	0.33	0.45	0.59	0.35	0.45	0.59	0.35	0.58	0.71	0.47
**Amikacin**	0.24	0.46	0.11	0.65	0.89	0.44	0.67	0.88	0.47	0.74	0.90	0.58	0.81	0.94	0.67
**Coamoxiclav**	0.05	0.07	0.03	0.26	0.32	0.21	0.20	0.24	0.17	0.32	0.37	0.27	0.53	0.59	0.47
**Ciprofloxacin**	0.03	0.07	0.01	0.34	0.45	0.23	0.41	0.50	0.33	0.42	0.52	0.33	0.63	0.74	0.54
**Septrin**	0.04	0.07	0.02	0.18	0.23	0.13	0.18	0.22	0.14	0.42	0.49	0.34	0.50	0.57	0.43

		Gentamicin			Amikacin			Coamoxiclav			Ciprofloxacin			Septrin.	
**Initial.Antibiotic**	**Mid**	**Upper**	**Lower**	**Mid**	**Upper**	**Lower**	**Mid**	**Upper**	**Lower**	**Mid**	**Upper**	**Lower**	**Mid**	**Upper**	**Lower**
**Meropenem**	0.45	0.64	0.28	0.41	0.62	0.23	0.99	1.00	0.94	0.23	0.45	0.09	0.58	0.75	0.39
**Pip.Taz**	0.27	0.34	0.18	0.18	0.25	0.11	0.91	0.96	0.84	0.31	0.40	0.21	0.37	0.45	0.27
**Ceftazidime**	0.33	0.41	0.25	0.20	0.30	0.12	0.87	0.92	0.80	0.45	0.54	0.35	0.43	0.53	0.33
**Cefotaxime**	0.21	0.27	0.16	0.16	0.23	0.10	0.90	0.94	0.83	0.29	0.38	0.22	0.67	0.75	0.58
**Cefuroxime**	0.15	0.20	0.12	0.10	0.15	0.06	0.85	0.89	0.80	0.24	0.31	0.19	0.46	0.52	0.39
**Gentamicin**	NA	NA	NA	0.23	0.37	0.11	0.82	0.89	0.73	0.55	0.66	0.43	0.58	0.71	0.45
**Amikacin**	0.61	0.78	0.41	NA	NA	NA	0.85	0.94	0.71	0.66	0.86	0.41	0.45	0.64	0.24
**Coamoxiclav**	0.13	0.17	0.10	0.06	0.09	0.03	NA	NA	NA	0.17	0.21	0.13	0.44	0.48	0.39
**Ciprofloxacin**	0.35	0.43	0.27	0.16	0.25	0.08	0.72	0.80	0.64	NA	NA	NA	0.58	0.67	0.49
**Septrin**	0.17	0.22	0.12	0.05	0.09	0.02	0.78	0.82	0.73	0.24	0.30	0.19	NA	NA	NA

All Patients

		Meropenem			Pip.Taz.			Ceftazidime			Cefotaxime.			Cefuroxime	
**Initial.Antibioti**	**Mid**	**Upper**	**Lower**	**Mid**	**Upper**	**Lower**	**Mid**	**Upper**	**Lower**	**Mid**	**Upper**	**Lower**	**Mid**	**Upper**	**Lower**
**Meropenem**	NA	NA	NA	0.86	1.00	0.43	0.84	1.00	0.40	0.95	1.00	0.51	0.95	1.00	0.52
**Pip.Taz**	0.10	0.23	0.02	NA	NA	NA	0.62	0.83	0.36	0.65	0.83	0.41	0.82	0.94	0.63
**Ceftazidime**	0.13	0.32	0.03	0.86	0.98	0.65	NA	NA	NA	0.89	0.98	0.72	0.97	1.00	0.88
**Cefotaxime**	0.13	0.27	0.04	0.65	0.80	0.44	0.66	0.82	0.43	NA	NA	NA	1.00	1.00	0.97
**Cefuroxime**	0.09	0.20	0.03	0.55	0.69	0.38	0.49	0.64	0.33	0.68	0.82	0.53	NA	NA	NA
**Gentamicin**	0.05	0.22	0.00	0.42	0.74	0.13	0.36	0.68	0.06	0.34	0.64	0.06	0.45	0.75	0.13
**Amikacin**	0.19	0.64	0.01	0.64	0.96	0.20	0.46	0.90	0.05	0.66	0.97	0.19	0.62	0.97	0.12
**Coamoxiclav**	0.06	0.13	0.02	0.41	0.53	0.29	0.30	0.42	0.18	0.43	0.57	0.29	0.63	0.76	0.50
**Ciprofloxacin**	0.02	0.10	0.00	0.43	0.75	0.16	0.55	0.85	0.25	0.43	0.74	0.14	0.53	0.82	0.22
**Septrin**	0.09	0.23	0.02	0.23	0.42	0.08	0.20	0.37	0.07	0.56	0.76	0.37	0.59	0.78	0.41

		Gentamicin			Amikacin			Coamoxiclav			Ciprofloxacin			Septrin.	
**Initial.Antibioti**	**Mid**	**Upper**	**Lower**	**Mid**	**Upper**	**Lower**	**Mid**	**Upper**	**Lower**	**Mid**	**Upper**	**Lower**	**Mid**	**Upper**	**Lower**
**Meropenem**	0.26	0.76	0.01	0.24	0.86	0.00	0.95	1.00	0.53	0.04	0.38	0.00	0.18	0.68	0.00
**Pip.Taz**	0.19	0.36	0.07	0.14	0.31	0.04	0.94	1.00	0.79	0.17	0.34	0.06	0.23	0.42	0.10
**Ceftazidime**	0.24	0.45	0.06	0.17	0.40	0.04	0.98	1.00	0.88	0.28	0.53	0.11	0.30	0.70	0.11
**Cefotaxime**	0.17	0.32	0.05	0.14	0.31	0.04	0.98	1.00	0.91	0.18	0.33	0.06	0.50	0.70	0.32
**Cefuroxime**	0.14	0.26	0.04	0.10	0.22	0.03	0.99	1.00	0.94	0.14	0.26	0.05	0.37	0.55	0.24
**Gentamicin**	NA	NA	NA	0.24	0.59	0.03	0.80	0.97	0.46	0.43	0.72	0.16	0.50	0.81	0.18
**Amikacin**	0.93	1.00	0.60	NA	NA	NA	0.62	0.97	0.13	0.23	0.69	0.01	0.25	0.72	0.02
**Coamoxiclav**	0.15	0.24	0.07	0.07	0.15	0.02	NA	NA	NA	0.11	0.20	0.04	0.37	0.52	0.25
**Ciprofloxacin**	0.57	0.85	0.26	0.18	0.52	0.02	0.74	0.97	0.40	NA	NA	NA	0.37	0.70	0.07
**Septrin**	0.27	0.45	0.12	0.07	0.21	0.01	0.86	0.96	0.71	0.13	0.30	0.03	NA	NA	NA

ICU Patients

		Meropenem			Pip.Taz.			Ceftazidime			Cefotaxime.			Cefuroxime	
**Initial.Antibiotic**	**Mid**	**Upper**	**Lower**	**Mid**	**Upper**	**Lower**	**Mid**	**Upper**	**Lower**	**Mid**	**Upper**	**Lower**	**Mid**	**Upper**	**Lower**
**Meropenem**	NA	NA	NA	0.93	1.00	0.71	0.93	1.00	0.72	0.97	1.00	0.71	0.97	1.00	0.72
**Pip.Taz**	0.37	0.66	0.16	NA	NA	NA	0.62	0.82	0.38	0.58	0.81	0.33	0.72	0.91	0.45
**Ceftazidime**	0.42	0.66	0.21	0.77	0.92	0.54	NA	NA	NA	0.88	0.98	0.70	0.99	1.00	0.94
**Cefotaxime**	0.42	0.68	0.21	0.62	0.82	0.40	0.71	0.90	0.45	NA	NA	NA	0.99	1.00	0.95
**Cefuroxime**	0.26	0.46	0.11	0.46	0.64	0.24	0.52	0.72	0.30	0.63	0.81	0.44	NA	NA	NA
**Gentamicin**	0.56	0.84	0.20	0.80	0.97	0.50	0.72	0.95	0.42	0.68	0.92	0.37	0.74	0.95	0.43
**Amikacin**	0.41	0.80	0.11	0.73	0.95	0.39	0.66	0.93	0.32	0.64	0.91	0.31	0.75	0.97	0.40
**Coamoxiclav**	0.21	0.41	0.09	0.48	0.67	0.30	0.36	0.56	0.19	0.46	0.66	0.30	0.66	0.82	0.49
**Ciprofloxacin**	0.14	0.43	0.01	0.40	0.67	0.14	0.39	0.68	0.13	0.44	0.71	0.17	0.49	0.79	0.13
**Septrin**	0.24	0.52	0.07	0.36	0.60	0.15	0.35	0.61	0.14	0.59	0.79	0.39	0.61	0.80	0.37

		Gentamicin			Amikacin			Coamoxiclav			Ciprofloxacin			Septrin.	
**Initial.Antibiotic**	**Mid**	**Upper**	**Lower**	**Mid**	**Upper**	**Lower**	**Mid**	**Upper**	**Lower**	**Mid**	**Upper**	**Lower**	**Mid**	**Upper**	**Lower**
**Meropenem**	0.70	0.94	0.36	0.54	0.87	0.19	0.97	1.00	0.69	0.37	0.85	0.03	0.39	0.74	0.10
**Pip.Taz**	0.32	0.55	0.12	0.30	0.56	0.09	0.87	0.98	0.62	0.31	0.64	0.09	0.42	0.75	0.16
**Ceftazidime**	0.39	0.64	0.18	0.38	0.62	0.16	0.92	0.99	0.75	0.35	0.62	0.13	0.39	0.64	0.16
**Cefotaxime**	0.28	0.54	0.11	0.29	0.52	0.09	0.91	0.99	0.72	0.27	0.52	0.07	0.59	0.82	0.33
**Cefuroxime**	0.17	0.34	0.07	0.21	0.39	0.07	0.78	0.93	0.52	0.17	0.35	0.04	0.40	0.59	0.22
**Gentamicin**	NA	NA	NA	0.72	0.93	0.41	0.90	1.00	0.63	0.45	0.82	0.09	0.32	0.65	0.06
**Amikacin**	0.71	0.96	0.36	NA	NA	NA	0.69	0.97	0.25	0.68	0.96	0.18	0.03	0.22	0.00
**Coamoxiclav**	0.18	0.35	0.08	0.19	0.36	0.07	NA	NA	NA	0.29	0.62	0.12	0.44	0.64	0.26
**Ciprofloxacin**	0.22	0.50	0.05	0.19	0.53	0.01	0.71	0.92	0.38	NA	NA	NA	0.80	1.00	0.43
**Septrin**	0.11	0.26	0.02	0.02	0.12	0.00	0.86	0.97	0.66	0.40	0.71	0.18	NA	NA	NA

Haemato-oncology Patients

		Meropenem			Pip.Taz.			Ceftazidime			Cefotaxime.			Cefuroxime	
**Initial.Antibiotic**	**Mid**	**Upper**	**Lower**	**Mid**	**Upper**	**Lower**	**Mid**	**Upper**	**Lower**	**Mid**	**Upper**	**Lower**	**Mid**	**Upper**	**Lower**
**Meropenem**	NA	NA	NA	x	x	x	x	x	x	x	x	x	x	x	x
**Pip.Taz**	0.01	0.07	0.00	NA	NA	NA	0.36	0.57	0.20	0.29	0.48	0.14	0.48	0.68	0.31
**Ceftazidime**	0.01	0.06	0.00	0.49	0.72	0.29	NA	NA	NA	0.79	0.94	0.56	0.98	1.00	0.88
**Cefotaxime**	0.06	0.20	0.00	0.26	0.44	0.13	0.50	0.69	0.34	NA	NA	NA	1.00	1.00	0.97
**Cefuroxime**	0.03	0.13	0.00	0.26	0.41	0.14	0.37	0.54	0.24	0.56	0.70	0.40	NA	NA	NA
**Gentamicin**	0.01	0.09	0.00	0.41	0.63	0.21	0.34	0.58	0.17	0.32	0.53	0.15	0.51	0.73	0.30
**Amikacin**	0.02	0.18	0.00	0.44	0.79	0.14	0.57	0.88	0.26	0.63	0.89	0.31	0.72	0.93	0.39
**Coamoxiclav**	0.02	0.07	0.00	0.23	0.32	0.14	0.14	0.22	0.08	0.25	0.35	0.16	0.41	0.51	0.30
**Ciprofloxacin**	0.02	0.12	0.00	0.30	0.51	0.14	0.38	0.56	0.22	0.38	0.55	0.22	0.58	0.76	0.40
**Septrin**	0.03	0.10	0.00	0.16	0.26	0.08	0.14	0.23	0.08	0.31	0.42	0.20	0.39	0.50	0.26

		Gentamicin			Amikacin			Coamoxiclav			Ciprofloxacin			Septrin.	
**Initial.Antibiotic**	**Mid**	**Upper**	**Lower**	**Mid**	**Upper**	**Lower**	**Mid**	**Upper**	**Lower**	**Mid**	**Upper**	**Lower**	**Mid**	**Upper**	**Lower**
**Meropenem**	x	x	x	x	x	x	x	x	x	x	x	x	x	x	x
**Pip.Taz**	0.29	0.47	0.12	0.13	0.28	0.03	0.77	0.91	0.55	0.33	0.53	0.16	0.40	0.59	0.22
**Ceftazidime**	0.34	0.55	0.16	0.14	0.34	0.02	0.70	0.87	0.46	0.57	0.78	0.33	0.48	0.73	0.24
**Cefotaxime**	0.21	0.36	0.08	0.10	0.24	0.01	0.78	0.90	0.59	0.36	0.53	0.20	0.66	0.84	0.43
**Cefuroxime**	0.19	0.30	0.09	0.08	0.18	0.02	0.74	0.85	0.57	0.33	0.47	0.21	0.49	0.65	0.33
**Gentamicin**	NA	NA	NA	0.13	0.30	0.02	0.73	0.90	0.51	0.58	0.82	0.37	0.68	0.90	0.42
**Amikacin**	0.56	0.85	0.27	NA	NA	NA	0.66	0.93	0.24	0.63	0.91	0.23	0.38	0.75	0.08
**Coamoxiclav**	0.14	0.22	0.07	0.04	0.09	0.01	NA	NA	NA	0.19	0.27	0.11	0.57	0.71	0.43
**Ciprofloxacin**	0.42	0.61	0.26	0.11	0.26	0.01	0.58	0.78	0.35	NA	NA	NA	0.48	0.68	0.29
**Septrin**	0.18	0.27	0.10	0.05	0.11	0.01	0.74	0.85	0.62	0.23	0.34	0.14	NA	NA	NA

Urine Source

		Meropenem			Pip.Taz.			Ceftazidime			Cefotaxime.			Cefuroxime	
**Initial.Antibiotic**	**Mid**	**Upper**	**Lower**	**Mid**	**Upper**	**Lower**	**Mid**	**Upper**	**Lower**	**Mid**	**Upper**	**Lower**	**Mid**	**Upper**	**Lower**
**Meropenem**	NA	NA	NA	0.05	0.51	0.00	0.11	0.72	0.00	0.93	1.00	0.35	0.93	1.00	0.36
**Pip.Taz**	0.05	0.16	0.00	NA	NA	NA	0.49	0.70	0.23	0.41	0.63	0.17	0.60	0.80	0.35
**Ceftazidime**	0.04	0.15	0.00	0.47	0.68	0.23	NA	NA	NA	0.61	0.80	0.39	0.92	0.99	0.77
**Cefotaxime**	0.11	0.25	0.02	0.38	0.59	0.17	0.64	0.82	0.44	NA	NA	NA	0.99	1.00	0.93
**Cefuroxime**	0.06	0.16	0.01	0.32	0.48	0.17	0.54	0.71	0.38	0.60	0.79	0.44	NA	NA	NA
**Gentamicin**	0.02	0.17	0.00	0.35	0.71	0.04	0.41	0.75	0.12	0.34	0.69	0.07	0.45	0.77	0.11
**Amikacin**	0.02	0.18	0.00	0.65	0.99	0.19	0.49	0.91	0.06	0.82	1.00	0.31	0.83	1.00	0.25
**Coamoxiclav**	0.04	0.09	0.01	0.28	0.39	0.18	0.29	0.40	0.19	0.29	0.41	0.19	0.46	0.57	0.33
**Ciprofloxacin**	0.02	0.09	0.00	0.38	0.61	0.18	0.44	0.66	0.24	0.60	0.93	0.32	0.71	0.94	0.47
**Septrin**	0.08	0.22	0.02	0.26	0.44	0.10	0.28	0.46	0.14	0.45	0.65	0.28	0.56	0.76	0.39

		Gentamicin			Amikacin			Coamoxiclav			Ciprofloxacin			Septrin.	
**Initial.Antibiotic**	**Mid**	**Upper**	**Lower**	**Mid**	**Upper**	**Lower**	**Mid**	**Upper**	**Lower**	**Mid**	**Upper**	**Lower**	**Mid**	**Upper**	**Lower**
**Meropenem**	0.10	0.73	0.00	0.01	0.06	0.00	0.94	1.00	0.36	0.17	0.65	0.00	0.96	1.00	0.56
**Pip.Taz**	0.14	0.32	0.02	0.18	0.40	0.05	0.94	1.00	0.77	0.32	0.52	0.13	0.37	0.60	0.15
**Ceftazidime**	0.12	0.27	0.02	0.11	0.25	0.02	0.89	0.97	0.74	0.32	0.52	0.14	0.35	0.57	0.17
**Cefotaxime**	0.18	0.36	0.05	0.19	0.37	0.06	0.88	0.97	0.72	0.43	0.67	0.24	0.56	0.77	0.33
**Cefuroxime**	0.12	0.23	0.04	0.11	0.22	0.03	0.81	0.91	0.67	0.30	0.49	0.16	0.44	0.68	0.27
**Gentamicin**	NA	NA	NA	0.14	0.42	0.00	0.95	1.00	0.75	0.41	0.77	0.08	0.68	0.94	0.36
**Amikacin**	0.29	0.74	0.01	NA	NA	NA	0.85	1.00	0.33	0.81	1.00	0.28	0.73	1.00	0.21
**Coamoxiclav**	0.12	0.21	0.05	0.06	0.13	0.02	NA	NA	NA	0.22	0.44	0.11	0.34	0.46	0.21
**Ciprofloxacin**	0.25	0.50	0.07	0.25	0.47	0.09	0.88	0.98	0.73	NA	NA	NA	0.54	0.74	0.32
**Septrin**	0.17	0.32	0.06	0.07	0.18	0.01	0.81	0.92	0.64	0.32	0.49	0.17	NA	NA	NA

GI source

		Meropenem			Pip.Taz.			Ceftazidime			Cefotaxime.			Cefuroxime	
**Initial.Antibiotic**	**Mid**	**Upper**	**Lower**	**Mid**	**Upper**	**Lower**	**Mid**	**Upper**	**Lower**	**Mid**	**Upper**	**Lower**	**Mid**	**Upper**	**Lower**
**Meropenem**	NA	NA	NA	0.90	1.00	0.59	0.88	1.00	0.57	0.98	1.00	0.78	0.98	1.00	0.78
**Pip.Taz**	0.34	0.58	0.14	NA	NA	NA	0.52	0.81	0.18	0.65	0.86	0.33	0.74	0.91	0.50
**Ceftazidime**	0.58	0.90	0.25	0.92	1.00	0.65	NA	NA	NA	0.92	1.00	0.69	0.90	1.00	0.68
**Cefotaxime**	0.37	0.67	0.15	0.67	0.89	0.40	0.57	0.82	0.34	NA	NA	NA	0.95	1.00	0.81
**Cefuroxime**	0.28	0.54	0.11	0.61	0.86	0.35	0.42	0.66	0.23	0.71	0.87	0.52	NA	NA	NA
**Gentamicin**	0.48	0.84	0.17	0.92	1.00	0.66	0.91	1.00	0.64	0.89	1.00	0.59	0.91	1.00	0.64
**Amikacin**	0.55	0.93	0.12	0.96	1.00	0.68	0.96	1.00	0.73	0.95	1.00	0.66	0.95	1.00	0.62
**Coamoxiclav**	0.16	0.31	0.06	0.49	0.68	0.30	0.28	0.44	0.15	0.44	0.61	0.26	0.54	0.73	0.32
**Ciprofloxacin**	0.44	0.86	0.06	0.86	1.00	0.42	0.83	1.00	0.41	0.91	1.00	0.53	0.94	1.00	0.60
**Septrin**	0.22	0.47	0.06	0.36	0.66	0.13	0.30	0.56	0.12	0.61	0.83	0.32	0.66	0.87	0.36

		Gentamicin			Amikacin			Coamoxiclav			Ciprofloxacin			Septrin.	
**Initial.Antibiotic**	**Mid**	**Upper**	**Lower**	**Mid**	**Upper**	**Lower**	**Mid**	**Upper**	**Lower**	**Mid**	**Upper**	**Lower**	**Mid**	**Upper**	**Lower**
**Pip.Taz**	0.45	0.70	0.22	0.28	0.55	0.09	0.99	1.00	0.95	0.26	0.50	0.09	0.31	0.54	0.11
**Ceftazidime**	0.82	0.99	0.46	0.66	0.96	0.23	0.99	1.00	0.90	0.44	0.89	0.13	0.43	0.72	0.14
**Cefotaxime**	0.52	0.83	0.22	0.39	0.74	0.10	0.99	1.00	0.96	0.24	0.52	0.07	0.51	0.77	0.22
**Cefuroxime**	0.41	0.71	0.16	0.27	0.57	0.07	0.98	1.00	0.89	0.17	0.39	0.05	0.42	0.64	0.18
**Gentamicin**	NA	NA	NA	0.47	0.87	0.12	0.86	1.00	0.51	0.37	0.84	0.06	0.49	0.80	0.15
**Amikacin**	0.99	1.00	0.85	NA	NA	NA	0.97	1.00	0.72	x	x	x	0.23	0.69	0.01
**Coamoxiclav**	0.19	0.35	0.09	0.14	0.31	0.03	NA	NA	NA	0.09	0.20	0.03	0.39	0.55	0.22
**Ciprofloxacin**	0.73	0.99	0.27	0.30	0.92	0.00	0.92	1.00	0.54	NA	NA	NA	0.67	0.97	0.22
**Septrin**	0.21	0.45	0.06	0.04	0.20	0.00	0.93	0.99	0.79	0.13	0.34	0.03	NA	NA	NA

Patients over 80 years

		Meropenem			Pip.Taz.			Ceftazidime			Cefotaxime.			Cefuroxime	
**Initial.Antibioti**	**Mid**	**Upper**	**Lower**	**Mid**	**Upper**	**Lower**	**Mid**	**Upper**	**Lower**	**Mid**	**Upper**	**Lower**	**Mid**	**Upper**	**Lower**
**Meropenem**	NA	NA	NA	0.90	1.00	0.59	0.88	1.00	0.57	0.98	1.00	0.78	0.98	1.00	0.78
**Pip.Taz**	0.34	0.58	0.14	NA	NA	NA	0.52	0.81	0.18	0.65	0.86	0.33	0.74	0.91	0.50
**Ceftazidime**	0.58	0.90	0.25	0.92	1.00	0.65	NA	NA	NA	0.92	1.00	0.69	0.90	1.00	0.68
**Cefotaxime**	0.37	0.67	0.15	0.67	0.89	0.40	0.57	0.82	0.34	NA	NA	NA	0.95	1.00	0.81
**Cefuroxime**	0.28	0.54	0.11	0.61	0.86	0.35	0.42	0.66	0.23	0.71	0.87	0.52	NA	NA	NA
**Gentamicin**	0.48	0.84	0.17	0.92	1.00	0.66	0.91	1.00	0.64	0.89	1.00	0.59	0.91	1.00	0.64
**Amikacin**	0.55	0.93	0.12	0.96	1.00	0.68	0.96	1.00	0.73	0.95	1.00	0.66	0.95	1.00	0.62
**Coamoxiclav**	0.16	0.31	0.06	0.49	0.68	0.30	0.28	0.44	0.15	0.44	0.61	0.26	0.54	0.73	0.32
**Ciprofloxacin**	0.44	0.86	0.06	0.86	1.00	0.42	0.83	1.00	0.41	0.91	1.00	0.53	0.94	1.00	0.60
**Septrin**	0.22	0.47	0.06	0.36	0.66	0.13	0.30	0.56	0.12	0.61	0.83	0.32	0.66	0.87	0.36

		Gentamicin			Amikacin			Coamoxiclav			Ciprofloxacin			Septrin.	
**Initial.Antibioti**	**Mid**	**Upper**	**Lower**	**Mid**	**Upper**	**Lower**	**Mid**	**Upper**	**Lower**	**Mid**	**Upper**	**Lower**	**Mid**	**Upper**	**Lower**
**Pip.Taz**	0.45	0.70	0.22	0.28	0.55	0.09	0.99	1.00	0.95	0.26	0.50	0.09	0.31	0.54	0.11
**Ceftazidime**	0.82	0.99	0.46	0.66	0.96	0.23	0.99	1.00	0.90	0.44	0.89	0.13	0.43	0.72	0.14
**Cefotaxime**	0.52	0.83	0.22	0.39	0.74	0.10	0.99	1.00	0.96	0.24	0.52	0.07	0.51	0.77	0.22
**Cefuroxime**	0.41	0.71	0.16	0.27	0.57	0.07	0.98	1.00	0.89	0.17	0.39	0.05	0.42	0.64	0.18
**Gentamicin**	NA	NA	NA	0.47	0.87	0.12	0.86	1.00	0.51	0.37	0.84	0.06	0.49	0.80	0.15
**Amikacin**	0.99	1.00	0.85	NA	NA	NA	0.97	1.00	0.72	x	x	x	0.23	0.69	0.01
**Coamoxiclav**	0.19	0.35	0.09	0.14	0.31	0.03	NA	NA	NA	0.09	0.20	0.03	0.39	0.55	0.22
**Ciprofloxacin**	0.73	0.99	0.27	0.30	0.92	0.00	0.92	1.00	0.54	NA	NA	NA	0.67	0.97	0.22
**Septrin**	0.21	0.45	0.06	0.04	0.20	0.00	0.93	0.99	0.79	0.13	0.34	0.03	NA	NA	NA

Patients Under 18
